# Evaluating the role of the nuclear microenvironment in gene function by population-based modeling

**DOI:** 10.1038/s41594-023-01036-1

**Published:** 2023-08-14

**Authors:** Asli Yildirim, Nan Hua, Lorenzo Boninsegna, Yuxiang Zhan, Guido Polles, Ke Gong, Shengli Hao, Wenyuan Li, Xianghong Jasmine Zhou, Frank Alber

**Affiliations:** 1 Institute for Quantitative and Computational Biosciences, University of California Los Angeles, Los Angeles, CA USA; 2 Department of Microbiology, Immunology, and Molecular Genetics, University of California Los Angeles, Los Angeles, CA USA; 3 Department of Quantitative and Computational Biology, University of Southern California, Los Angeles, CA USA; 4 Department of Pathology, David Geffen School of Medicine, University of California Los Angeles, Los Angeles, CA USA

**Keywords:** Computational biology and bioinformatics, Chromatin structure, Chromatin analysis, Chromatin, Nuclear organization

## Abstract

The nuclear folding of chromosomes relative to nuclear bodies is an integral part of gene function. Here, we demonstrate that population-based modeling—from ensemble Hi-C data—provides a detailed description of the nuclear microenvironment of genes and its role in gene function. We define the microenvironment by the subnuclear positions of genomic regions with respect to nuclear bodies, local chromatin compaction, and preferences in chromatin compartmentalization. These structural descriptors are determined in single-cell models, thereby revealing the structural variability between cells. We demonstrate that the microenvironment of a genomic region is linked to its functional potential in gene transcription, replication, and chromatin compartmentalization. Some chromatin regions feature a strong preference for a single microenvironment, due to association with specific nuclear bodies in most cells. Other chromatin shows high structural variability, which is a strong indicator of functional heterogeneity. Moreover, we identify specialized nuclear microenvironments, which distinguish chromatin in different functional states and reveal a key role of nuclear speckles in chromosome organization. We demonstrate that our method produces highly predictive three-dimensional genome structures, which accurately reproduce data from a variety of orthogonal experiments, thus considerably expanding the range of Hi-C data analysis.

## Abstract (web summary)

Here, using population-based modeling on ensemble Hi-C data, the authors provide an expansive overview of how the genic chromatin microenvironment influences its potential involvement in different functions, such as transcription, DNA replication, and chromatin compartmentalization. Their results unveil a key role of nuclear speckles in genome organization.

## Main

The spatial organization of eukaryotic genomes is linked to regulation of gene transcription, DNA replication, cell differentiation, and, upon malfunction, to cancer and other diseases^1,2^. Recent advances have led to a prolific development of improved technologies in live-cell and super-resolution microscopy^3–10^, as well as mapping technologies based on high-throughput sequencing^11–26^, for probing chromosome interactions and three-dimensional (3D) organization^27–30^. However, mapping the 3D nuclear locations of all genes simultaneously in single cells remains a major challenge. Several experimental technologies probe the mean distances (tyramide signal amplification sequencing (TSA-seq)^13^) or association frequencies (nucleolus-associated domain sequencing (NAD-seq)^31^; DNA adenine methyltransferase identification (DamID)^16^) of genes with nuclear speckles, lamina-associated domains (LADs), and nucleoli. However, these methods do not have the technical capacity to collect all this information simultaneously within the same cell, and the considerable cell-to-cell variability of chromosomal structures adds additional layers of complexity. Several multiplex fluorescence in situ hybridization (FISH) and super-resolution microscopy techniques have recently provided such information^5–7^. For instance, DNA- and RNA-multiplexed error-robust FISH (MERFISH) imaging has detected, within the same cells, the nuclear locations of 1,137 genes, together with the positions of nuclear speckles and nucleoli, as well as the amount of mRNA transcripts^6^. However, at this point, the amount of probed genomic DNA regions is still sparse, representing ~1% of entire genomes.

Here, we introduce an approach for modeling a population of single-cell 3D genome structures to describe the nuclear microenvironment of all genomic regions in single-cell models, defined by their nuclear locations relative to nuclear landmarks and nuclear compartments. Our aim is to evaluate the roles of the nuclear microenvironment and its cell-to-cell variability in chromatin function and identify characteristic nuclear microenvironments that distinguish chromatin in different functional states.

**Fig. 1:**
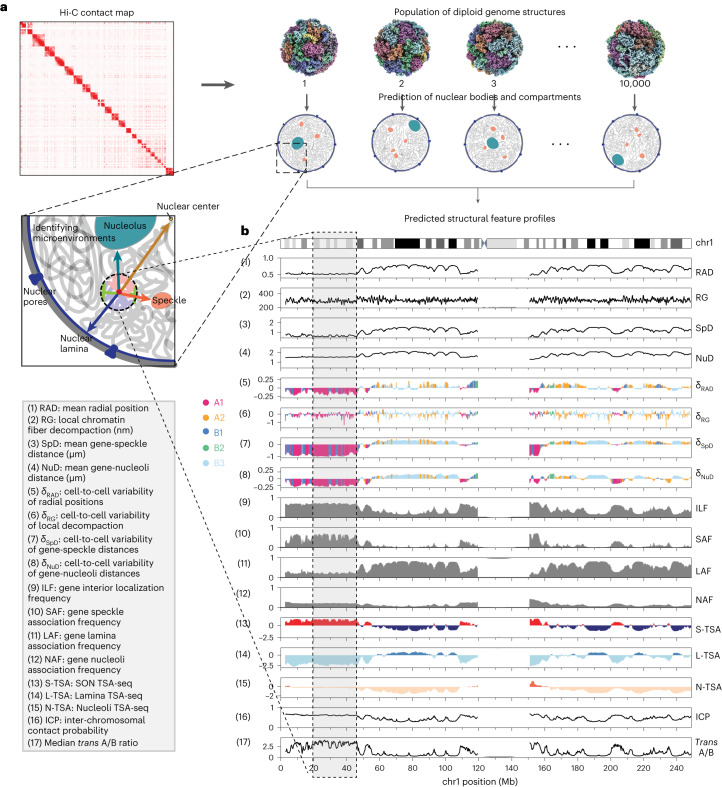
Microenvironment and structural features of genomic regions. **a**, Schematic depiction of our approach. A population of 10,000 genome structures is generated that is statistically consistent with the ensemble Hi-C data. Genome structures predict the locations of nuclear speckles, nucleoli, and the lamina-associated compartment, which serve as reference points to describe the global genome organization and define structural features. **b**, Seventeen structural features are calculated from the models that describe the nuclear microenvironment of each genomic region. Structural feature profiles for chromosome 1 are shown. Profiles for other chromosomes are shown in Supplementary Figs. 2–22.

We achieve this goal by using a population-based genome structure modeling approach, which takes Hi-C data to generate a population of diploid genome structures statistically consistent with it^32,33,34^. We demonstrate that our method produces—from Hi-C data alone—highly predictive genome structures, which predict with high correlation the cytological distances of genomic regions to nuclear speckles and lamina from SON TSA-seq^13^ and lamin-B1 TSA-seq^13^ experiments, contact probabilities to the nuclear lamina from lamin-B1 protein A-DamID (pA-DamID)^35^ experiments, mean radial positions from genomic loci positioning by sequencing (GPSeq)^36^ experiments, and distance distributions and single-cell chromosome tracing data from 3D FISH^18^ and DNA-MERFISH^6^ experiments, respectively. We define the nuclear microenvironment of a genomic region by an array of structural descriptors, including its nuclear radial position; association frequencies with and mean distances to nuclear speckles, the lamina, and nucleoli; the local chromatin fiber compaction; and local compartmentalization in form of the *trans* A/B ratio, defined as the fraction of its inter-chromosomal interactions with chromatin in the A (active) or B (inactive) compartment^6^ (Fig. 1a,b). These structural descriptors are determined in single-cell models, thereby revealing the cell-to-cell variability of the nuclear microenvironment for a genomic region across the population of models.

Our genome structure analysis provides several key findings. First, genomic regions with a strong preference for the same specific microenvironment across cells, thus having low structural cell-to-cell variability, are also most homogeneous in their functional properties. These chromatins are associated in most cells with either nuclear speckles or constitutive LADs and act as structural anchor points to genome organization. Second, our analysis shows that the subnuclear microenvironment of a genomic region reflects its transcriptional potential upon activation. Genes with high expression heterogeneity^37^ often show increased structural variability in the nucleus, indicating a contribution of extrinsic noise to gene expression heterogeneity^38^. Third, our observations confirm that Hi-C subcompartments^39^ define physically distinct chromatin environments, some of which (like A1) are linked to associations with nuclear speckles.

Although other computational approaches have modeled entire chromosomes, or even diploid genomes, from Hi-C data^18,34,40–56^, none so far has documented the predictive accuracy in reproducing multimodal experimental data, as presented here. Our findings demonstrate that our approach, from Hi-C data alone, produces predictive models that provide a detailed description of the subnuclear locations, folding, and compartmentalization of chromatin in diploid genomes. Therefore, our approach considerably expands the scope of Hi-C data analysis and is widely applicable to any cell type for which Hi-C data are available.

## Results

### Assessment of 3D genome structures

**Extended Data Fig. 1:**
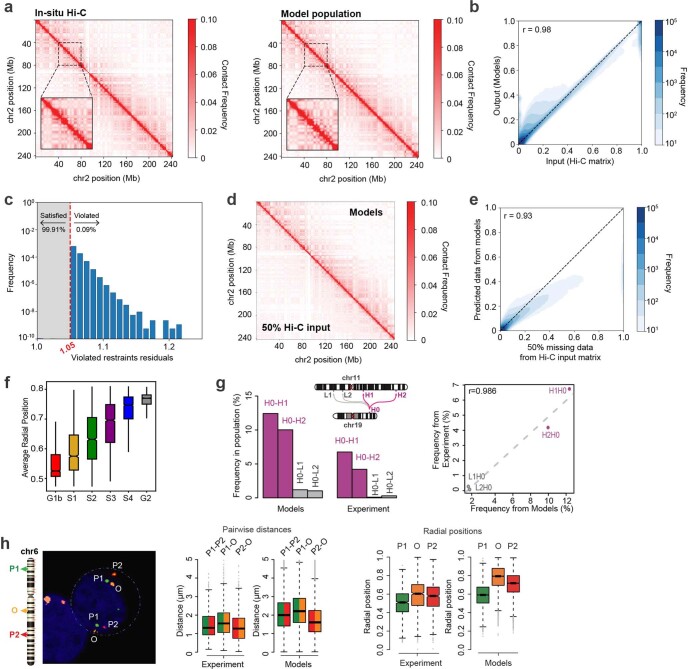
3D chromatin structure modeling and assessment. **a**, The contact probability matrix calculated from Hi-C (left) and the structure population (right) for chromosome 2. Zoomed-in heatmaps show the matrix for 40–80 Mb. **b**, Density scatter plot of contact probabilities from Hi-C data and structure population (Pearson correlation: *r* = 0.98, p = ~0). **c**, Histograms of restraint residuals from the structure population (Methods). A restraint residual less than 1.05 is considered satisfied and is not displayed in the histograms (99.9% of restraints fall in this category). **d**, The contact probability matrix for chromosome 2 showing the 50% randomly chosen dataset used as input (lower triangle) vs. the matrix generated from the structure population (upper triangle). **e**, Density plot of Hi-C contact probabilities missing in the input and their predictions from the structure population (Pearson correlation: *r* = 0.93, p = ~0). **f**, Average radial positions of chromatin in different replication phases^58^. Numbers of regions in each boxplot are (from left to right): 1,714, 1,921, 2,166, 2,246, 2,068, 2,064. **g**, Comparison of the inter-chromosomal loci co-localization frequencies between the observed occurrence in FISH experiments^18^ and in the structure population shown as bar plots (left), and scatter plot (right). **h**, A FISH image with three different probes at far-separating loci on chromosome 6 (left), the comparison of pair-wise distances of these loci in experiment and models (middle), and their relative radial positions in experiment and models (right). Numbers of regions in each boxplot for models: 10,000, for experiment: 892. In **f** and **h**, the box and the middle line in the box show the the interquartile range (IQR = Q3 – Q1) and the median. The vertical lines outside the box (and whiskers in h) extend to a maximum of 1.5*IQR beyond the box. Q1 and Q3 are the lower and upper quartile of the distribution. Outliers are shown as dots.

Here, we study 3D structures of diploid lymphoblastoid genomes (GM12878) from in situ Hi-C data^39^ at 200-kb (kilobase) resolution. Our method generates a population of 10,000 genome structures, in which all accumulated chromatin contacts are statistically consistent with contact probabilities from Hi-C experiments^33,34,32^. Structure optimization is achieved by solving a maximum likelihood estimation problem in an iterative fashion^33,34,48,32^ (Methods). The resulting genome structure population accurately reproduces experimental Hi-C contact probabilities (Pearson’s *r* = 0.98, genome-wide; 0.99 and 0.83 for *cis* and *trans* contacts, *P* = ~0, average chromosome SCC^57^ = 0.87; Extended Data Fig. 1a–c and Supplementary Information).

**Extended Data Fig. 2:**
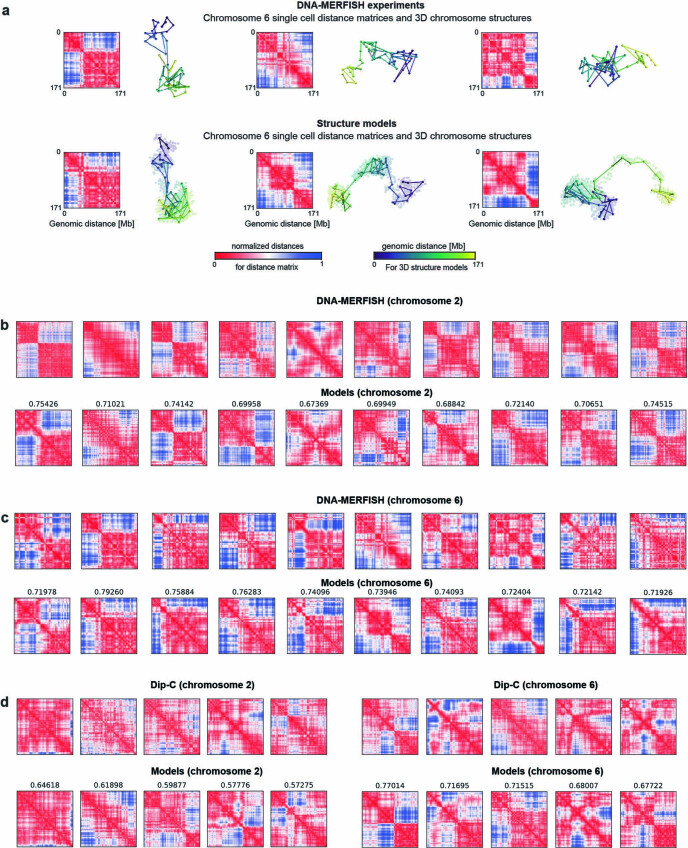
Assessment of single cell chromosome structures. **a**, Comparison of selected structures of chromosome 6 modeled in our simulations and imaged in DNA-MERFISH experiments^6^. 3D chromosome structures are shown to the right of the corresponding normalized distance matrix for structures from DNA MERFISH experiments (top row) and structure modeling (bottom row). Modeled structures at 200 kb base pair resolution have higher coverage than those from experiment. To allow direct comparison, we highlight those genomic regions that are imaged in DNA-MERFISH by opaque ball and stick representations, while other genomic regions are shown as translucent genomic regions. **b**, Comparison of distance matrices for single cell structures of chromosome 6 from DNA-MERFISH experiments (top) and structure models (bottom); models reproduce folding patterns of different chromosome conformations observed in experiment. **c**, Comparison of distance matrices for single cell structures of chromosome 2 from DNA-MERFISH experiments (top) and structure models (bottom). **d**, Comparison of chromosome structures between models from Dip-C experiment^25^ (top row) and our structure population (bottom row) for chromosome 2 (left) and chromosome 6 (right). Shown are the normalized distance matrices for chromosome structures in different conformational states. Both experimental and modeled structures show high degree of similarity. In **b**, **c**, and **d**, the numbers indicate the Pearson correlation between the experimental and the predicted distance matrices.

Our method is robust against missing data, as models generated from sparse Hi-C data (50% entries randomly removed) accurately predict the missing Hi-C contact frequencies (Pearson’s *r* = 0.93 (*cis*) and 0.69 (*trans*) of missing data, *P* = ~0; Extended Data Fig. 1d,e and Methods). Moreover, our models accurately predict, with significant correlation to their experimental values, a host of orthogonal data from lamin-B1 pA-DamID^35^, lamin-B1 TSA-seq^13^, SON TSA-seq^13^, and genomic loci positioning by sequencing (GPSeq)^36^ experiments (Pearson’s *r* = 0.80, 0.78, 0.87, and 0.80, respectively; Table 1 and Methods), which we will discuss in greater detail throughout this paper. Our models also confirm preferences for interior radial positions of chromatin replicated in the earliest G1b phase (*P* = 2.39 × 10^−77^, Mann–Whitney–Wilcoxon test, two-sided) and predict a gradual increase in average radial positions for chromatin replicated at later times^58^ (Extended Data Fig. 1f). Our results also agree with those of 3D FISH experiments^18^, namely co-location frequencies of four inter-chromosomal pairs of loci (Pearson’s *r* = 0.99, *P* = 0.014; Extended Data Fig. 1g) and distance distributions between three loci on chromosome 6 and relative differences in radial positions of these loci (Extended Data Fig. 1h). We also assessed our single-cell chromosome structures with data from multiplex DNA-MERFISH^6^ imaging and single cell Dip-C^25^ experiments, and found good agreement between the single-cell chromosome conformations in our models and those from DNA-MERFISH (Extended Data Fig. 2a–c) and Dip-C experiments (Extended Data Fig. 2d and Methods). All results were reproduced using technical replicates (Methods and Supplementary Information).

**Table 1:** Genome-wide Pearson and Spearman correlations between experimental and predicted omics and imaging data

	Pearson’s *r*	Spearman’s *r*
SON TSA-seq^13^	0.87	0.89
Lamin-B1 TSA-seq^13^	0.78	0.81
Lamin-B1 pA-DamID^35^	0.80	0.79
GPSeq^36^	0.80	0.79
SAF^6^	0.77	0.73
LAF^6^	0.64	0.58
NAF^6^	0.71	0.63
Median *trans* A/B ratio^6^	0.70	0.67

All *P* values are ~0. Chromosome X was discarded from genome-wide correlation calculations in TSA-seq, DamID, and GPSeq comparisons.

We now characterize the nuclear microenvironment of genomic regions by calculating a variety of structural descriptors for each genomic region in each single-cell model (Fig. 1a,b). Our aim is to identify characteristic nuclear microenvironments distinguishing chromatin of different functional states and to evaluate the roles of the nuclear topography and its cell-to-cell variability in regulating transcription and replication.

### Average nuclear position and its cell-to-cell heterogeneity

**Fig. 2:**
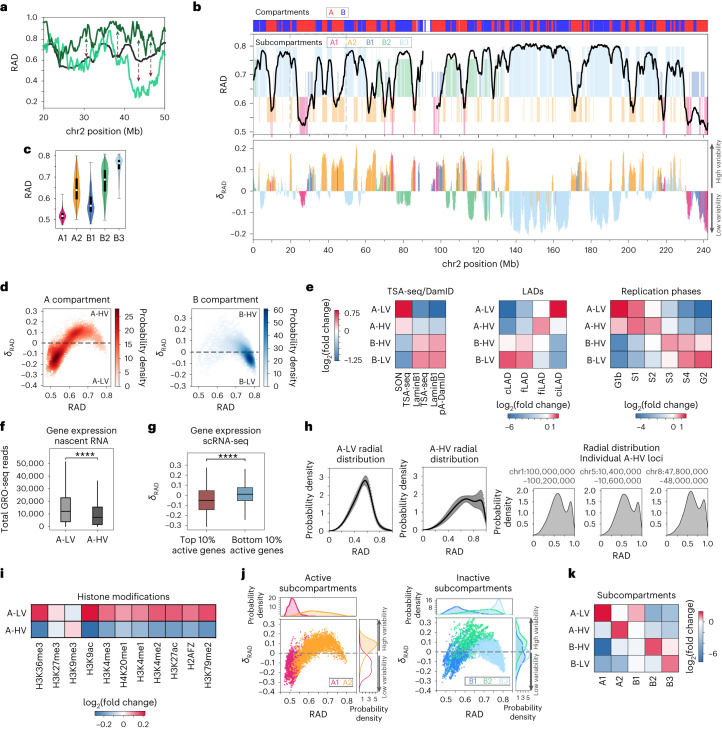
Radial chromatin positions and their cell-to-cell variability. **a**, RAD profiles for a 30-Mb region in chromosome 2. The black line shows the average radial position over the population of structures, and green lines show the radial positions in two different single structures. Arrows depict regions with high cell-to-cell variability. **b**, Chromatin RAD (top) and *δ*_RAD_ (bottom) in chromosome 2. Subcompartments are color-coded. **c**, RAD violin plots for each subcompartment. White circles and black bars show the median and the interquartile range (IQR: Q3–Q1). Whiskers show minima and maxima. Q1 and Q3 are the lower and upper quartile of the distribution. Numbers of regions used in each violin plot are: A1: 1,858, A2: 2,723, B1: 1,581, B2: 2,008, B3: 4,187. **d**, Scatter plots of *δ*_RAD_ versus RAD for chromatin in the A (left) and B (right) compartments. Dashed lines separate low (A-LV and B-LV) and high (A-HV and B-HV) levels of variability. **e**, Fold-change enrichment of TSA-seq^13^ and pA-DamID^35^ signals (left), LADs^61^ (middle), and replication phases^58^ (right) for A-LV, A-HV, B-LV, and B-HV. cLAD, constitutive LAD; fLAD, facultative LAD; ciLAD, constitutive inter-LAD; fiLAD, facultative inter-LAD. **f**, Box plots of the nascent RNA expression levels^63^ for A-LV and A-HV (Mann–Whitney–Wilcoxon test, two-sided). Numbers of regions used in each box plot are: A-LV: 3,164, A-HV: 2,731. **g**, *δ*_RAD_ box plots for chromatin with highest and lowest 10% transcript counts^37^ (Mann–Whitney–Wilcoxon test, two-sided). Numbers of regions used in each box plot are: Top 10%: 686, Bottom 10%: 684. In **f** and **g**, the box and the middle line in the box show the the interquartile range (IQR = Q3–Q1) and the median. The vertical lines outside the box extend to a maximum of 1.5 × IQR. **h**, Distributions for A-LV and A-HV RAD (left) and RAD for three representative A regions with high *δ*_RAD_ (right). The black lines in the left panels indicate the average distribution, and gray areas show the s.d. **i**, Fold-change enrichment of histone marks in A-LV and A-HV. **j**, Scatter plots of *δ*_RAD_ versus RAD for A (left) and B (right) chromatin. Top and right panels show RAD and *δ*_RAD_ distributions. **k**, Fold-change enrichment of subcompartments in A-LV, A-HV, B-LV, and B-HV.

**Extended Data Fig. 3:**
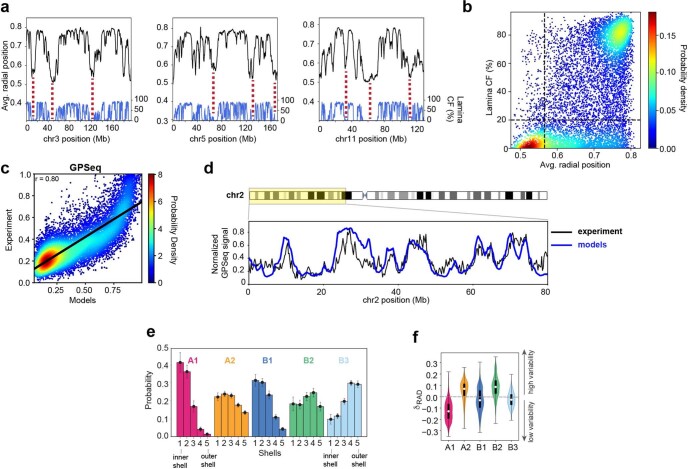
Assessment of radial positions. **a**, Average radial position profiles in chromosomes 3 (left), 5 (middle), and 11 (right). Also shown in blue are lamina contact frequencies (CF) from single cell lamin DamID experiments^61^. Valleys in the average radial position plots match well with low lamina CF regions (red dashed lines). **b**, Density scatter plot of average radial positions (RAD) of chromatin regions from the structure population against the lamina CF from single cell lamin DamID experiments in haploid KBM7 cell type^61^. 93% of chromatin regions with the 25% lowest average radial positions show either no detectable or only occasional contact with lamina (CF < 20%). Vertical and horizontal black dashed lines show the 25^th^ percentile average radial position and the 20% CF values, respectively. **c**, Scatter plot showing the comparison between experimental and predicted GPSeq scores^35^ (Pearson correlation: *r* = 0.80, p = ~0) **d**, Comparison of experimental and predicted GPSeq^35^ profiles for the 0–80 Mb region in chromosome 2. **e**, Probabilities for chromatin region of a given subcompartment to be located in any of the five concentric shells, each containing the same total amount of chromatin (Methods). Shell 1 is the most interior shell. Error bars show mean +/− s. d. Numbers of regions used in each bar for each shell for A1: 372, A2: 545, B1: 316, B2: 402, B3: 837. **f**, Violin plots for distributions of cell-to-cell variabilities of radial positions (δ_RAD_) for chromatin regions in different subcompartments. White circles and black bars show the median value and the interquartile range (IQR: Q3 – Q1). Whiskers show minima and maxima. Q1 and Q3 are the lower and upper quartile of the distribution. Numbers of regions used in each violin plot are: A1: 1,858, A2: 2,723, B1: 1,581, B2: 2,008, B3: 4,187. Dashed line separates low and high levels of variability.

The nuclear positions of genes are of functional relevance: FISH experiments revealed for some genes, upon transcriptional activation, a statistical shift of their locations towards the nuclear center^59,60^. Owing to the stochastic nature of genome structures, the radial nuclear position of a locus can vary between individual cells (Fig. 2a). However, the average radial position over all the models in the population reveals distinct preferences, which vary between different genomic loci (Fig. 2b, upper panel). The minima in the average radial profiles of chromosomes overlap with regions of lowest lamin-B1 DamID signals which have the lowest probabilities to interact with the nuclear envelope^61^ (Extended Data Fig. 3a,b). Our predictions also reproduce average radial locations, inferred from DNA digestion timing, detected in GPSeq experiments^36^ (Pearson’s *r* = 0.80, *P* = ~0; Extended Data Fig. 3c,d).

Notably, sequence positions that coincide with large transitions in the average radial profile often overlap with borders between the five primary Hi-C subcompartments identified by Rao et al.^39^ (Fig. 2b, top, and Methods) (that is, two transcriptionally active (A1, A2) and three inactive subcompartments (B1, B2, B3)). Chromatin in different subcompartments displays distinct distributions of average radial positions (Fig. 2c) and radial shell occupancy (Extended Data Fig. 3e), confirming previous observations^25,36^. For example, both A1 and B1 chromatins preferentially occupy the most interior radial shells of the nucleus, whereas B3 chromatin (mostly associated with LADs) shows a preferential location at the periphery and A2 chromatin shows a wide range of average locations without a marked radial preference (Fig. 2c and Extended Data Fig. 3e).

### Structural variability correlates with functional properties

We also calculated the cell-to-cell variability for gene locations (*δ*_RAD_), to quantify stochastic variations of radial positions between cells (Methods). *δ*_RAD_ differs distinctly between genomic loci (Fig. 2b, bottom). Sections of chromatin with high structural variability (*δ*_RAD_ > 0) alternate, in sharp transition, with regions of low variability (*δ*_RAD_ < 0)—transitions between high and low variability occur over relatively small sequence distances (Fig. 2b, bottom). These transitions align well with borders between subcompartments, most prominently between the A2 and B3 subcompartments (Fig. 2b, bottom). Continuous sections with similar *δ*_RAD_ values are often part of the same subcompartment.

We noticed that the structural variability of a genomic region is a strong indicator of its functional properties, for both the active A and inactive B compartment. Chromatin in the A compartment with low structural variability (*δ*_RAD_ < 0) (A-LV) (Fig. 2d) is enriched for high SON TSA-seq^13^ signals and low signals from lamin-B1 pA-DamID^35^ experiments; thus, A-LV regions have relatively short mean distances to nuclear speckles and are excluded from the nuclear periphery (Fig. 2e). Moreover, A-LV chromatin is highly enriched for constitutive inter-LADs^61^ (that is, regions never observed as LADs in any cell type) and is mostly replicated at the earliest G1b phase^58^. A-LV chromatin also shows significantly higher transcriptional activity than chromatin in the A compartment with high structural variability (*δ*_RAD_ > 0) (A-HV) (*P* = 1.35 × 10^−40^, Mann–Whitney–Wilcoxon test, two-sided; Fig. 2f). Overall, active genes with the highest number of transcripts in single-cell RNA-seq (scRNA-seq) experiments^37^ have a significantly lower *δ*_RAD_ compared to genes with the lowest number of transcripts (*P* = 3.45 × 10^−18^, Mann–Whitney–Wilcoxon test, two-sided; Fig. 2g).

By contrast, A-HV chromatin lacks SON TSA-seq signal enrichment and thus has larger mean distances to nuclear speckles, and is enriched for facultative inter-LADs (Fig. 2e). Notably, A-HV regions with the largest structural variability often show a bimodal distribution in their single-cell radial positions, an indication of two favored nuclear locations—at the nuclear interior and a peripheral location (Fig. 2h). We hypothesize that genes in these regions may exist in two functional states: active in the transcriptionally favorable interior, and silenced in the periphery. Indeed, compared with A-LV chromatin, A-HV chromatin is more enriched for the repressive trimethylated H3 K9 (H3K9me3) mark and depleted of the activating acetylated H3 K9 (H3K9ac) mark (Fig. 2i), which could point to a higher functional heterogeneity in single cells. Notably, the structural variability can distinguish A1 from A2 subcompartment chromatin (Fig. 2j, left, and Extended Data Fig. 3f)—93% of all A-HV regions in the active compartment are A2 chromatin, whereas A1 chromatin is strongly enriched in A-LV (Fig. 2j, left, and 2k).

Similar to the active compartment, B compartment chromatin also shows substantial differences in functional properties between the highly variable (B-HV) (*δ*_RAD_ > 0) and lowly variable (B-LV) (*δ*_RAD_ < 0) (Fig. 2d, right) genomic regions (Fig. 2e). Subsequently, the B1, B2, and B3 subcompartments are well distinguished by their structural variability and average radial positions (Fig. 2j, right), and B2 and B3 are enriched in B-HV and B-LV regions, respectively (Fig. 2k).

### Subcompartments separate into spatial partitions

**Fig. 3:**
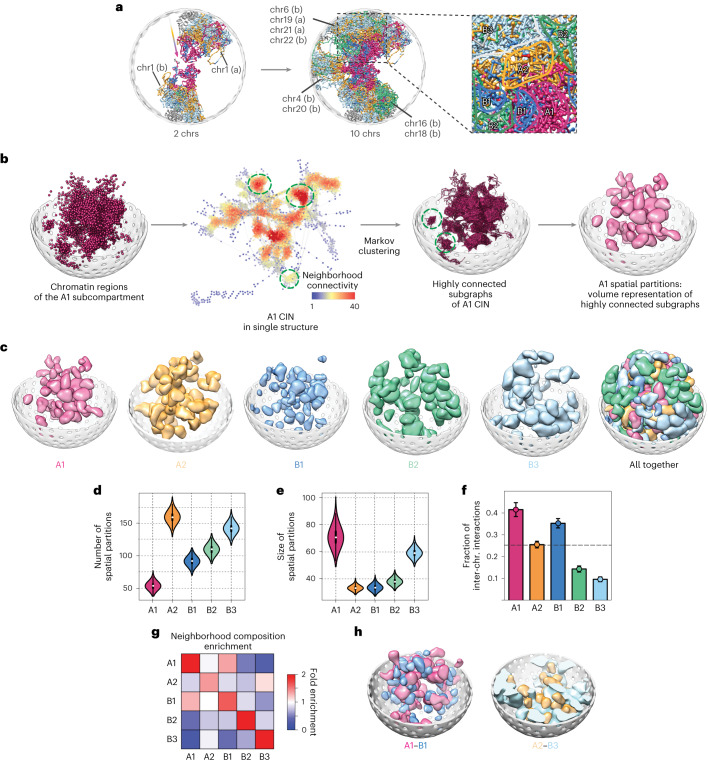
Spatial partitions of subcompartments. **a**, A representative genome structure showing chromosome folding patterns. Both images show the same structure with different numbers of chromosomes; copies are denoted with (a) and (b). The zoomed inset delineates regions that are primarily occupied by chromatin of the same subcompartment. Subcompartments in each chromatin region are color-coded (A1: pink, A2: yellow, B1: dark blue, B2: green, B3: light blue). **b**, Procedure to identify spatial partitions of subcompartments: A chromatin interaction network (CIN) is generated from all chromatin regions in a given subcompartment for each structure in the population. Each node in the CIN represents a single chromatin region connected by edges if the two regions are in physical contact in the 3D structure. Nodes are colored by their neighborhood connectivity, ranging from low (blue) to high (red). Highly connected subgraphs were then identified by Markov clustering of CINs (Methods) and visualized in the 3D structure (green dashed circles show examples). The rightmost image illustrates the volume occupied by a spatial partition in a single genome structure. **c**, Spatial partitions of subcompartments, shown by their occupied volume in the 3D structures. For clarity, only the 50 largest partitions are shown. **d**, Distributions of the number of partitions per genome structure. **e**, Distributions of the average size (that is, number of nodes) of subcompartment partitions. In **d** and **e**, white circles and black bars show the median value and the interquartile range (IQR: Q3–Q1). Tip points of the violins are minima and maxima. Q1 and Q3 are the lower and upper quartile of the distribution. **f**, Average fraction of inter-chromosomal edges in spatial partitions. Error bars indicate s.d., and the gray dashed line is the average fraction of all partitions. The number of data points (structures) used in each violin plot and statistics in **d**, **e**, and **f** is 10,000. **g**, Neighborhood enrichment of chromatin in each subcompartment (Methods). **h**, A representative structure showing examples of colocalizations of A1–B1 and A2–B3 partitions in the 3D space.

Chromosome folding permits functionally related chromatin, separated in sequence, to assemble into spatial compartments (Fig. 3a). The single-cell interaction networks (CINs) of chromatin in the same subcompartment show a heterogeneous network organization with clusters of highly connected and physically separated subgraphs (that is, local partitions) reminiscent of microphase fragmentation^62^ (Fig. 3b and Methods). These spatial partitions can be visualized in single genome structures by the occupied volume of the contained chromatin (Fig. 3b,c).

Network structures differ between individual subcompartments. While A1 chromatin is fragmented into the smallest number of partitions with the largest sizes (Fig. 3d,e and Extended Data Table 1) and highest fraction of inter-chromosomal interactions (Fig. 3f), A2 chromatin is fragmented into substantially larger numbers of smaller partitions, dominated by intra-chromosomal interactions (Extended Data Table 1 and Fig. 3d–f). Among the B compartment, B3 chromatin has the largest partitions, dominated by intra-chromosomal interactions (Fig. 3e,f).

The larger partition sizes of A1 and B3 chromatin lead to a more homogenous compartmentalization, with each having a higher neighborhood enrichment score with its own kind (see high enrichment fold along the diagonal in Fig. 3g and Methods). Smaller partition sizes of A2 and B1 chromatin lead to relatively high neighborhood enrichment with other chromatin (see off diagonal enrichment in Fig. 3g). A2 partitions are often associated with B3 chromatin, whereas B1 partitions are associated with A1 chromatin^36^ (Fig. 3g,h).

**Fig. 4:**
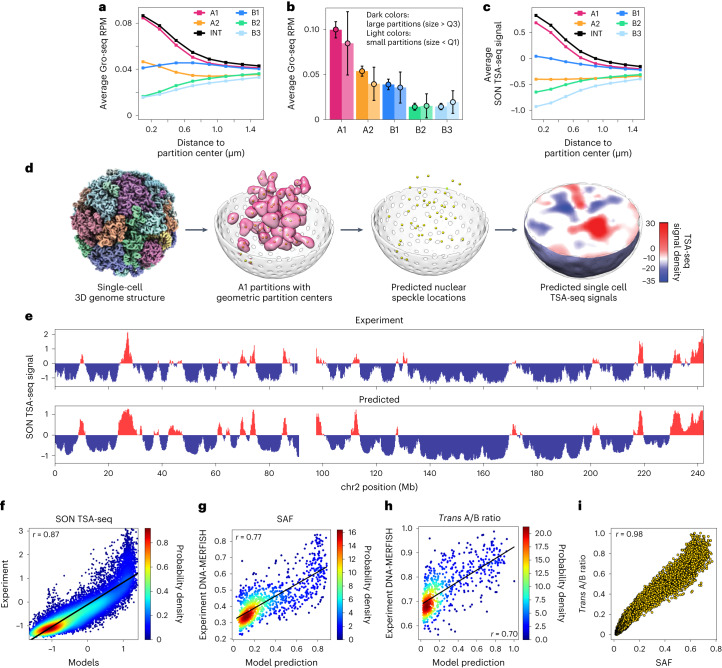
SON TSA-seq predictions using 3D structures. **a**, Average GRO-seq signal^63^ (RPM) of chromatin regions with respect to their 3D distances to partition centers in different Hi-C subcompartments or interior (INT) chromatin (Methods). **b**, Comparison of average GRO-seq signals^63^ for chromatin in large (size > Q3, dark colors) and small (size < Q1, light colors) spatial partitions for different subcompartments. Error bars show mean ± s.d. Total numbers of data points (all partitions in all structures) used in statistics are: A1 large: 257,454, A1 small: 245,698, A2 large: 354,618, A2 small: 689,536, B1 large: 212,557, B1 small: 629,112, B2 large: 306,571, B2 small: 364,678, B3 large: 778,382, B3 small: 136,143. **c**, Average SON TSA-seq signals^13^ of chromatin regions with respect to their 3D distances to partition centers in Hi-C subcompartments and interior (INT) chromatin (Methods). **d**, Procedure for SON TSA-seq signal prediction from 3D models: The geometric centers of identified A1 partitions in each single structure are used as point sources for the simulation of SON-TSA-produced tyramide free-radical diffusion^13^. SON TSA-seq signals are averaged over all structures (Methods). The rightmost image shows a cross-section of the predicted TSA-seq signal density distribution in a genome structure. **e**, Comparison of the experimental and predicted SON TSA-seq profiles for chromosome 2 (Pearson correlation: *r* = 0.90, *P* = ~0). **f**, Scatter density plot of the experimental versus predicted SON TSA-seq signals genome-wide (Pearson correlation: *r* = 0.87, *P* = ~0). **g**, Scatter density plot of the predicted speckle association frequency (SAF) versus SAF determined in DNA-MERFISH experiments^6^ for 1,041 imaged loci (Pearson correlation: *r* = 0.77, *P* = ~0). **h**, Scatter density plot of the median *trans* A/B ratios predicted in our models (Methods) versus those from the DNA-MERFISH experiment^6^ for 724 imaged loci that share the same compartment in GM12878 and IMR-90 cells (Pearson correlation: *r* = 0.70, *P* = ~0). **i**, Scatter plot of the predicted median *trans* A/B ratios versus SAF for each chromatin region in our models (Pearson correlation: *r* = 0.98, *P* = ~0).

When we mapped nascent RNA expression from GRO-seq experiments^63^ onto our genome structures, we found increasing transcriptional activities towards the centers of A1 partitions (Fig. 4a). A2 partitions show similar trends, although substantially lower signals (Fig. 4a). We also observe that highly expressed genes reside preferably in larger partitions, and expression levels at the centers of large A1 and A2 partitions are notably higher than those of smaller ones (Fig. 4b). These observations indicate that spatial partitions of active chromatin are regional territories of highest transcriptional activities.

### Predicting locations of nuclear speckles

**Extended Data Fig. 4:**
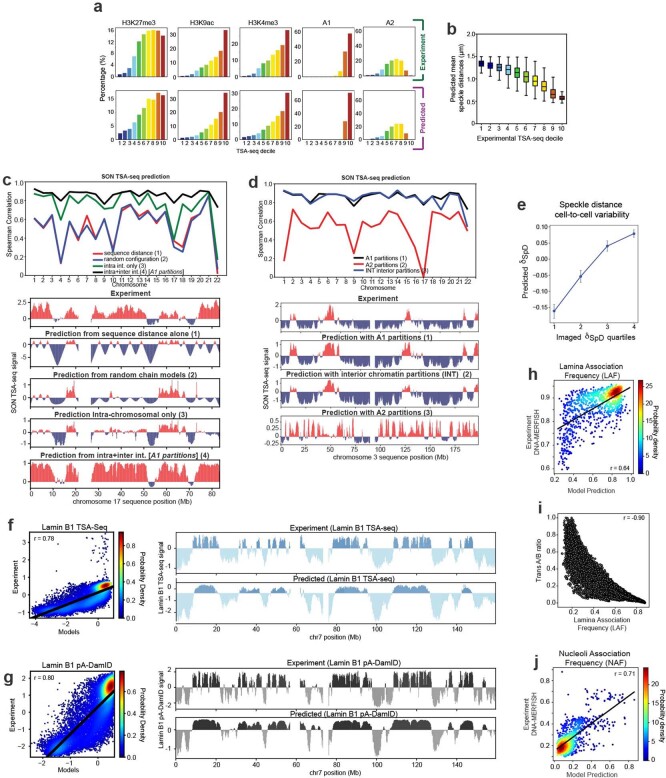
Predictions of nuclear body related experimental data using 3D models. **a**, Fraction of mapped histone mark peaks and number of A1/A2 chromatin regions in experimental^13^ (top) and predicted SON TSA-seq deciles (bottom). **b**, Predicted mean speckle distances (SpD) for chromatin in experimental SON TSA-seq deciles^13^. The box and the middle line show the interquartile range (IQR = Q3–Q1) and the median. The vertical lines and whiskers extend to a maximum of 1.5*IQR. Q1 and Q3 are the lower and upper quartile of the distribution. Number of regions used in each decile boxplot is 1,368. **c**, Spearman correlations between the experimental^13^ and predicted SON TSA-seq signals (top) using sequence distances to A1 clusters (red), 3D distances to A1 partitions in random territories (blue), 3D distances to A1 regions in the same chromosome (green), and 3D distances to A1 partitions (black), and chromosome 17 profiles (Spearman correlations: 0.37, 0.30, 0.38, 0.78, respectively, bottom). **d**, Spearman correlations between experimental^13^ and predicted SON TSA-seq signals (top) using A1 (black) or A2 (red) partitions, or partitions from chromatin with 10% lowest average radial positions (INT, blue), and chromosome 3 profiles (Spearman correlations: 0.88, 0.89, 0.58, respectively, bottom). **e**, Cell-to-cell variability of speckle distances (δ_SpD_) from DNA-MERFISH^6^ and models. Error bars show mean +/− s.d. of predicted δ_SpD_ values in each imaged δ_SpD_ quartile. Number of regions used in each quartile (left to right): 245, 241, 247, 246. Scatter plots of the experimental and predicted signals (left) and chromosome 7 profiles (right) for (**f**) LaminB1 TSA-seq^13^ (Pearson correlation: *r* = 0.78, p = ~0) and (**g**) LaminB1 pA-DamID^34^ (Pearson correlation: *r* = 0.80, p = ~0). **h**, Scatter plot of lamina association frequencies (LAF) from DNA-MERFISH^6^ and models (Pearson correlation: *r* = 0.64, p = ~0). **i**, Scatter plot of median *trans* A/B ratios and LAF for each region predicted from models (Pearson correlation: *r* = −0.90, p = ~0). **j**, Comparison of nucleoli association frequencies (NAF) from DNA-MERFISH^6^ and models (Pearson correlation: *r* = 0.71, p = ~0).

Mapping TSA-seq data^13^ onto our genome structures revealed the strongest TSA-seq signals—and thus the smallest mean speckle distances—for chromatin located towards the central regions of A1 partitions (Fig. 4c); this suggests that the center locations of A1 partitions could represent positions of nuclear speckles in individual cell models. To test this assumption, we simulated the experimental TSA-seq process by using A1 partition centers as approximate speckle locations (Fig. 4d and Methods). The simulated, population-averaged SON TSA-seq data from our models show highly significant correlation with the experimental SON TSA-seq values^13^ (Pearson’s *r* = 0.87, *P* = ~0), capturing well both peak sizes and signal distributions (Fig. 4e,f). For instance, the TSA-seq profile of chromosome 2 is reproduced with high correlation (Pearson’s *r* = 0. 90, *P* = ~0) across the entire chromosome profile, despite containing few A1 regions (6.4%) (Fig. 4e). Chromatins grouped by predicted TSA-seq signals show characteristic enrichment of histone modifications, identical to those observed in the experiment^13^ (Extended Data Fig. 4a). Moreover, predicted speckle locations confirm the proposed correlation between mean speckle distances of chromatin and its experimental TSA-seq signal (Extended Data Fig. 4b).

We then found out that speckle locations can be predicted accurately even without relying on A1 subcompartment annotations, which are only available for a limited number of cell lines. We found that spatial partitions of chromatin with lowest average radial positions in the bottom 10% (labeled as internal (INT) in Fig. 4c) predict speckle locations within 500 nm to those derived from A1 partitions in 99% of structures (78% of chromatin with 10% lowest average radial positions are part of A1). Subsequently, the SON TSA-seq data can also be predicted from INT centers with almost identical accuracy (Pearson’s *r* = 0.86, *P* = ~0) (Extended Data Fig. 4d and Extended Data Table 2). Further investigations showed that only INT or A1 chromatin partition centers predict accurately the nuclear speckle locations in our models (Extended Data Fig. 4c,d and Extended Data Table 2).

### Predicting speckle-associated structural features

With predicted speckle locations as reference points, we can now calculate speckle-associated features (SpD, SAF, *δ*_SpD_, S-TSA in Fig. 1b) for each genomic region (Methods). The predicted speckle association frequencies (SAFs) of genomic regions agree with a recent DNA-MERFISH microscopy study^6^ with high correlation (Pearson’s *r* = 0.77, *P* = 1.2 × 10^−202^; Fig. 4g and Methods). Predicted *trans* A/B ratios also show a high correlation with those from DNA-MERFISH^6^ (Pearson’s *r* = 0.70, *P* = 7.6 × 10^−109^; Fig. 4h). We also found a moderate but highly significant correlation for the cell-to-cell variability of speckle distances (*δ*_SpD_) between our models and the experiment (Extended Data Fig. 4e; Pearson’s *r* = 0.352, *P* = 7 × 10^−30^). Interestingly, we find a strong anticorrelation between the inter-chromosomal contact probability (ICP) of a genomic region and its mean speckle distance (SpD) (Pearson’s *r* = −0.95, *P* = ~0; Supplementary Fig. 1). Thus, the surroundings of speckles are strongly enriched in interchromosomal interactions, in particular for A compartment chromatin. This observation is confirmed by a strong correlation between a gene’s SAF and *trans* A/B ratio^6^ (Pearson’s *r* = 0.98, *P* = ~0; Fig. 4i).

### Defining lamina- and nucleoli-associated features

Our models also accurately predict structural features describing chromatin positioning relative to the nuclear lamina (LAF, L-TSA in Fig. 1b). For instance, our models predict experimental lamin-B1 TSA-seq data with high correlation^13^, thus revealing accurate mean distances of genomic regions to the nuclear envelope (Pearson’s *r* = 0.78, *P* = ~0; Extended Data Fig. 4f and Table 1). Our models also predict lamin-B1 pA-DamID^35^ data with high correlation (Pearson’s *r* = 0.80, *P* = ~0; Extended Data Fig. 4g and Table 1), and thus could predict well the contact frequencies of genomic regions with the nuclear periphery. Finally, our models also reproduce experimental lamina association frequencies (LAFs)^6^ (Pearson’s *r* = 0.64, *P* = ~3.6 × 10^−119^; Extended Data Fig. 4h), despite the differences in shape between IMR-90 and GM12878 cell nuclei. Predicted LAF values are inversely correlated with a gene’s *trans* A/B ratios, confirming previous observations from DNA-MERFISH imaging^6^ (Extended Data Fig. 4i).

Moreover, our models also predict nucleolus-associated structural features (NuD, *δ*_NuD_, NAF (nucleoli association frequencies), N-TSA in Fig. 1; Extended Data Fig. 4j and Methods).

**Extended Data Fig. 5:**
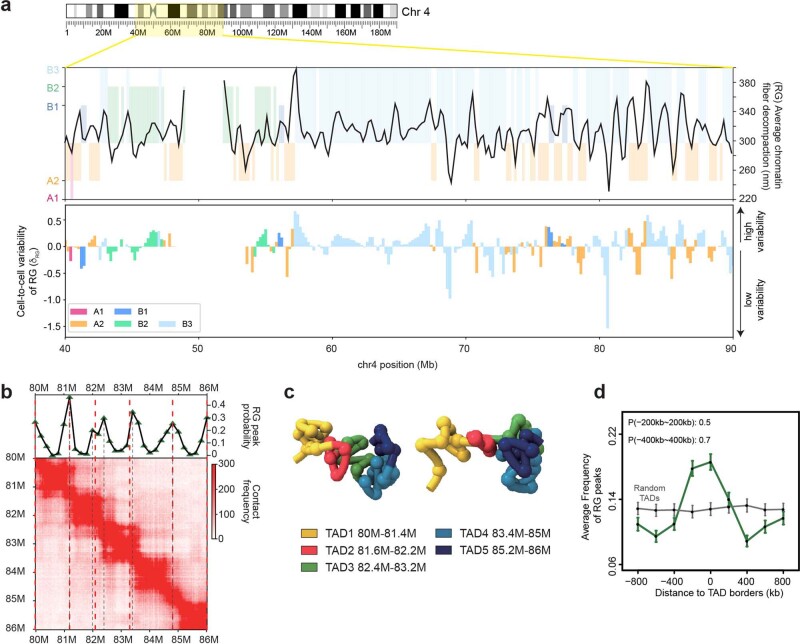
Chromatin compaction and TAD borders. **a**, Average radius of gyration (RG, that is local decompaction) profile for chromatin in the 40–90 Mb region of chromosome 4. The background is color coded by the subcompartment annotations of chromatin (top). Cell-to-cell variability of RG values (δ_RG_) in the structure population for the same chromatin regions. Negative values indicate regions with low RG variability (bottom). Bars are color coded by the subcompartment annotations of the corresponding chromatin regions. **b**, RG peak frequencies (that is, the fraction of models showing a RG maximum at a given position) for a 6-Mb region in chromosome 4 (80–86 Mb) (top), and Hi-C contact frequency heatmap for the same region showing TAD borders identified by TopDom^79^ (bottom). Regions with RG peak frequency maxima are shown with gray dashed lines, and either overlap or are very close to TAD borders identified by TopDom (red dashed lines). **c**, Two representative structures showing chromatin folding patterns for the same chromatin region in b. TAD identities are shown by color code. **d**, Averaged RG peak frequencies for loci at TopDom TAD borders (green) compared to randomly selected loci (gray). In around 50% of structures, there is a RG peak in the immediate neighboring region of a TAD border (±200 kb, Mann-Whitney-Wilcoxon test, two-sided, p = 1.47×10^−200^ compared to random) In ~70% of structures there is a RG peak within a ±400 kb range of a TAD border (Mann-Whitney-Wilcoxon test, two-sided, p = 2.46 × 10^−7^ compared to random). Data are presented as mean values +/− s.d. Numbers of data points at each distance used for TopDom borders (green): 1,839, for random borders (gray): 2,491.

Finally, we also calculate structural features of the chromatin fiber (Extended Data Fig. 5a and Methods), including local chromatin compaction (RG), which confirm the locations of TAD borders (Extended Data Fig. 5b–d).

### The role of the nuclear microenvironment in gene function

Overall, we calculate a total of 17 structural features from our single-cell genome structure models (Fig. 1 and Supplementary Figs. 2–22). Collectively, these features define the nuclear microenvironment of each genomic region, which allows us to assess the role of the nuclear microenvironment in explaining functional differences between chromatin, in particular for gene transcription, DNA replication, and chromatin compartmentalization.

### Gene transcription

**Fig. 5:**
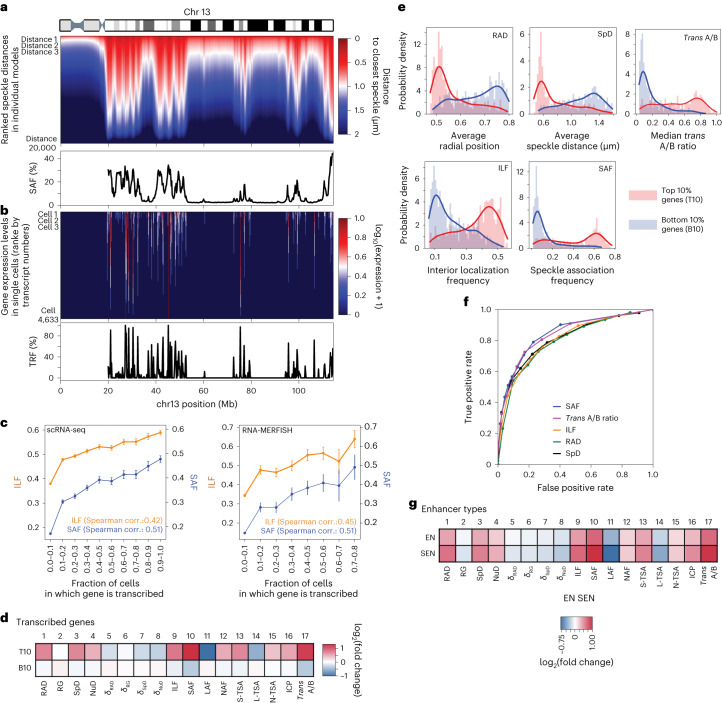
Relationship between 3D chromatin structure and transcriptional activity. **a**, Top, Heatmap of gene–speckle distances in chromosome 13 in 10,000 structures. Each column shows, for a given gene, the gene–speckle distances in all 10,000 structures of the population. In each column, gene–speckle distances are sorted in ascending order from top to bottom, with short distances (dark red) to large distances (dark blue). Bottom, SAF for each chromatin region in chromosome 13. **b**, Top, Heatmap of single-cell mRNA counts of genes in chromosome 13 in all 4,633 G1 cells measured by scRNA-seq experiment^37^. For a given gene, each column shows the observed mRNA transcript count in each cell of the population of cells. In each column, mRNA transcript counts are sorted in descending order from top to bottom, with high counts (dark red) to zero counts (dark blue). Bottom, TRF for each gene in chromosome 13 from scRNA-seq data^37^ (Methods). **c**, ILF and SAF values for genes with different TRF ranges from scRNA-seq^37^ (left) and nascent RNA-MERFISH imaging^6^ (right). Error bars show mean ± s.d. of ILF and SAF values in each TRF range. The numbers of regions used in each range in the left plot are (from left to right): 4,940, 960, 669, 478, 352, 280, 220, 204, 167, 301. The numbers of regions used in each range in the right plot are (from left to right): 692, 75, 65, 59, 41, 27, 10, 10. **d**, Fold-change enrichment of the 17 structural features for chromatin with top 10% highest (T10) and bottom 10% lowest (B10) transcript numbers of actively transcribed genes according to scRNA-seq data^37^ (Methods). **e**, Distributions of several structural features for T10 and B10 regions. **f**, ROC curves for RAD, SpD, ILF, SAF, and *trans* A/B ratios to distinguish T10 and B10 regions (area under the curve values are 0.65, 0.72, 0.81, 0.85, 0.84, respectively). **g**, Fold-change enrichment of the 17 structural features for enhancer (EN) and superenhancer (SEN) chromatin regions.

First, we compare the stochastic variability of gene–speckle distances (*δ*_SpD_) in single-cell models with the heterogeneity of single-cell gene expression from single-cell RNA sequencing (scRNA-seq) experiments^37^. Cumulatively ranked single-cell distances of a genomic region to its nearest predicted speckle (Fig. 5a, top) show striking similarities to the cumulatively ranked number of gene transcripts of the corresponding genes in a cell population from scRNA-seq^37^ (Fig. 5b, top, and Methods). Subsequently, the gene transcription frequency (TRF), defined as the fraction of cells a gene transcript is detected in scRNA-seq^37^ (Fig. 5a,b, bottom), shows a highly significant correlation with the SAF predicted from the models (Fig. 5c, left panel, Spearman’s *r* = 0.51, *P* = ~0). Thus, genes with transcripts in a large fraction of cells are also located close to speckles in a large fraction of models. We also validated these findings with transcription frequencies measured from RNA-MERFISH microscopy for 1,137 genes^6^. Here as well, we observe the identical highly significant correlation between TRF and SAF (Spearman’s *r* = 0.51, *P* = 1.6 × 10^−64^) (Fig. 5c, right panel). Interestingly, a gene’s interior location frequency (ILF; Methods) shows substantially smaller correlation with the TRF than with the SAF, for both scRNA-seq^37^ and RNA-MERFISH^6^ data (Spearman’s *r* = 0.42, *P* = ~0 (scRNA-seq) and *r* = 0.45, *P* = 4.1 × 10^−50^ (RNA-MERFISH)) (Fig. 5c). These observations indicate a possible role for single-cell variations of a gene’s nuclear microenvironment in its expression heterogeneity.

Moreover, we found that genes in the top 10% of genes with the highest numbers of transcripts (T10) are distinguished in their nuclear microenvironment from genes in the bottom 10% (B10). T10 genes show strong enrichment for several structural features (Fig. 5d, for example SAF and *trans* A/B**)**, while being depleted in *δ*_RAD_, *δ*_SpD_, and *δ*_NuD_. Thus, T10 genes show a strong preference for the same specific microenvironment in different cells, while B10 genes do not—their microenvironment is highly variable between cells without clear association preferences to nuclear bodies.

The distribution of feature values for the most discriminative features (SpD, ILF, SAF, RAD, and *trans* A/B) are quite different between the T10 and B10 genes (Fig. 5e). However, SAF and the highly correlated *trans* A/B ratio outperform all other features, including the radial gene position (RAD), in distinguishing T10 from B10 genes, as shown by the receiver operating characteristic (ROC) curves (Fig. 5f) (AUC for SAF = 0.85, RAD = 0.65). This finding could indicate that the general preference of highly expressed genes at interior radial positions may be an indirect consequence of favored associations with nuclear speckles, which themselves show stochastic preferences towards the nuclear interior^13,64^.

Moreover, genes controlled by superenhancers (SEN) show overall higher fold enrichments in structural features than genes controlled by regular enhancers (EN) (Methods). Thus, SEN genes reveal stronger preferences in their nuclear microenvironment between cells, particularly for higher SAF, interior positions, *trans* A/B, ICP, and depletion of LAF values (Fig. 5g).

### The organizing role of nuclear speckles and lamina

**Fig. 6:**
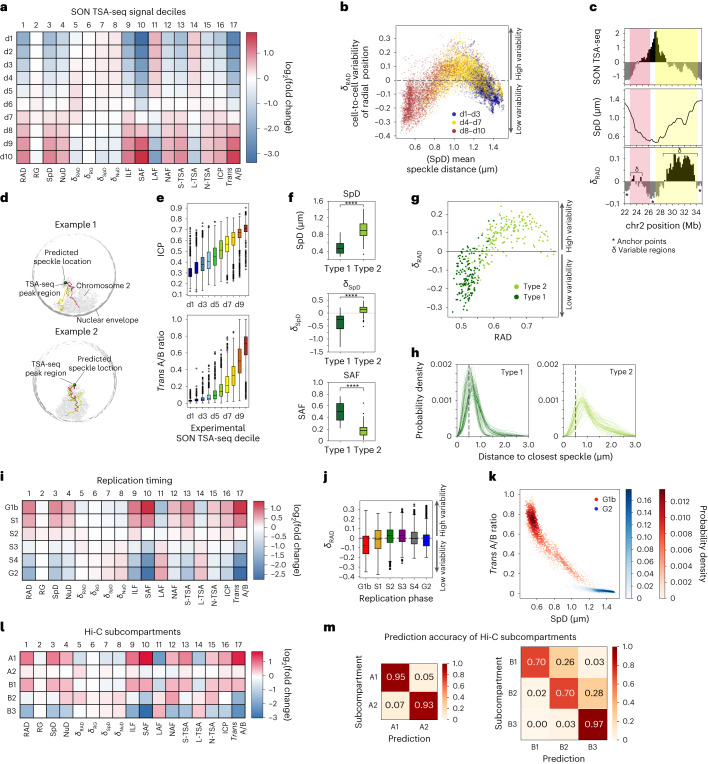
Structural features of microenvironments. **a**, Fold-change enrichment of the 17 structural features for chromatin in SON TSA-seq deciles^13^. **b**, *δ*_RAD_ versus SpD for chromatin in each decile (d1–d3: blue, d4–d7: yellow, d8–d10: red). **c**, SON TSA-seq (top), SpD (middle) and δ_RAD_ (bottom) profiles for a ~11-Mb region of chromosome 2. Stars and *δ* in the lower panel indicate anchor regions with low and high *δ*_RAD_. **d**, Two representative structures showing folding patterns of chromatin for the same region as in **c**, together with the nuclear envelope, the closest predicted speckle location (green), and the rest of chromosome 2 (gray). In **c** and **d**, valley-to-peak: red, peak-to-valley: yellow. **e**, Distributions of ICP (top) and *trans* A/B ratios (bottom) for chromatin in each decile. The number of regions used in each decile boxplot is 1,368. **f**, Distributions of SpD (top), δ_SpD_ (middle), and SAF (bottom) for regions where type I and type II TSA-seq peaks^13^ are located (Mann–Whitney–Wilcoxon test, two-sided). The numbers of regions used in each boxplot are: Type I: 165, Type II: 191. **g**, *δ*_RAD_ versus RAD for type I (dark green) and type II (light green) chromatin. **h**, Distributions of gene–speckle distances for randomly selected 50 type I (left) and type II loci (right). The gray dashed line indicates the 0.5 μm distance. **i**, Same as **a**, but for different replication phases^58^. **j**, Distributions of *δ*_RAD_ for chromatin in each replication phase^58^. Numbers of regions used in each boxplot are (from left to right): 1,714, 1,921, 2,166, 2,246, 2,068, 2,064. **k**, *Trans* A/B ratios versus SpD for chromatin in G1b (red) and G2 (blue)^58^. **l**, Same as **a** and **i**, but for each subcompartment. **m**, Confusion matrices for the prediction of A (left) and B (right) subcompartments (Methods). In **f** and **j**, the box and the middle line in the box show the the interquartile range (IQR = Q3–Q1) and the median. The vertical lines outside the box (and whiskers in **f** and **j**) extend to a maximum of 1.5 × IQR. Q1 and Q3 are the lower and upper quartile of the distribution. Outliers are shown as circles.

**Extended Data Fig. 6:**
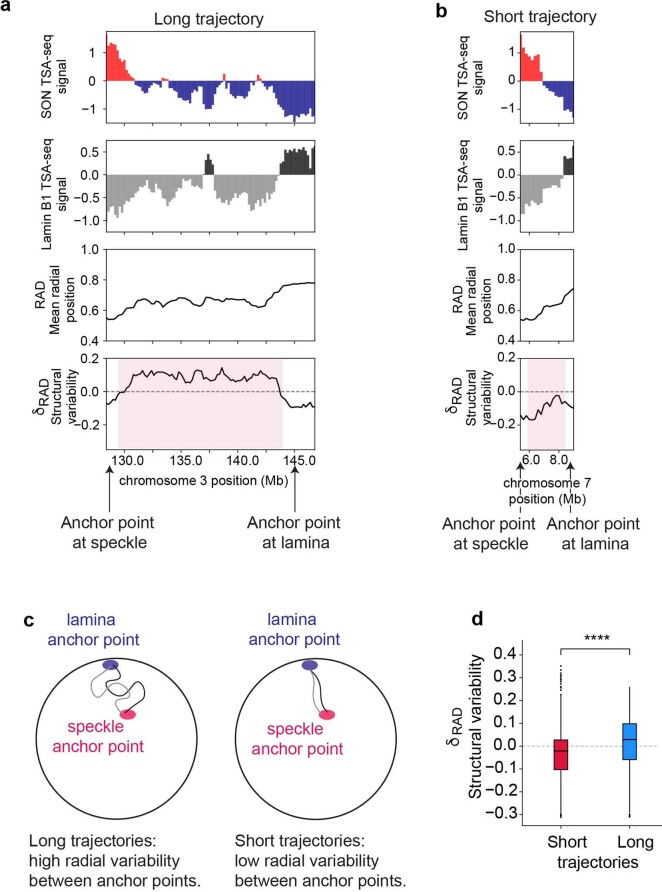
Chromatin trajectories with speckle and lamina anchor points. **a**. Structural feature profiles for a representative example of a long trajectory (chromosome 3 128.4–146.8 Mb). Calculated feature profiles include the experimental SON TSA-seq data, Lamin B1 TSA-seq data, predicted mean radial positions (RAD), and predicted structural variability (δ_RAD_). **b**. The same structural feature profiles for a representative example of a short trajectory (chromosome 7 5.4–9.2 Mb). Anchor points (indicated by arrows in both **a** and **b**) are defined by extreme average radial positions (interior for speckle anchor and exterior position for lamina anchor points) as well as high SON TSA-seq and lamin B1 TSA seq values. **c**. Schematic illustration of short and long chromatin trajectories with speckle and lamina anchor points and two hypothetical chromatin conformations connecting the anchor points. Chromatin trajectories are defined as consecutive chromatin sequence regions between a speckle and nearest lamina anchor points. **d**. Box plots of radial structural variability (δ_RAD_) distributions for chromatin regions in short and long trajectories (Mann-Whitney-Wilcoxon test, two-sided, p-value = 1.48 × 10–18). The box and the middle line in the box show the the interquartile range (IQR = Q3 – Q1) and the median. The vertical lines outside the box extend to a maximum of 1.5*IQR beyond the box. Q1 and Q3 are the lower and upper quartile of the distribution. Outliers are shown as dots. Numbers of regions used in each boxplot for short: 321, for long: 1,929.

Our approach allows a detailed analysis of chromatin speckle interactions. Chromatins divided into ten groups on the basis of their experimental SON TSA-seq signals^13^ show distinct structural enrichment patterns, which gradually change with increasing SON TSA-seq values (Fig. 6a). Chromatins in deciles d4–d7 (intermediate mean speckle distances) are highly variable in their nuclear positions (*δ*_RAD_) and show no preferred associations with nuclear bodies studied here (Fig. 6a,b). By contrast, chromatins in the first (d1, d2) and last (d9, d10) deciles show the highest fold enrichments and thus the most stable microenvironment with strong structural homogeneity between cells in the population; these regions show the lowest *δ*_RAD_ and have the smallest and largest SpD, respectively (Fig. 6b). The latter coincides with mostly B-LV chromatin located at the nuclear periphery (Fig. 2d, right panel) and subsequently high lamin-B1 TSA-seq signal enrichment (Fig. 2e, left panel). Thus, these genomic regions provide stable structural anchor points at the nuclear periphery. Speckle-associated chromatin with the highest SON TSA-seq signals (d8–d10 in Fig. 6b) and SAF values also show relatively low cell-to-cell structural variability in their radial positions (*δ*_RAD_) (Fig. 6b, mostly A-LV in Fig. 2d, left panel). Since speckle locations are mostly excluded from the nuclear periphery^13,64^, these regions act as stable anchor points at the nuclear interior (mostly A-LV in Fig. 2d, left panel). Therefore, both the lamina compartment and nuclear speckles act as anchor points for scaffolding the organization of the spatial genome. These observations provide a structural interpretation of the steep transitions between low and high signal peaks in SON TSA-seq profiles, previously reported as TSA-seq trajectories^13^ (Fig. 6c and Extended Data Fig. 6a). These transitions correspond to the sequence stretches between two consecutive anchor points, each with relatively low *δ*_RAD_, and coincide with steep transitions in average speckle distances and radial positions (Fig. 6c and Extended Data Fig. 6a–c). In a fraction of models, these chromosome regions fold from anchor regions at the outer nuclear periphery towards anchor points at the nuclear interior, where the SON TSA-seq peak region is often associated with a nuclear speckle and forms the apex of a chromosomal loop, which then traces back to the nuclear periphery (Fig. 6d and Extended Data Fig. 6c). We found that *δ*_RAD_ in long trajectories (median length of 19.1 Mb between two consecutive anchor points) is significantly larger than for chromatin regions in short trajectories (median length 4.8 Mb) (Mann–Whitney two-sided test, *P* = 1.48 × 10^−18^; Extended Data Fig. 6d). Therefore, sequence locations of consecutive anchor points can modulate the structural properties for chromatin between anchor points over an extended genomic range, and disruption of an anchor point would likely affect structural properties of genomic regions over an extended sequence distance.

We also observe that SON TSA-seq signals (that is, mean speckle distances) positively correlate with both the ICP (Methods) (Pearson’s *r* = 0.76 *P* = ~0, Fig. 6e, top) and *trans* A/B ratio (Fig. 6e, bottom). These observations imply that surroundings of nuclear speckles act as major hubs for inter-chromosomal interactions of transcriptionally active genomic regions, confirming similar findings reported earlier^13,23^.

Finally, our models reveal distinct structural differences for genomic regions with high and intermediate SON TSA-seq signal peaks (that is, previously labeled type I and type II transcription ‘hot zones’^13^) (Fig. 6f). The vast majority of type II peaks show significantly higher speckle distance and radial variability (*δ*_SpD_, *δ*_RAD_) (Fig. 6f,g) than type I peaks and thus do not reside stably at intermediate speckle distances. Instead, they show a wider, in many cases bimodal, speckle distance distribution in comparison to type I peaks (Fig. 6h).

### The role of microenvironment in replication timing

Variations in the replication timing^58^ of chromatin are mirrored by distinct differences in their nuclear microenvironment (Fig. 6i). For example, chromatin that replicates at early time points (G1b, S1) is most enriched for high SAF and *trans* A/B ratio, as well as low structural variability (Fig. 6i,j), whereas late-replicating chromatin (S4 and G2 phase) are depleted of interior locations and SAF, and strongly enriched for lamina-associated features (Fig. 6i). Overall, SAF, SpD, and *trans* A/B ratio are more discriminative (that is, these features have higher fold changes) than features related to radial positions (RAD, ILF) in distinguishing early-replicating (G1b) from late-replicating chromatin (G2) (Fig. 6k).

### Chromatin compartmentalization

**Extended Data Fig. 7:**
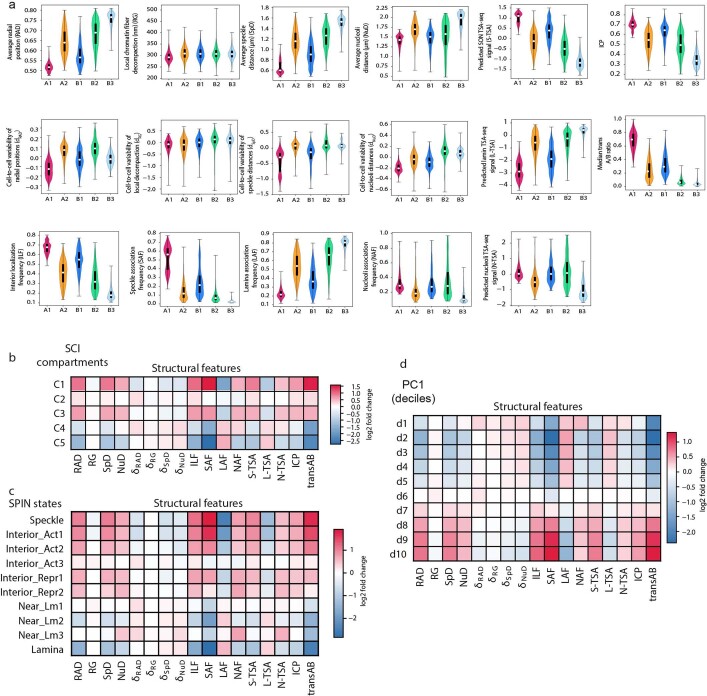
Structural features of chromatin in different subcompartments. **a**, Violin plots for the distributions of the 17 structural features calculated from the structure population for chromatin in different subcompartments. White circles and black bars in the violins show the median value and the interquartile range (IQR: Q3 – Q1). Whiskers show minima and maxima. Q1 and Q3 are the lower and upper quartile of the distribution. Numbers of regions used in each violin plot are: A1: 1,858, A2: 2,723, B1: 1,581, B2: 2,008, B3: 4,187. (**b-d)**: Fold-change enrichment of the 17 structural features for chromatin in different: **b**, SCI states^66^, **c**, SPIN states^67^, **d**, PC1 decile groups^38^. Structural feature abbreviations are as defined in Fig. 1.

Chromatins in different subcompartments are well separated in terms of their enrichment patterns for structural features, and thus represent distinct physical microenvironments (Fig. 6l, Extended Data Fig. 7a, and Methods) (speckle features are predicted without A1 subcompartment annotations). While A1 chromatin shows strong preferences in its nuclear microenvironment, particularly for speckle-associated features, A2 chromatin lacks clear location preferences, with high cell-to-cell variability in radial locations, overall weak enrichment patterns, and wide distributions of feature values (Fig. 6l and Extended Data Fig. 7a). Similarly, the three inactive B subcompartments are well distinguished in terms of their characteristic enrichment patterns. Indeed, these differences are so pronounced that we are able to predict Hi-C subcompartments from structural features alone without explicit considerations of chromatin interactions. Unsupervised *K*-means clustering based on structural feature vectors for compartment A chromatin predicts A1 and A2 subcompartment annotations with 94% accuracy. Chromatins in inactive subcompartments were predicted with an accuracy of 84% (Fig. 6m and Methods). These results are comparable in accuracy to supervised methods using Hi-C contact frequencies^65^. Our approach provides an alternative way of detecting subcompartment annotations while providing underlying structural interpretations.

Moreover, we confirmed our findings with other chromatin compartment annotations, such as SCI states^66^ and SPIN states^67^, which showed distinct structural enrichment patterns for each chromatin state (Extended Data Fig. 7b–d).

## Discussion

We introduce an approach to determine a population of single-cell 3D genome structures from ensemble Hi-C data. Our method predicts a host of structural features in single-cell models to provide information about the nuclear microenvironment of genomic regions in single cells, which is not available from ensemble Hi-C data itself. Therefore, our method expands the scope of Hi-C data analysis and is widely applicable to other cell types and tissues for which Hi-C data is available.

The models and derived structural features are a powerful resource to unravel relationships between genome structure and function. We found that cell-to-cell heterogeneity of structures varies by genomic loci and is a strong indicator of functional properties. Structurally stable chromatin in the A compartment is dominantly associated with nuclear speckles, and shows relatively high speckle association frequencies, a high *trans* A/B ratio, and the overall lowest average radial positions. These regions contain highly transcribed genes, are enriched for superenhancers and SON TSA-seq signals, and are replicated at the earliest time points. Moreover, these genomic regions compartmentalize in relatively large spatial partitions, formed by a high fraction of inter-chromosomal interactions. Chromatin in the A1 subcompartment is enriched in this category.

By contrast, active chromatin with high structural variability is characterized by a lack of preferences in nuclear locations. In a fraction of cells, these regions can be located in a silencing environment at the nuclear periphery; in others, it can be located towards the transcriptionally favorable interior. These genes show relatively low transcript frequencies, low inter-chromosomal contact probabilities with low *trans* A/B ratios, and intermediate replication timing (phases S2, S3). In TSA-seq experiments, most of these regions were identified as type II peaks, with intermediate TSA-seq values. We also noticed that these regions compartmentalize into relatively small spatial partitions, dominated by intra-chromosomal interactions. Chromatin in the A2 subcompartment is enriched in this category. It is possible that the high structural variability of these regions could be linked to functional heterogeneity between cells. For instance, although they are transcriptionally active, these regions have higher levels of the silencing H3K9me3 mark and reduced levels of the activating H3K9ac mark than active regions with low structural variability. Moreover, gene transcripts for these regions are found in a smaller fraction of cells and show lower transcriptional activity.

Interestingly, structural heterogeneity is also an indicator that can distinguish nucleoli- and lamina-associated chromatin in the B compartment. Genomic regions with low structural variability are dominantly associated with the lamina compartment, contain constitutive LADs and are enriched in the B3 subcompartment. Genomic regions with high structural variability are associated with nucleoli and pericentromeric heterochromatin and are enriched in the B2 subcompartment.

Our results suggest that nuclear speckles, together with the lamina compartment, are a major organizing factor in genome structure. Chromatin with low structural variability between cells is dominantly associated with either nuclear speckles or constitutive LADs. LADs are mostly located at the nuclear periphery while speckles are mostly excluded from the periphery^13,64^. Therefore, LADs and nuclear speckles provide structural anchor points at the periphery and nuclear interior. We hypothesize that A-LV and B-LV regions associated to these anchors act similarly to recently reported fixed points in the nuclear organization of mouse embryonic stem cells^7^.

**Fig. 7:**
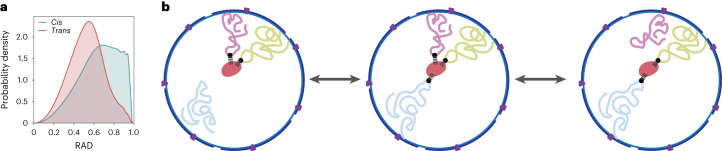
Inter-chromosomal interactions and speckle locations. **a**, Distributions of radial positions where *cis* and *trans* interactions occur in the models **b**, Scheme for the proposed effect of inter-chromosomal interactions on speckle (red) locations in the nucleus.

Moreover, the observed anticorrelation between inter-chromosomal contact probabilities and mean speckle distances suggests that speckles are hubs that facilitate inter-chromosomal interactions for active chromatin, confirming similar observations from SPRITE experiments^23^. The high fraction of inter-chromosomal interactions for speckle-associated chromatin could explain the preferential locations of speckles toward the nuclear interior. The probability of inter-chromosomal interactions increases towards the nuclear interior (Fig. 7a). If speckles associate with multiple chromosomes, their locations are more likely at the nuclear interior. Over time, dynamic interactions with multiple chromosomes may restrain their locations towards the interior (Fig. 7b). These cooperative effects could bias the global speckle distributions towards the nuclear interior.

Chromatins with highest and lowest transcriptional activity are distinguished by their nuclear microenvironment. The SAF shows the highest correlation with the gene transcription frequency^7,68–70^. Therefore, the interior preferences of highly activated genes could be a consequence of preferential locations close to nuclear speckles, which in turn have a stochastic preference towards the nuclear interior, confirming previous observations from TSA-seq experiments^13^. Chromatin replicated at the earliest time are also distinguished in their structural features from late-replicating chromatin. Moreover, our observations confirm that Hi-C subcompartments define physically distinct chromatin environments, some of which (such as A1) linked to associations with nuclear bodies.

In summary, our method defines the nuclear microenvironment of a genomic region by calculating a large number of structural features from 3D genome structures. The nuclear microenvironment of a gene can be linked to its functional potential in transcription and replication and thus is relevant for a better understanding of genome structure function relationships. These features can be calculated from Hi-C data, and thus are applicable to many different cell types.

## Methods

### Population-based 3D structural modeling

#### General description

Our goal is to generate a population of 10,000 diploid genome structures, so that the accumulated chromatin contacts across the entire population are statistically consistent with the contact probability matrix **A** = (*a*_*IJ*_)_(*N* × *N*)_ derived from Hi-C experiments^18,34^, with *I* and *J* as two chromatin regions in the genome. To achieve this goal, we utilize population-based modeling, our previously described probabilistic framework to de-multiplex the ensemble Hi-C data into a large population of individual genome structures of diploid genomes statistically consistent with all contact frequencies in the ensemble Hi-C data^33,34,32^.

The structure optimization is formulated as a maximum likelihood estimation problem solved by an iterative optimization algorithm with a series of optimization strategies for efficient and scalable model estimation^33,34,48^. Briefly, given a contact probability matrix **A** = (*a*_*IJ*_)_(*N* × *N*)_, we aim to reconstruct all 3D structures **X** = {***X***_1_, ***X***_2_…***X***_M_} in the population of *M* models, each containing 2 × *N* genomic regions for the diploid genome (at 200 kb base-pair resolution), and $\vec{x}_{im} \in {{\mathfrak{R}}}^{3}$*, i =* 1,…,2*N* as coordinates of all diploid genomic regions in model *m* (we use lowercase letters *i* and *i'* to indicate a given copy of the genomic region *I*). We introduce a latent indicator variable $\mathbf{W} = \left( w_{ijm} \right)_{2N}$ for complementing missing information (that is, missing phasing and ambiguity owing to genome diploidy). **W** is a binary-valued third-order tensor specifying the contacts of homologous genomic regions in each individual structure of the population, such that $\mathop{\sum }\limits_{m=1}^{M}{{\boldsymbol{W}}}^{m}/M={\boldsymbol{A}}$, with **W**^*m*^ = (*w*^*m*^)_2*N* × 2*N*_ such that ${w}_{{ij}}^{m}={w}_{{ijm}}$. We can jointly approximate the structure population (**X**) and the contact tensor (**W**) by maximizing the log-likelihood of the probability:

$${\rm{log }}P\left({\bf{X|A}}{\boldsymbol{,}}{\bf{W}}\right)={\rm{log }}P\left({\bf{A}}{\boldsymbol{,}}{\bf{W|X}}\right)$$

$$\mathrm{subject}\,\mathrm{to}\,\left\{\begin{array}{c}\mathrm{nuclear}\,\mathrm{volume}\,\mathrm{confinement}\\ \mathrm{excluded}\,\mathrm{volume}\\ \mathrm{chain}\,\mathrm{connectivity}\,\mathrm{restraint}\end{array}\right.$$

where

i. Nuclear volume constraint: all chromatin spheres are constrained to the nuclear volume with radius *R*_nuc_; ${\|{\vec{x}}_{{im}}\|}_{2}\le {R}_{\mathrm{nuc}},$ where ${\|{\vec{x}}_{{im}}\|}_{2}$ is the distance of the region *i* from the nuclear center in structure *m*.
ii. Excluded volume constraint: this constraint prevents overlap between two regions represented by spheres, defined by their excluded volume radii (*R*_ex_); ${d_{ijm} = \|{\vec{x}}_{{im}}-{\vec{x}}_{{jm}}\|}_{2}\ge 2{\times R}_{\mathrm{ex}}$.
iii. Polymer chain constraint: distances between two consecutive 200-kb spheres within the same chromosomes are constrained to their contact distance to ensure chromosomal chain integrity; ${\|{\vec{x}}_{\left(i+1\right)m}-{\vec{x}}_{{im}}\|}_{2}\le 2{\times R}_{\mathrm{soft}}$, where ${R}_{\mathrm{soft}}=\,2{\times R}_{\mathrm{ex}}$.

Our modeling pipeline uses a step wise iterative process in which the optimization hardness is gradually increased by adding contacts with decreasing contact probabilities in the input matrix. The iterative optimization procedure involves two steps, each optimizing local approximations of the likelihood function: (1) assignment step (A-step)—given the estimated structures **X**^*k*^ at step *k*, estimate **W**^k^; and (2) modeling step (M-step)—given the estimated **W**^*k*^, generate model population **X**^*k*+1^ at step *k* + 1 that maximizes likelihood to observe **W**. Structures in the M-step are calculated using a combination of optimization approaches, including simulated annealing molecular dynamics simulations.

Moreover, during each optimization cycle, we also use iterative refinement steps, a methodological innovation for effective reassignment of restraints during the optimization process, which allows genome structure generation at higher resolution and improved accuracy in comparison to our previous approach^33,34^ (see iterative refinement method in Supplementary Information).

After 11 iterations, our method converged and the genome-wide contact probabilities from the structure population agreed with those from the Hi-C experiment.

#### Genome representation

The nucleus is modeled as a sphere with 5-μm radius (*R*_nuc_)^34^. Chromosomes are represented by a chromatin chain model at 200-kb base-pair resolution. Each 200-kb chromatin region, in the diploid genome, is modeled as a sphere, defined by an excluded volume radius (*R*_ex_ = 118 nm). *R*_ex_ is estimated from the sequence length, the nuclear volume and the genome occupancy (40%), as described in ref. ^34^. The full diploid genome is represented with a total of 30,332 spheres.

#### Random starting configurations

Optimizations are initiated with random chromosome configurations. Chromatin regions are randomly placed in a bounding sphere proportional to its chromosome territory size and randomly placed within the nucleus.

#### Comparison between contact frequency maps from Hi-C experiment and model population

To quantify the agreement between Hi-C experiment and model population, we perform the following analyses:

1. Comparison between input and output Hi-C maps are evaluated by Pearson and stratum adjusted (SCC)^57^ correlation coefficients (Supplementary Table 1).
2. Restraint residual. On average about 175,304 contact restraints are imposed in each of the 10,000 structures. The restraint residual of each contact restraint between loci *k* and *l* is calculated as: ${\eta }_{kl}=\frac{{d}_{kl}-D}{D}$, where *d*_*kl*_ is the distance between the contact loci in the model, and *D* is the target contact distance (2 × *R*_soft_).
3. Residual ratio. The residual ratio Δ*r* is defined as:
  $${\Delta r}_{{kl}}=\,\left({f}_{{kl}}^{\,\mathrm{input}}-\,{f}_{{kl}}^{\,\mathrm{model}}\right)/{f}_{{kl}}^{\,\mathrm{input}}$$
  with ${f}_{{kl}}^{\,\mathrm{input}}$ and ${f}_{{kl}}^{\,\mathrm{model}}$ as the contact probabilities between regions *k* and *l* from experiment and models, respectively. Residual ratios are very small, and centered at a median of 0.03 (mean = −0.05) for intra-chromosomal and 0.001 (mean = −0.002) for inter-chromosomal contacts (Supplementary Fig. 23), showing agreement between experiment and model.
4. Prediction of missing Hi-C data from sparse data model. A sparse Hi-C input data set is generated by randomly removing 50% of the non-zero data entries from the Hi-C contact frequency matrix.

#### Comparison of simulated single cell chromosome structures with those from DNA-MERFISH imaging

Preprocessing of the DNA-MERFISH dataset^6^: please refer to the methods in Boninsegna et al.^32^.

Preprocessing Dip-C dataset^25^: we collected both homologous chromosome copies from each of the 16 single cells. To match our model resolution, we generated 200-kb-resolution models by averaging coordinates of loci that map to 200-kb bins.

Calculation and comparison of distance matrices: please refer to Methods in Boninsegna et al.^32^.

### Robustness and converge analysis

#### Replicates

Technical replicates are calculated from different random starting configurations. Resulting contact frequency maps and the average radial positions of all chromatin regions between replica populations are nearly identical (Supplementary Fig. 24). All observed structural features discussed in this paper are reproduced in the technical replicate population.

#### Population size

To assess convergence with respect to population size, we generated 5 populations with 50, 100, 1,000, 5,000, or 10,000 structures. Chromatin contact frequencies and structural features for each structure populations were compared against results with a population size of 10,000 structures. At a population of 1,000 structures, a size much smaller than our target population, contact frequency values and average radial positions were already converged at a very high correlation with those from a 10,000-structure population (Supplementary Fig. 25).

### Chromatin interaction networks and identification of spatial partitions

#### Building chromatin interaction networks

A chromatin interaction network (CIN) is calculated for each model and for chromatin in each subcompartment separately as follows (Supplementary Fig. 26): each vertex represents a 200-kb chromatin region. An edge between two vertices *i* and *j* is drawn if the corresponding chromatin regions are in physical contact in the model, if the spatial distance *d*_*ij*_ ≤ 2 × *R*_soft_).

#### Network properties

Maximal clique enrichment: A clique is a subset of nodes in a network where all nodes are adjacent to each other and fully connected. The maximal clique refers to the clique that cannot be further enlarged. The number of maximal cliques, *c*, is calculated using the graph_number_of_cliques function in the NetworkX python package^71^. The maximal clique enrichment (MCE) of the subcompartment *s* in the structure *m* is calculated as:

$${{MCE}}_{s,m}=\,\frac{{c}_{s,m}}{\frac{1}{10}\mathop{\sum }\limits_{r=1}^{10}{c}_{r,m}}$$

Where *c*_*s*,*m*_ is number of maximal cliques for subcompartment *s* in structure *m*; and *c*_*r*,*m*_ is the number of maximal cliques of a CIN constructed from randomly shuffled subcompartment regions in the same structure *m*. High MCE values show formation of a structural subcompartment with high connectivity between 200-kb regions of the same state.

Neighborhood connectivity: To calculate the neighborhood connectivity (NC) of a subcompartment CIN, we first calculate the average neighbor degree for each node using the average_neighbor_degree function in the NetworkX python package^71^. The overall neighborhood connectivity of the subcompartment *s* in the structure *m* is then calculated as:

$${{NC}}_{s,m}=\frac{1}{{N}_{s,m}}\mathop{\sum }\limits_{j=1}^{{N}_{s,m}}{\deg }_{j}\,$$

where *N*_*s,m*_ is the number of nodes in the CIN of the subcompartment *s* in the structure *m*, and *deg*_*j*_ is the average neighbor degree of node *j*.

#### Identifying spatial partitions via Markov clustering

Spatial partitions of subcompartments are identified by applying the Markov Clustering Algorithm (MCL)^72^, a graph clustering algorithm, which identifies highly connected subgraphs within a network. MCL clustering is performed for each subcompartment CIN in each structure by using the mcl tool in the MCL-edge software^72^. Unless otherwise noted, the 25% smallest subgraphs (with less than 7 nodes, many of those being singletons) are discarded from further analysis, to focus on highly connected subgraphs. The highly connected subgraphs are referred to as ‘spatial partitions’ throughout the text.

In addition to subcompartment partitions, we also predict speckle and nucleoli partitions as follows:

#### Speckle partitions

Case 1: Predictions of speckle locations with knowledge of A1 subcompartment annotations.

Speckle locations are identified as the geometric center of A1 spatial partitions identified by Markov clustering of A1 CINs. In each structure, only A1 spatial partitions with sizes larger than three nodes (chromatin regions) are considered for downstream analysis.

Case 2: Predictions of speckle locations without knowledge of subcompartments.

We first identify chromatin expected to have high speckle association. These regions are identified as those with unusually low and stable interior radial positions. We select 10% chromatin regions with the lowest average radial positions (78.4% of these regions are part of the A1 subcompartment). We then generate CINs for the selected group of chromatin regions in each structure of the population. Approximate speckle locations are then identified as the geometric center of the resulting spatial partitions identified by Markov clustering of the CINs. Only spatial partitions with sizes larger than three nodes (chromatin regions) are considered for downstream analysis.

Case 3: Predictions using locations of A2 partition centers.

For comparison, we also identify speckle locations as the geometric center of A2 spatial partitions identified by Markov clustering of A2 CINs similar to case 1. In each structure, only A2 spatial partitions with sizes larger than three nodes (chromatin regions) are considered for downstream analysis.

#### Nucleoli partitions

Following the same protocol as in case 2 for speckle partitions, we first identify chromatin expected to have high nucleoli association. These regions are identified as those previously reported nucleoli-associated domain (NAD)^73^ regions and nucleolus organizing regions (NOR, on short arms of chromosomes 13, 14, 15, 21, and 22). Using these regions, we generate CINs in each structure of the population. Approximate nucleoli locations are then identified as the center of mass of the resulting spatial partitions identified by Markov clustering of the CINs. Only the top 25% largest spatial partitions are used as predicted nucleoli. For NOR regions, we use the first 25 restrained 200-kb regions that are closest in sequence to NOR regions in these five chromosomes, as NOR regions do not have Hi-C data and they are not restrained during the modeling protocol.

#### Properties of partitions

Size of partitions: The size of a spatial partition is calculated as 0.2 × *N* Mb, where *N* is the number of nodes in the partition that represents a 0.2-Mb region.

Fraction of inter-chromosomal edges (contacts): For each spatial partition, the inter-chromosomal edge fraction (ICEF) is calculated as:

$${ICEF}=\,\frac{{E}_{\mathrm{inter}}}{{E}_{\mathrm{intra}}+{E}_{\mathrm{inter}}}$$

where *E*_intra_ and *E*_inter_ are the number of intra- and inter- edges in the partition, respectively.

### Structural features

Unless otherwise noted, mean values of structural features for each genomic region *I* are calculated from 2 copies (*i* and *i'*) and 10,000 structures (total 20,000 configurations) in the following structural feature calculations.

### Mean radial position (RAD, no. 1)

Radial position of a chromatin region *i* in structure *m* is calculated as:

$${r}_{i,m}=\frac{{d}_{i,m}}{{R}_{\mathrm{nuc}}}$$

where *d*_*i,m*_ is the distance of *i* to the nuclear center, and *R*_nuc_ is the nucleus radius which is 5 μm. *r*_*i,m*_ = 0 means the region *i* is at the nuclear center, while *r*_*i,s*_ = 1 means it is located at the nuclear surface.

### Local chromatin fiber decompaction (RG, no. 2)

The local compaction of the chromatin fiber at the location of a given locus is estimated by the radius of gyration (RG) for a 1 Mb region centered at the locus (that is, comprising +500 kb up- and 500 kb downstream of the given locus). To estimate the RG values along an entire chromosome we use a sliding-window approach over all chromatin regions in a chromosome.

The RG for a 1 Mb region centered at locus *i* in structure *m* is calculated as:

$${{RG}}_{i,m}=\,\mathop{\sum }\limits_{j=1}^{N}{{d}_{j,m}}^{2}$$

where *N* is the number of chromatin regions in the 1-Mb window, and *d*_*j,m*_ is the distance between the chromatin region *j* to the center of mass of the 1-Mb region, in structure *m*.

### Mean gene–speckle and gene–nucleolus distances (SpD and NuD, nos. 3 and 4)

For each 200-kb region, the closest speckle partition (or nucleolus partition) in each single structure is identified and the center-to-center distance is calculated (from the center of the region to the geometric center of the partition). The distances across the population are then averaged for each region to calculate mean speckle (or nucleolus) distances.

### Cell-to-cell variability of features (*δ*_RAD_, *δ*_RG_, *δ*_SpD_, and *δ*_NuD_, nos. 5–8)

Cell-to-cell variability of any structural feature *F* (${\delta }_{I}^{\mathrm{RAD}}$ for radial positions, ${\delta }_{I}^{\mathrm{SpD}}$ speckle distances, ${\delta }_{I}^{\mathrm{NuD}}$ nucleoli distances, and ${\delta }_{I}^{\mathrm{RG}}$ local decompaction) for a chromatin region *I* is calculated as:

$${\delta }_{I}^{F}={\log }_{2}\,\frac{{\sigma }_{I}^{F}}{\bar{{\sigma }^{F}}}$$

where ${\sigma }_{I}^{F}$ is the s.d. of the values for structural feature *F* calculated from both homologous copies *i* and *i'* of the region *I* across all 10,000 genome structures in the population; $\bar{{\sigma }^{F}}$ is the mean s.d. of the feature value calculated from all regions within the same chromosome of region *I*. Positive ${\delta }_{I}^{F}$ values (${\delta }_{i}^{F} > 0$) result from high cell-to-cell variability of the feature (for example radial position); negative values (${\delta }_{i}^{F} < \,0$) indicate low variability.

Regions in the A compartment with positive and negative ${\delta }_{I}^{{RAD}}$ are called A-HV (high variability) and A-LV (low variability), respectively. Likewise, regions in the B compartment with positive and negative ${\delta }_{I}^{{RAD}}$ are called B-HV and B-LV, respectively. The number of 200-kb regions in each group are 3,164, 2,731, 3,839, and 3,918 for A-LV, A-HV, B-LV, and B-HV, respectively.

### Interior localization frequency (ILF, no. 9)

For a given 200-kb region, the interior localization frequency (ILF) is calculated as:

$${{ILF}}_{I}=\,\frac{{n}_{r_I < 0.5}}{M}$$

where ${{n}_{rI < 0.5}}$ is the number of structures where either copy of the region *I* has a radial position lower than 0.5, and *M* is the total number of structures which is 10,000 in our population.

### Nuclear-body association frequencies (SAF, LAF, and NAF, nos. 10–12)

For a given 200-kb region, the association frequency to nuclear bodies (SAF, LAF, and NAF for speckle, lamina, and nucleoli association frequencies, respectively) is calculated as:

$${{SAF} (\text{or } LAF \text{ or } NAF)}_{I}=\,\frac{{n}_{{d}_{i} < {d}_{t}}+{n}_{{d}_{{i}^{{\prime} }} < {d}_{t}}}{2M}$$

where *M* is the number of structures in the population (two homologous copies of each chromosome are present per structure); ${n}_{{d}_{i} < {d}_{t}}$ and ${n}_{{d}_{{i}^{{\prime} }} < {d}_{t}}$ are the number of structures, in which region *i* and its homologous copy *i*′ have a distance to the nuclear body of interest (NB) smaller than the association threshold, *d*_*t*_, respectively. The *d*_*t*_s are set to 500 nm, 0.35 × *R*_nuc_, and 1,000 nm for SAF, LAF, and NAF, respectively. We tried different distance thresholds, and the selected thresholds resulted in the best correlations with experimental data. For SAF and NAF calculations, we use the predicted speckle and nucleolus partitions to calculate distances (see ‘Identifying spatial partitions via Markov clustering’). For LAF, we use the direct distances of regions to the nuclear envelope. For all association frequency calculations, we calculate distances from the surface of the region to the center-of-mass of the partition or to the surface of the nuclear envelope.

### TSA-seq (S-TSA, L-TSA, N-TSA, nos. 13–15)

To predict TSA-seq signals for speckle, nucleoli, and lamina from our models, we use the following equation:

$${{sig}}_{i}=\,\frac{1}{M}\mathop{\sum }\limits_{m=1}^{M}\mathop{\sum }\limits_{l=1}^{L}{e}^{-{R}_{0}\|{d}_{{il}}\|}$$

where *M* is the number of models, *L* is the number of predicted speckle locations in structure *m*, *d*_*il*_ is the distance between the region *i* and the predicted nuclear body location *l*, and *R*_0_ is the estimated decay constant in the TSA-seq experiment^13^ which is set to 4 in our calculations. The normalized TSA-seq signal for region *i* then becomes:

$${\mathrm{predicted}\;\mathrm{TSA}{\hbox{-}}\mathrm{seq}\;\mathrm{signal}}_{i}=\log \left(\frac{{{sig}}_{i}}{\overline{{sig}}}\right)$$

where $\overline{{sig}}$ is the mean signal calculated from all regions in the genome. The predicted signal is then averaged over two copies for each region. The predicted speckle and nucleoli partitions are used for distance calculations (see ‘Identifying spatial partitions via Markov clustering’). For lamina TSA-seq, we use direct distances of each 200-kb chromatin region to the nuclear surface in each structure, which is calculated as (1 − *r*_*i,m*_) × *R*_nuc_, where *r*_*i,m*_ is the radial position of the 200-kb region *i* in structure *m* and *R*_nuc_ is the nucleus radius, which is set to 5 μm.

### Mean inter-chromosomal neighborhood probability (ICP, no. 16)

For each target chromatin region *i*, we define the neighborhood {*j*} if the center-to-center distances of other regions {*j*} to the target region are smaller than 500 nm, which can be expressed as a set; *Ne*_*i*_ = {*j*: *j* ≠ *i,* $d_{ij}$ *<* 500 nm}. Inter-chromosomal neighborhood probability (ICP) is then calculated as:

$${{ICP}}_{I}=\frac{1}{2M\,}\mathop{\sum }\limits_{m=1}^{M}\mathop{\sum }\limits_{i=1}^{2}\frac{{n}_{{inter}}(m,i)}{{n}_{{inter}}\left(m,i\right)+{n}_{{intra}}(m,i)}$$

where *M* is the number of structures, *n*_*intra*_ (*m,i*) and *n*_*inter*_ (*m,i*) are the number of intra- and inter-chromosomal regions in the set *Ne*_*i*_ in structure *m* for region *i*.

### Median *trans* A/B ratio (no. 17)

For each chromatin region *i*, we define the trans neighborhood {*j*} if the center-to-center distances of other regions from other chromosomes to itself are smaller than 500 nm, which can be expressed as a set; ${{Ne}}_{i}^{t}=\{j:\,{{chrom}}_{i}\ne \,{{chrom}}_{j},\,{d}_{{ij}} < 500{nm}\}$. The *trans* A/B ratio is then calculated as:

$${Trans}\,\mathrm{A/B}\,{\mathrm{ratio}}_{i}=\frac{{n}_{A}^{t}}{{n}_{B}^{t}}$$

where ${n}_{A}^{t}$ and ${n}_{B}^{t}$ are the number of trans A and B regions in the set ${{Ne}}_{i}^{t}$ for region *i*. The median of the *trans* A/B ratios for a region is then calculated from all the *trans* A/B ratios of the homologous copies of the region observed in all the structures of the population. The values are then rescaled to have values between 0 and 1.

### Data analysis

The analyses and most of the figure panels were performed using custom Python scripts (matplotlibv3.4 (ref. ^74^), Scikit-learnv1.0 (ref. ^75^), scipyv1.5 (ref. ^76^), and networkxv2.3 (ref. ^71^)) together with the publicly available alabtools platform (https://github.com/alberlab/alabtools). The remaining panels and the final figures were assembled using Adobe Illustrator. Correlations between input and output contact matrices were calculated using HiCRep^57^ (https://github.com/TaoYang-dev/hicrep). Spatial partitions were identified using the MCL algorithm^72^ (https://micans.org/mcl/). Chromatin interaction networks were visualized with Cytoscape^77^. Images of 3D genome structures were generated using UCSF Chimera1.13 (ref. ^78^). For all other analysis, please refer to the Supplementary Information.

### Reporting summary

Further information on research design is available in the Nature Portfolio Reporting Summary linked to this article.

## Online content

Any methods, additional references, Nature Portfolio reporting summaries, source data, extended data, supplementary information, acknowledgements, peer review information; details of author contributions and competing interests; and statements of data and code availability are available at 10.1038/s41594-023-01036-1.

### Supplementary information

Supplementary Information Supplementary Figs. 1–26, Tables 1 and 2, Discussion.

Reporting Summary

## Figures and Tables

**Extended Data Table 1:**
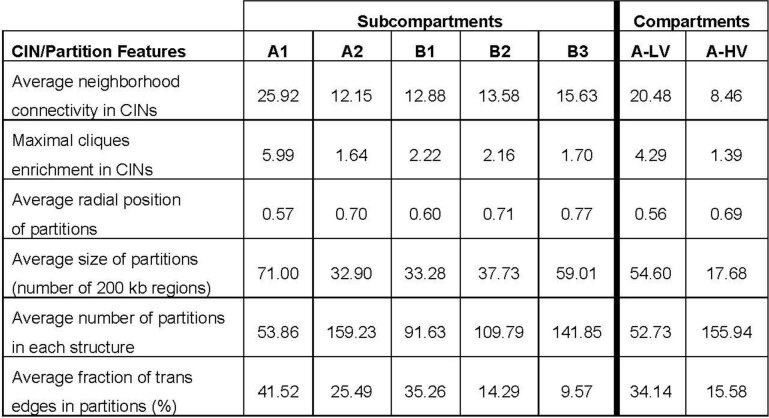
Properties of subcompartment interaction networks and spatial partitions

Population averages of features for chromatin interaction networks (CIN) and spatial partitions of chromatin in different subcompartments (Methods).

**Extended Data Table 2:**
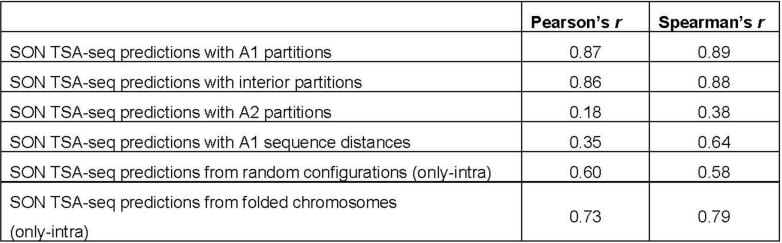
Genome-wide Pearson and Spearman correlations between experimental and predicted SON TSA-seq data using different approaches

All p-values are ~0. Chromosome X is discarded from genome-wide correlation calculations.

## Data Availability

The genome structure population and genome-wide structural features are available at 10.5281/zenodo.7352276. The accession codes for the experimental data used in our analyses are as follows: GEO: GSE63525 (Hi-C), GSE63525 (subcompartments), GSE81553 (SON TSA-seq), GSE81553 (lamin-B1 TSA-seq), GSE56465 (single cell lamina DamID), GSM1480326 (GRO-seq), GSE135882 (GPSeq), GSM923451 (Repli-seq), GSM3596321 (scRNA-seq); 4DN: 4DNFIGL8MCSJ (lamin-B1 pA-DamID), 4DNFILYQ1PAY (compartments); ENCODE: ENCFF313LYI, ENCFF171MDW, ENCFF776DPQ, ENCFF309OEW, ENCFF028KBY, ENCFF601YET, ENCFF831ZHL, ENCFF039HDL, ENCFF340JIF, ENCFF803DJF, ENCFF683HCZ (ChIP–seq, histone modifications), https://zenodo.org/record/3928890 (DNA-MERFISH imaging). The complete list of the datasets used in this study and their accession numbers are also tabulated in Supplementary Table 2.

## Code availability

The software used to generate the genome structure population and the accompanying documentation are available at https://github.com/alberlab/igm.
