# Supplementary material for: Evaluating the role of the nuclear microenvironment in gene function by population-based modeling
Source: Nat Struct Mol Biol. 2023 Aug 14;30(8):1193–206. doi: 10.1038/s41594-023-01036-1 (PMC10442234; doi:10.1038/s41594-023-01036-1)
Supplement: Supplementary file 1 — Supplementary Figs. 1–26, Tables 1 and 2, Discussion. [file 41594_2023_1036_MOESM1_ESM.pdf]

nature structural & molecular biology

Article

https://doi.org/10.1038/s41594-023-01036-1

# Evaluating the role of the nuclear microenvironment in gene function by population-based modeling

In the format provided by the authors and unedited

# Table of Contents


1. **Supplementary Figures and Tables**

- **Fig. S1** Correlation between the mean distance to the nearest speckle and the inter-chromosomal contact probability (ICP) of genomic regions.
- **Fig. S2-22**. Structural feature profiles for Chr2-22.
- **Fig. S23.** Residual ratios
- **Fig. S24.** Comparison of replicate structure populations
- **Fig. S25.** Population size convergence plots
- **Fig. S26.** Chromatin interaction networks for subcompartments

- **Table S1**. Pearson and stratum adjusted correlation coefficients between input/output for each chromosome.
- **Table S2**. List of experimental data sources used in this work (and relating accession numbers)

2. **Analyses related to structural features**

3. **Comparison of gene expression with structural features**

4. **Other structural analyses**

5. **Preprocessing Hi-C data**

6. **Iterative refinement**

7. **Mapping experimental data to 200 kb**

8. **3D DNA FISH experiments**

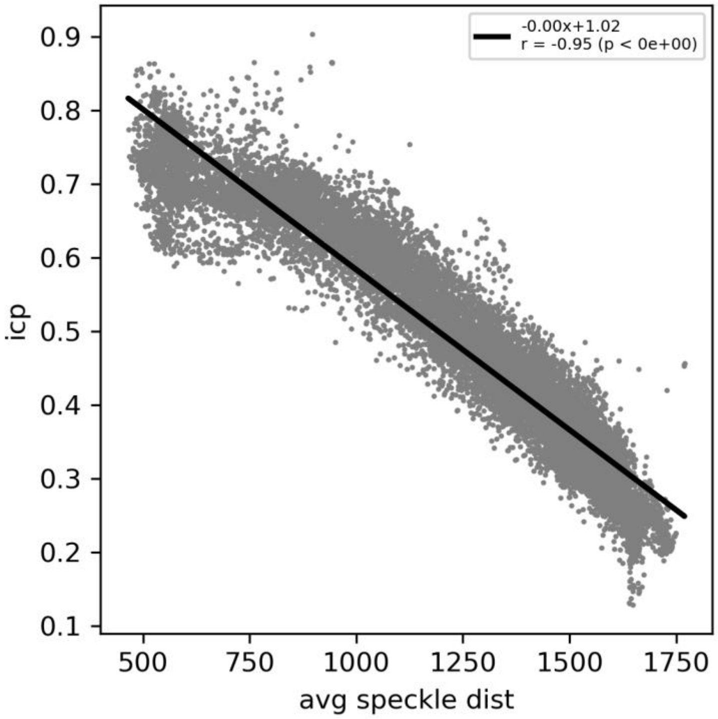


**Fig. S1.** Pearson correlation (*r*= -0.95, p=~0) between the mean distance to the nearest speckle and the inter-chromosomal contact probability (ICP) of genomic regions.

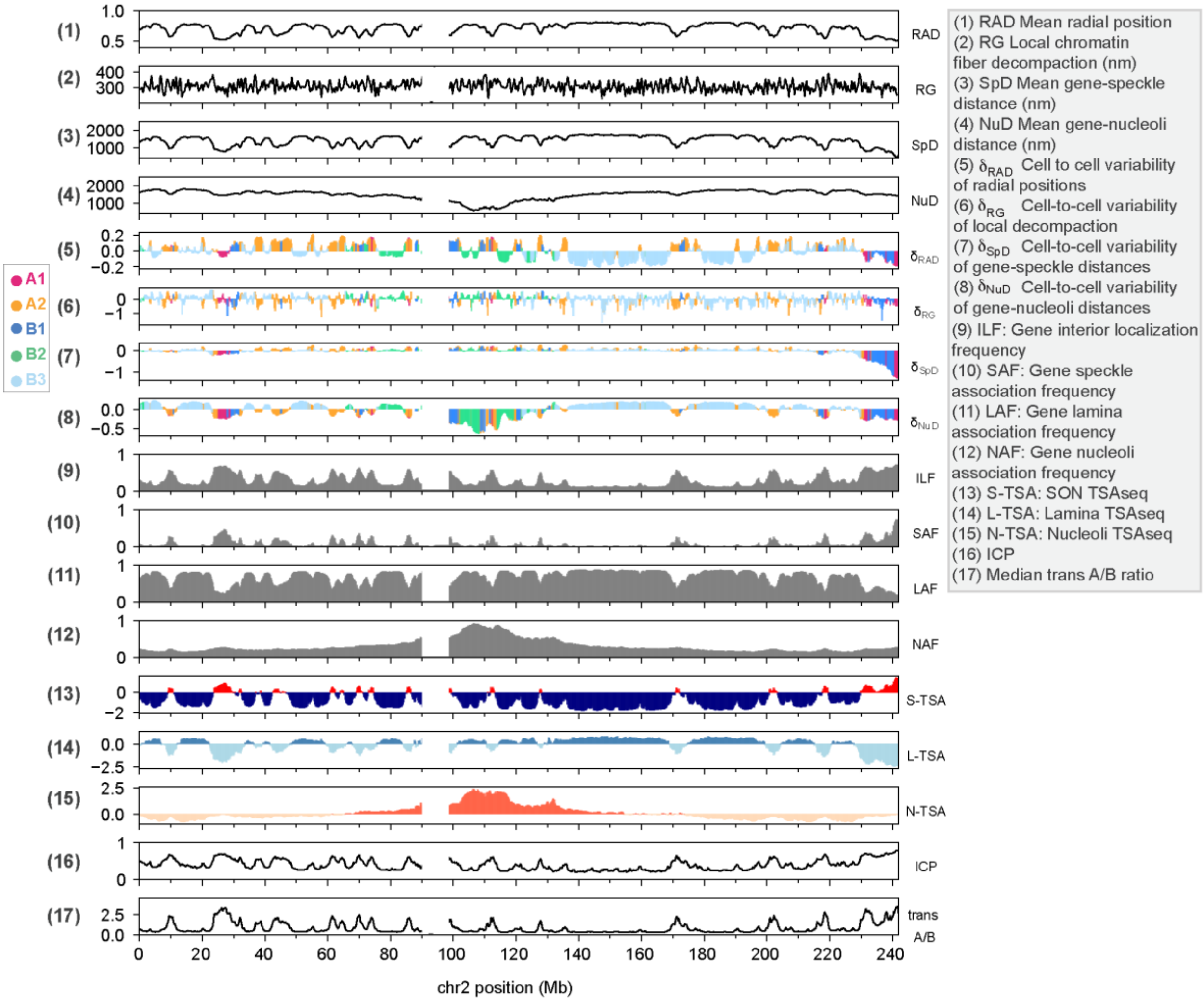


**Fig. S2.** Structure feature profiles for chromosome 2.

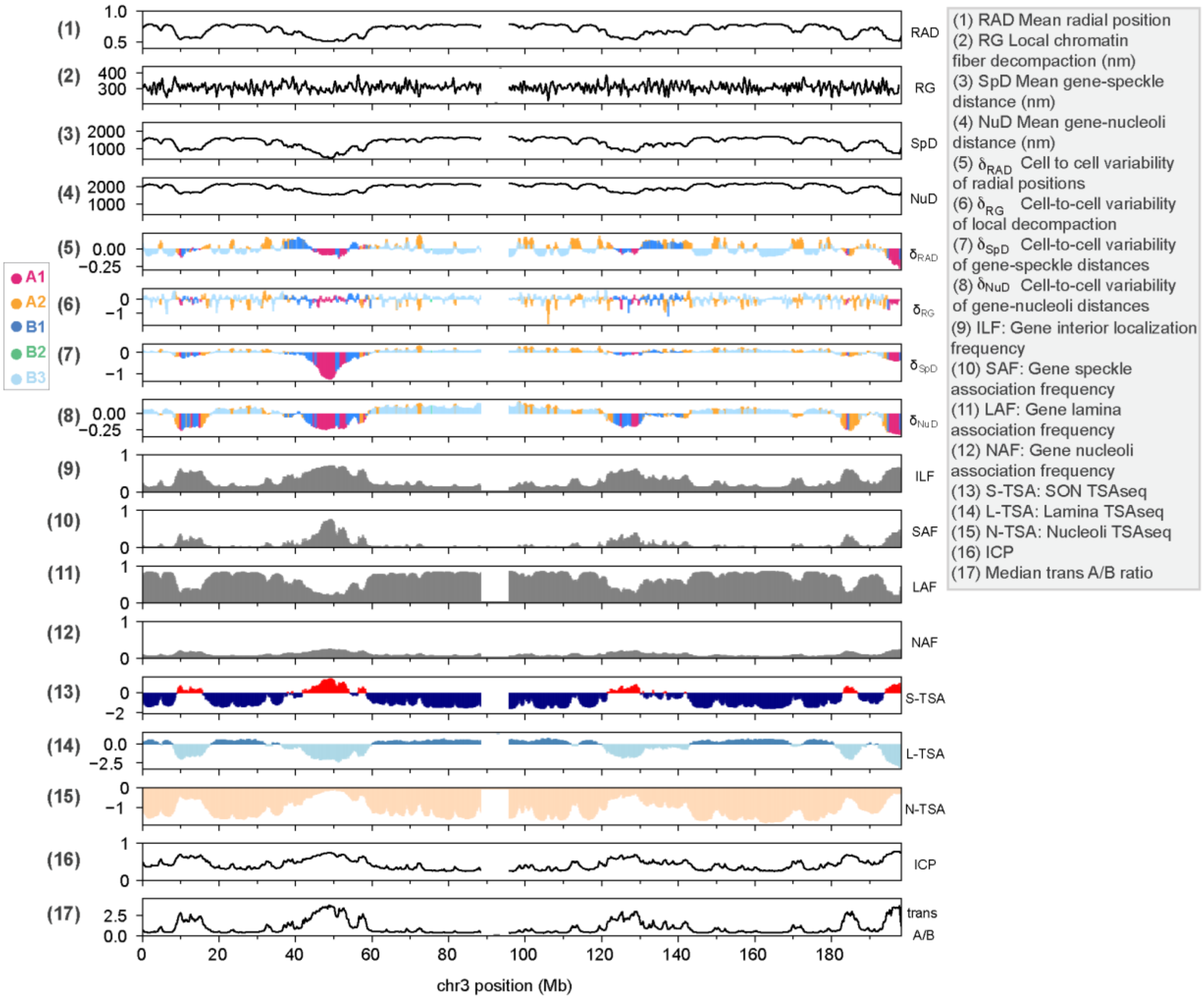


**Fig. S3.** Structure feature profiles for chromosome 3.

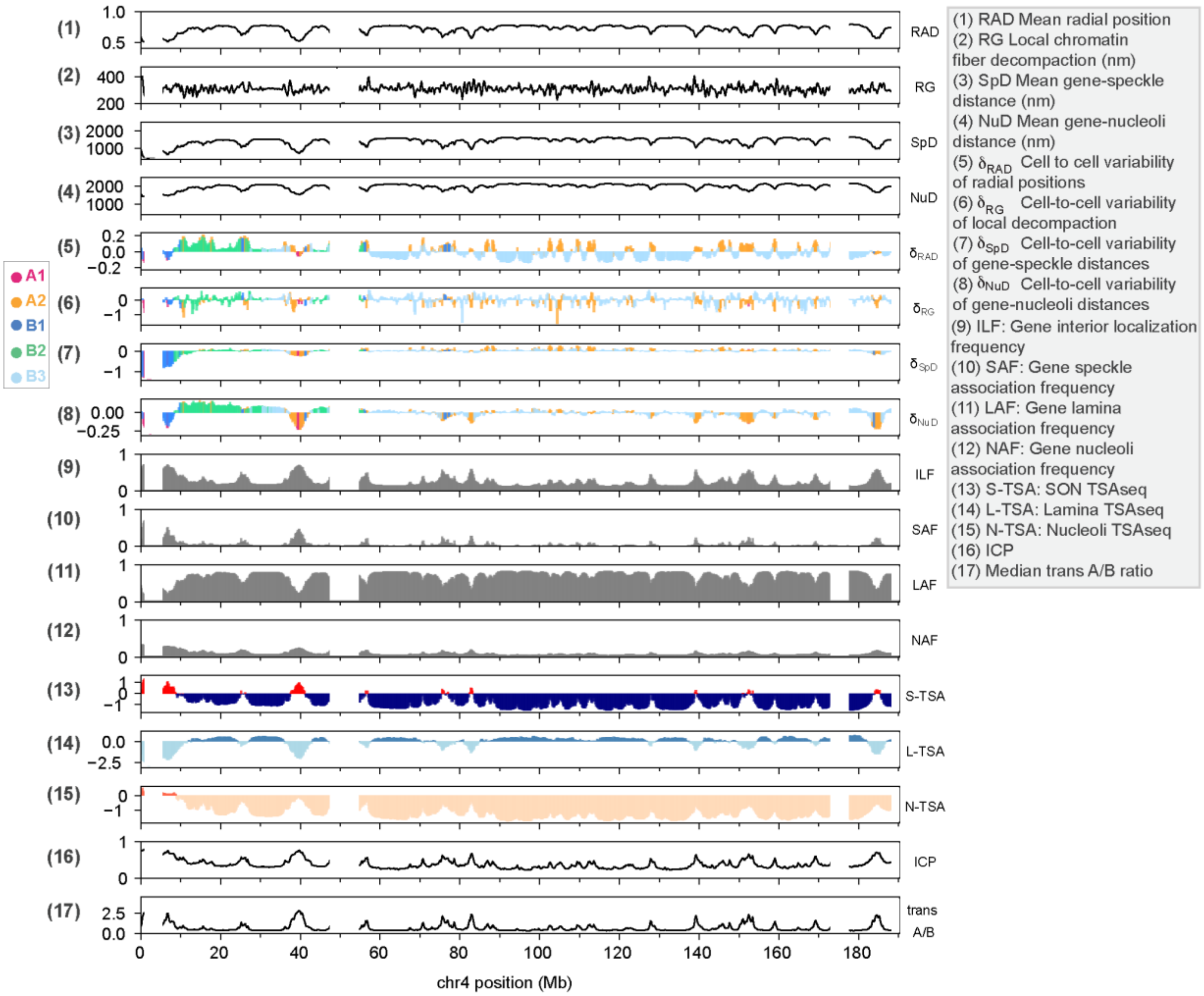


**Fig. S4.** Structure feature profiles for chromosome 4.

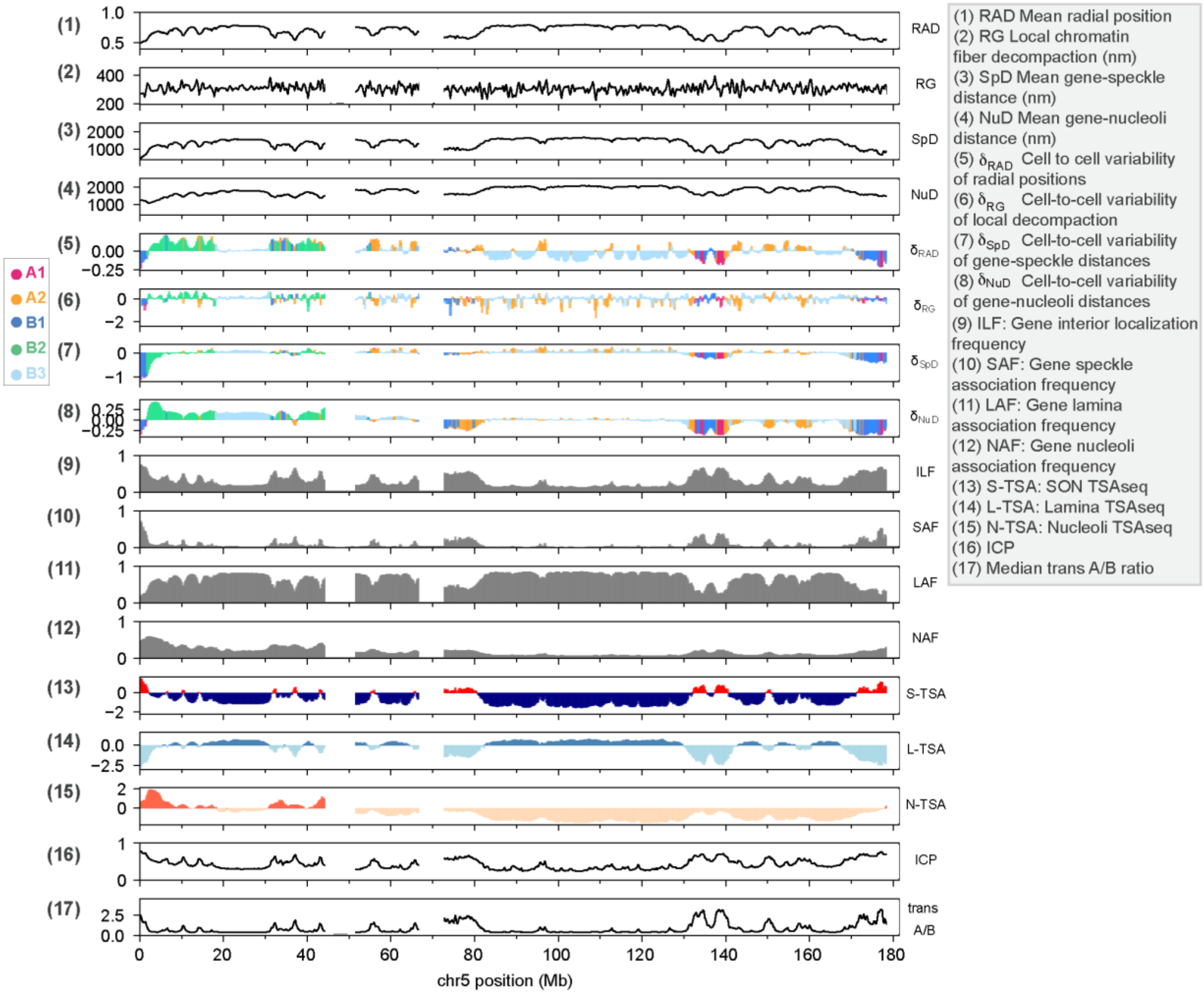


**Fig. S5.** Structure feature profiles for chromosome 5.

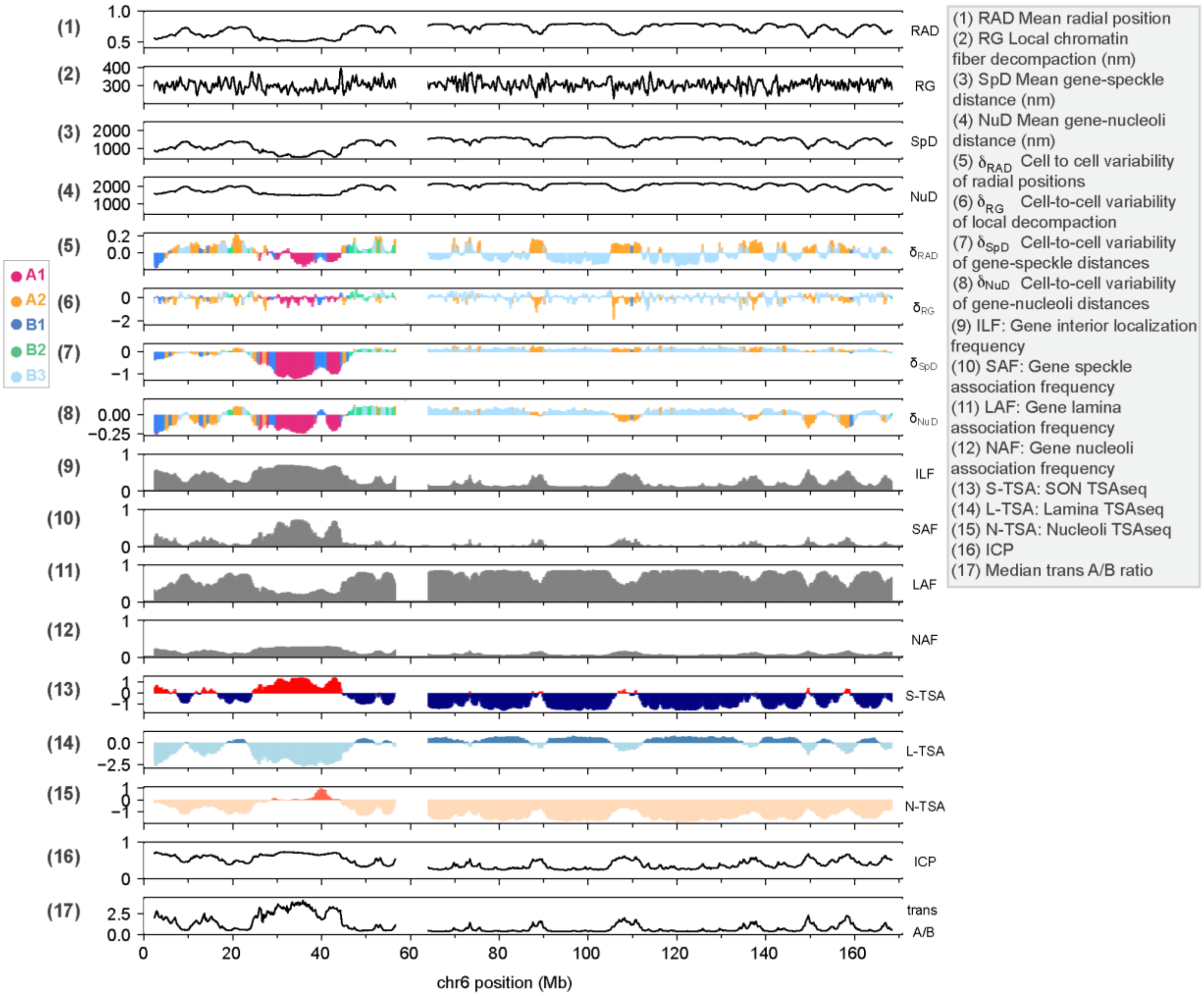


**Fig. S6.** Structure feature profiles for chromosome 6.

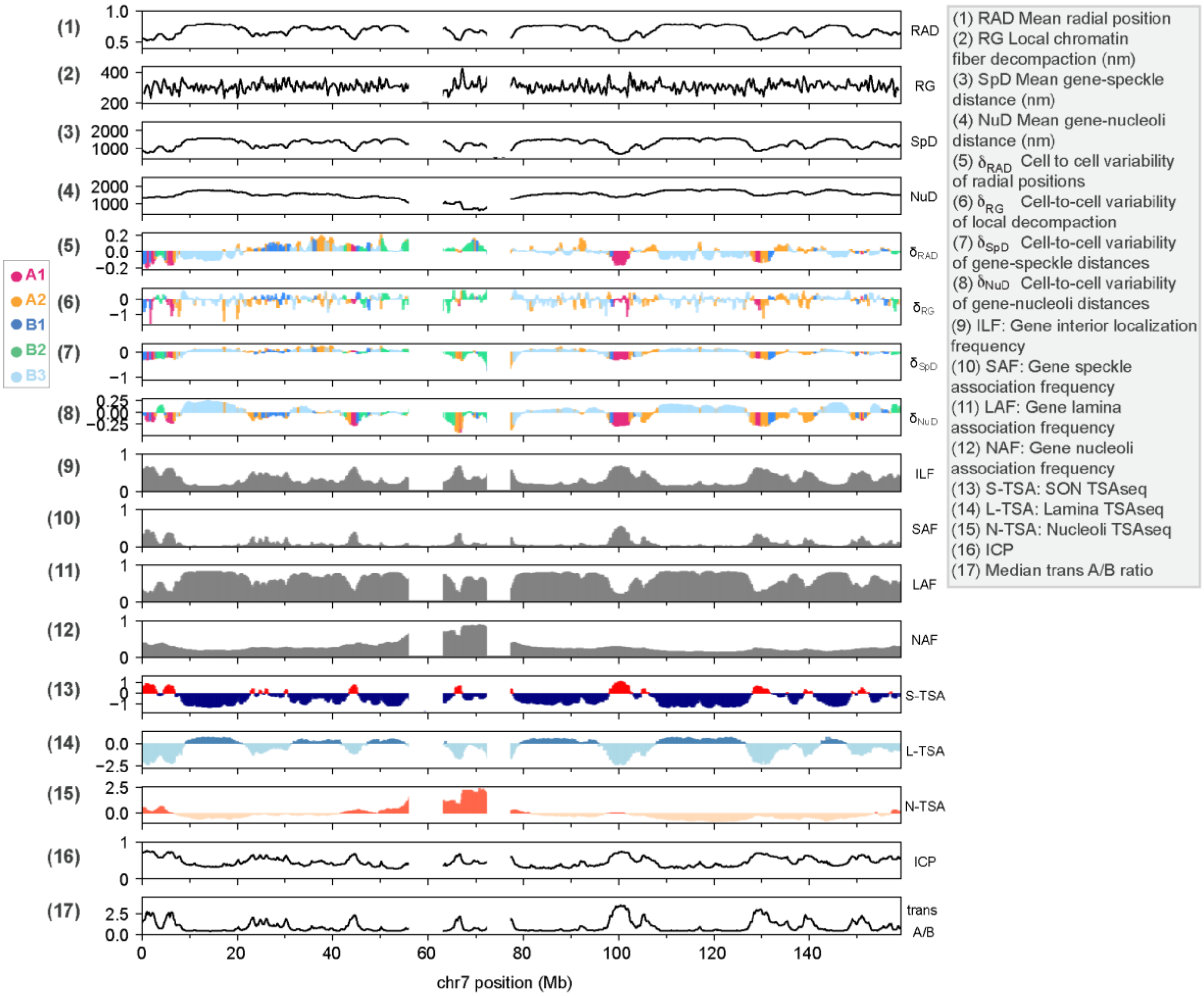


**Fig. S7.** Structure feature profiles for chromosome 7.

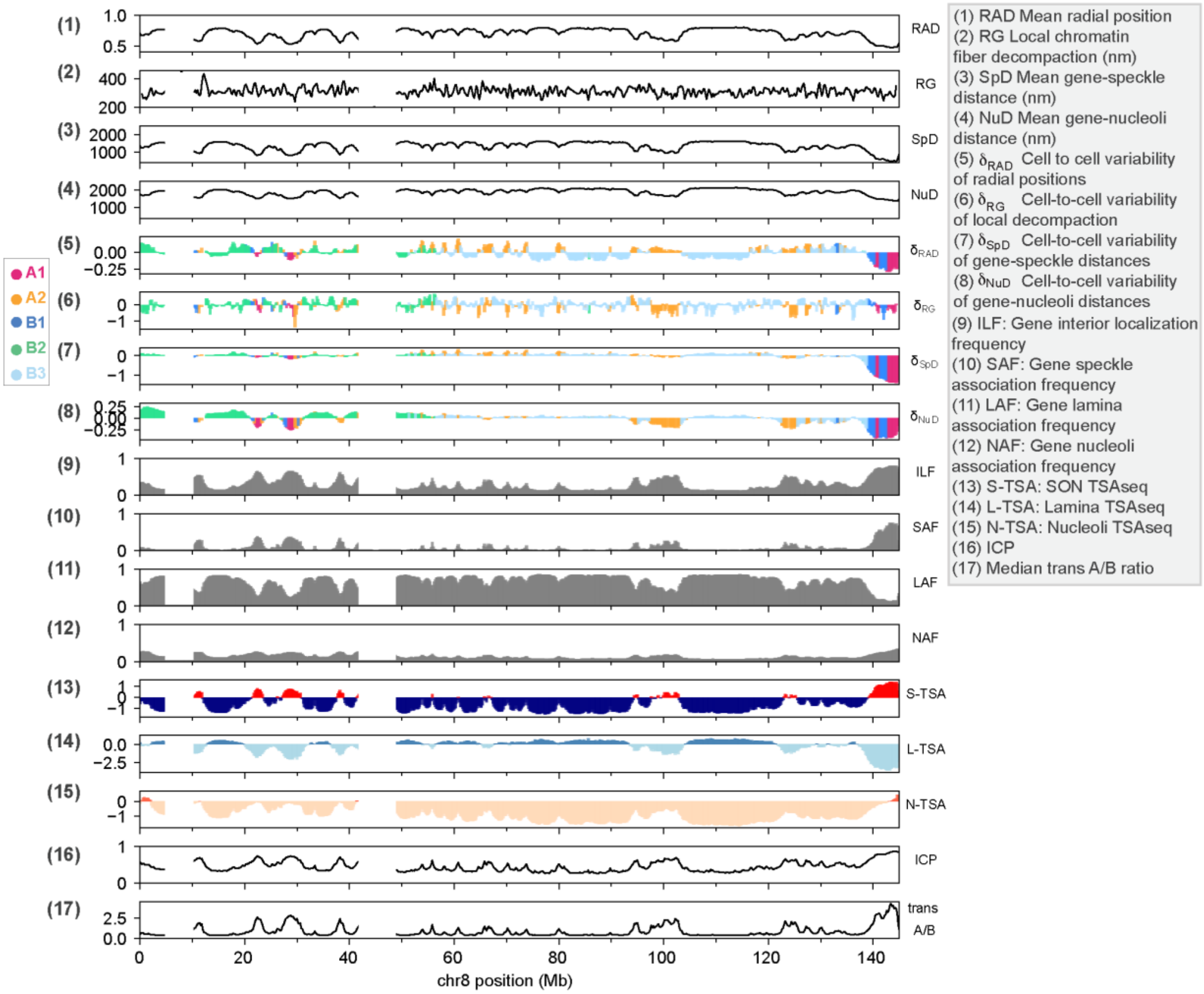


**Fig. S8.** Structure feature profiles for chromosome 8.

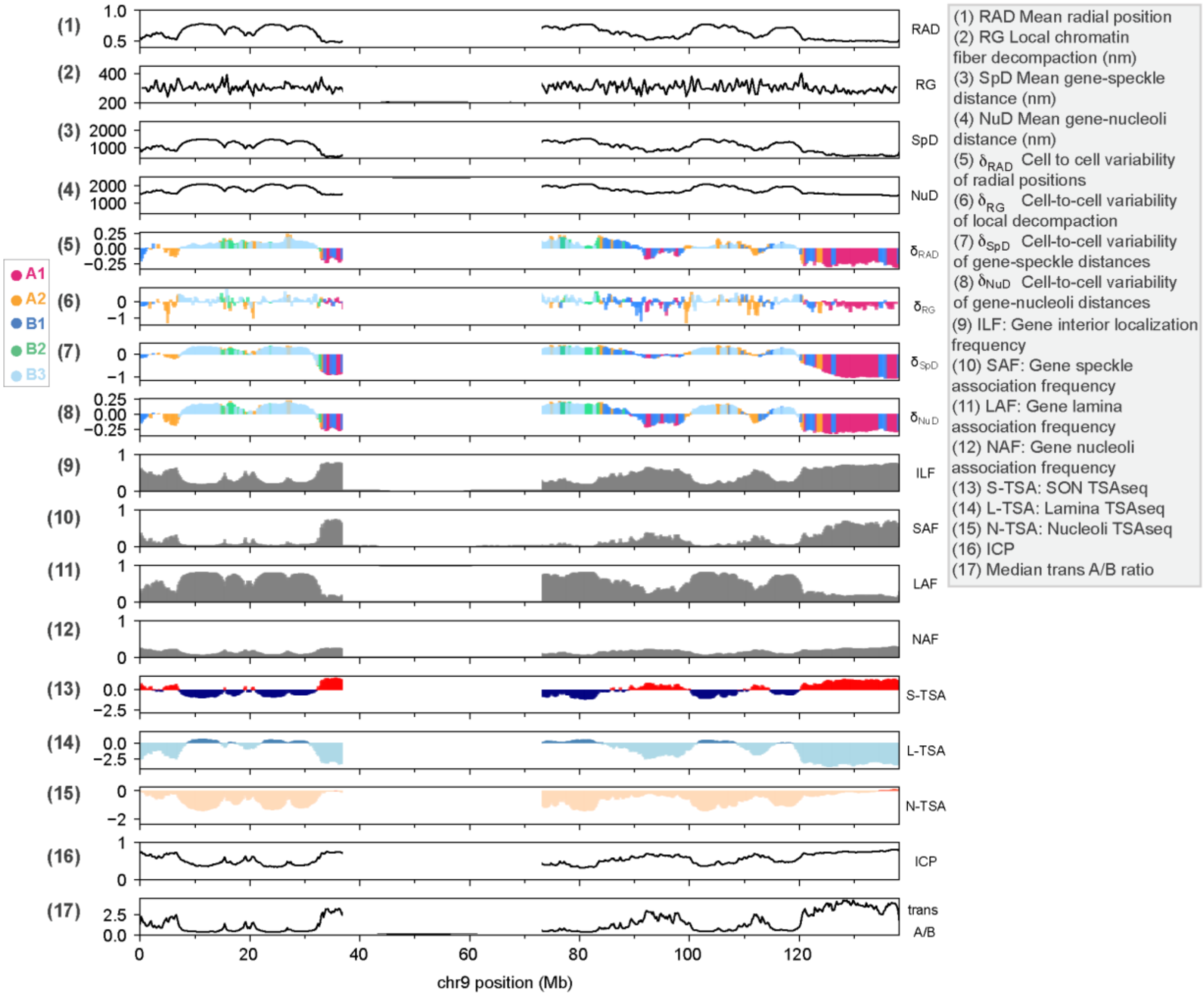


**Fig. S9.** Structure feature profiles for chromosome 9.

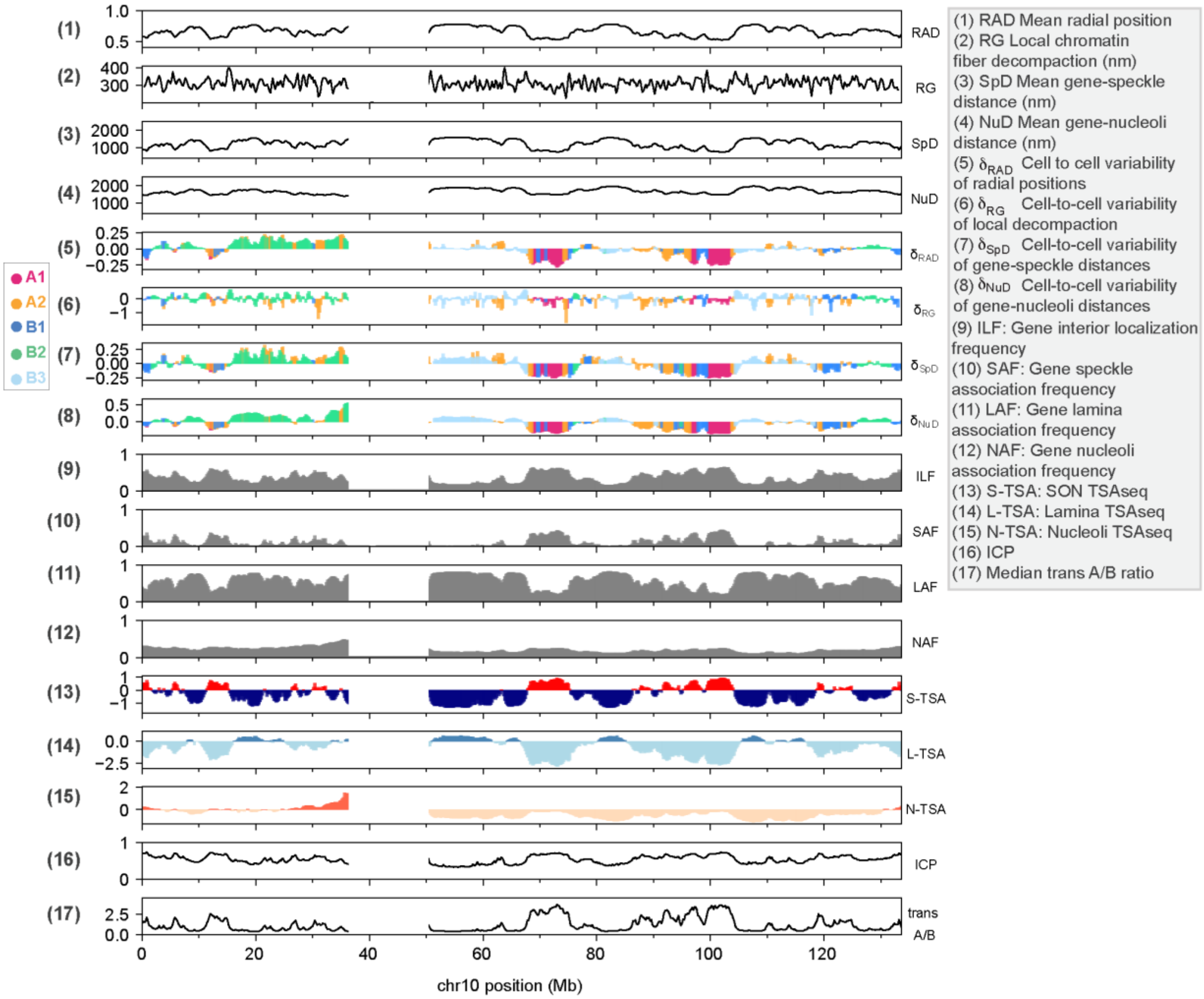


**Fig. S10.** Structure feature profiles for chromosome 10.

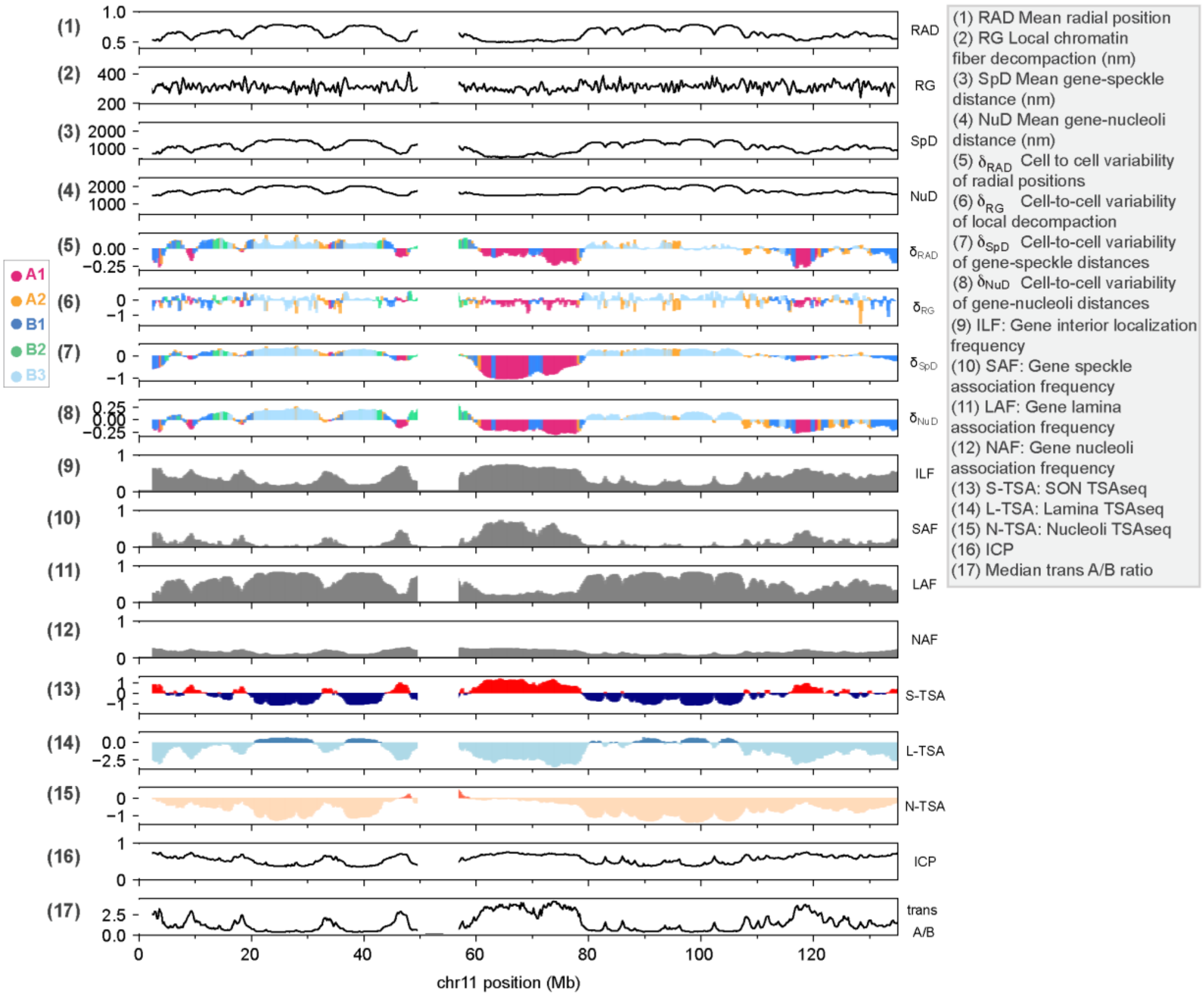


**Fig. S11.** Structure feature profiles for chromosome 11.

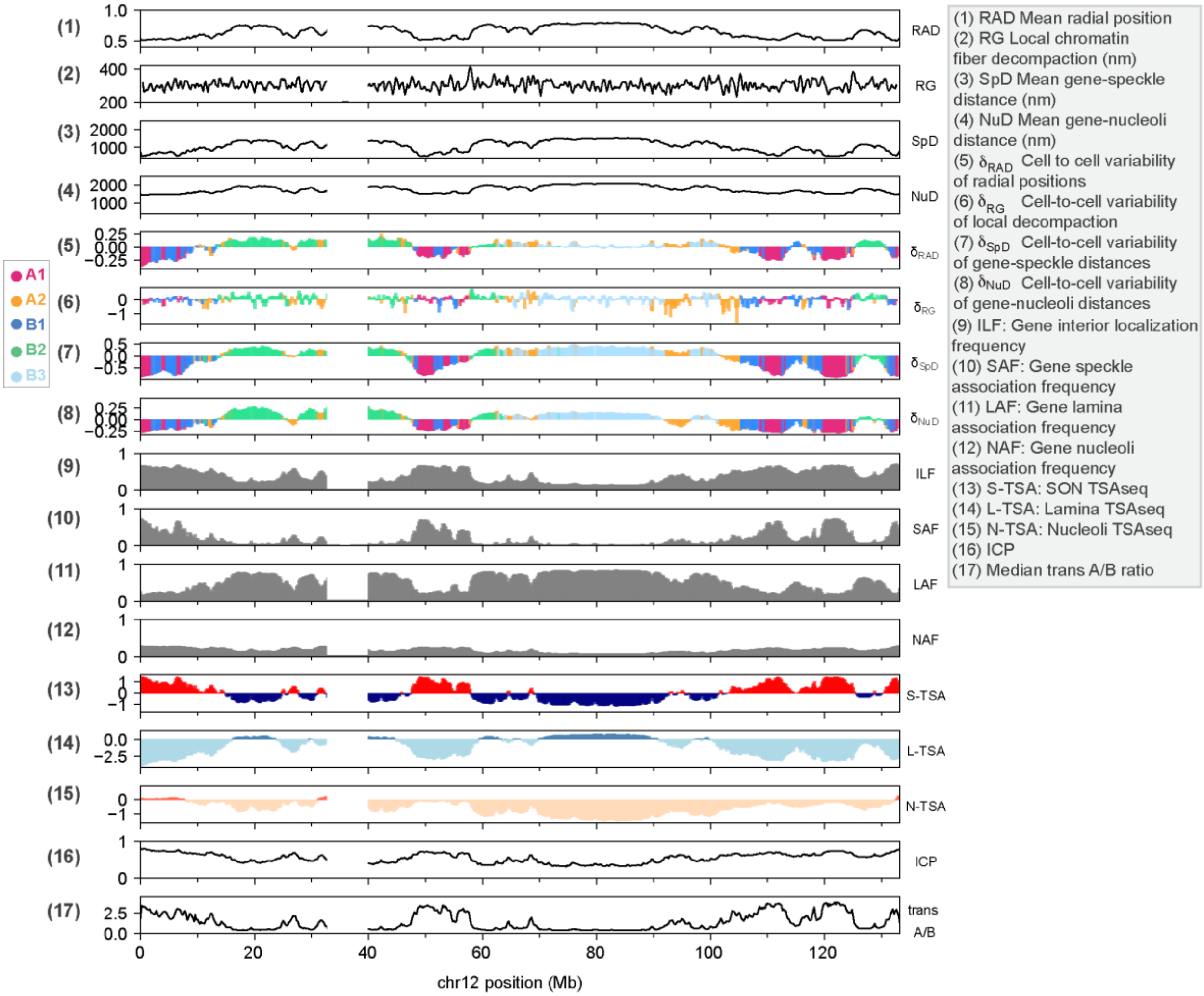


**Fig. S12.** Structure feature profiles for chromosome 12.

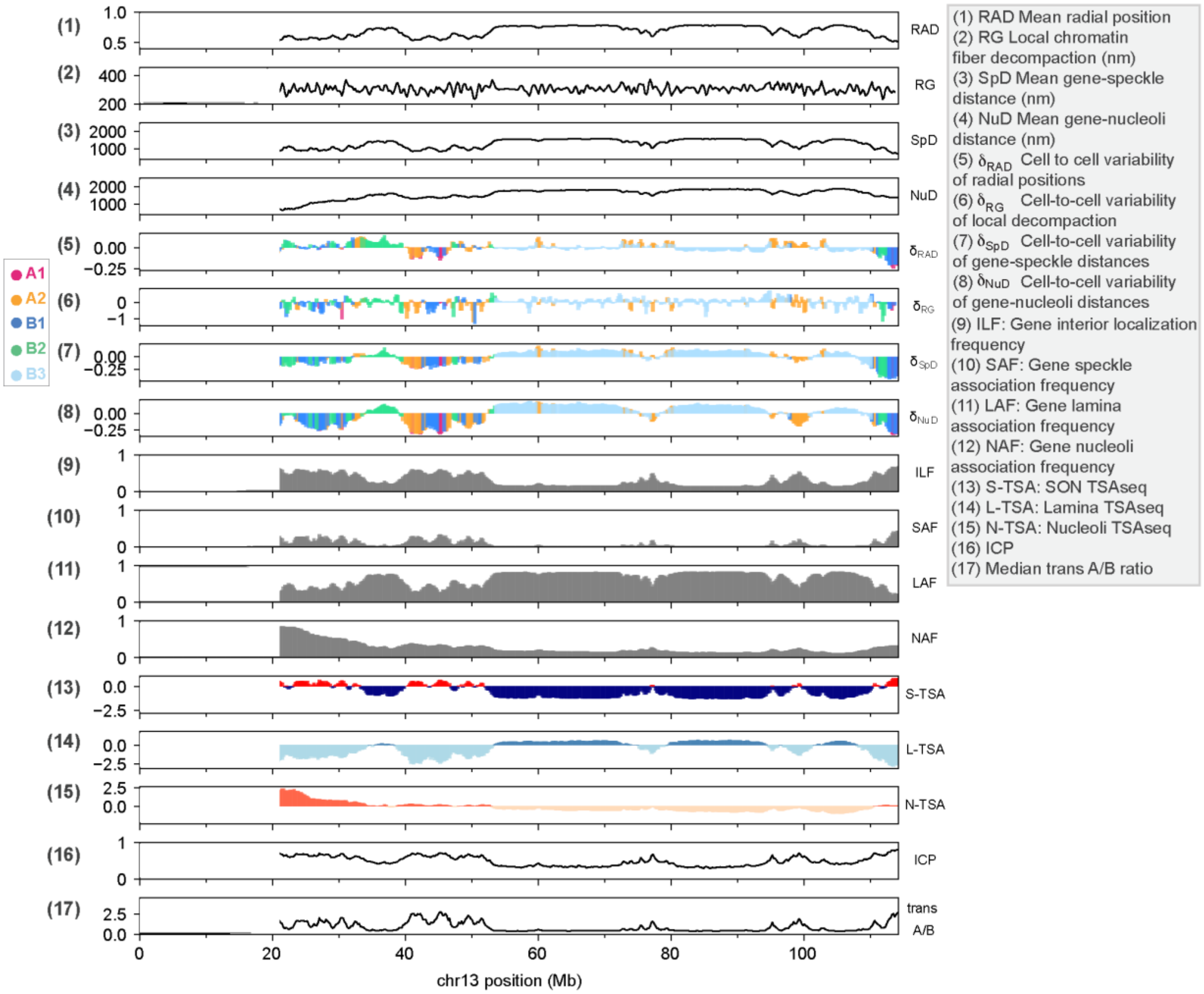


**Fig. S13.** Structure feature profiles for chromosome 13.

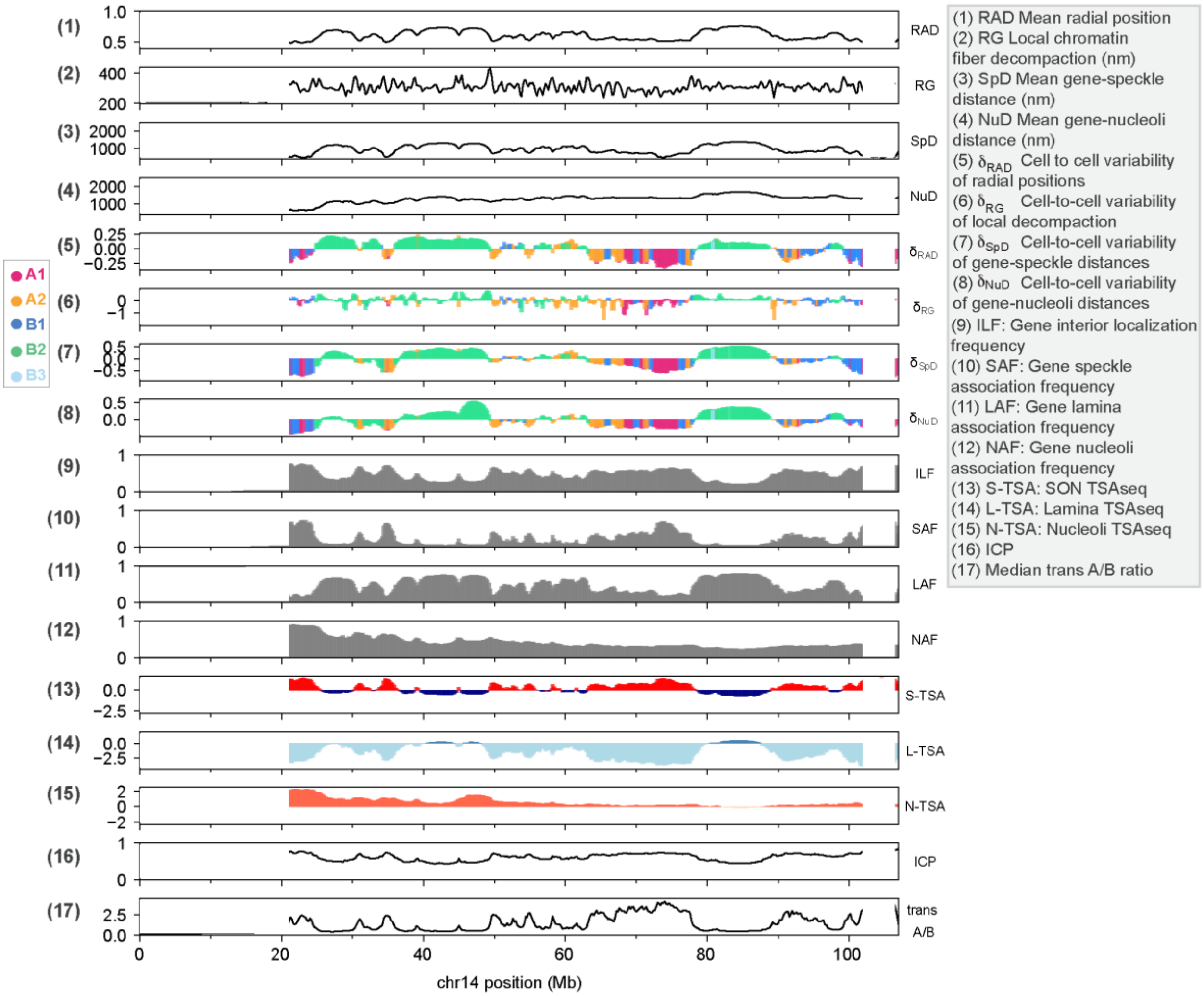


**Fig. S14.** Structure feature profiles for chromosome 14.

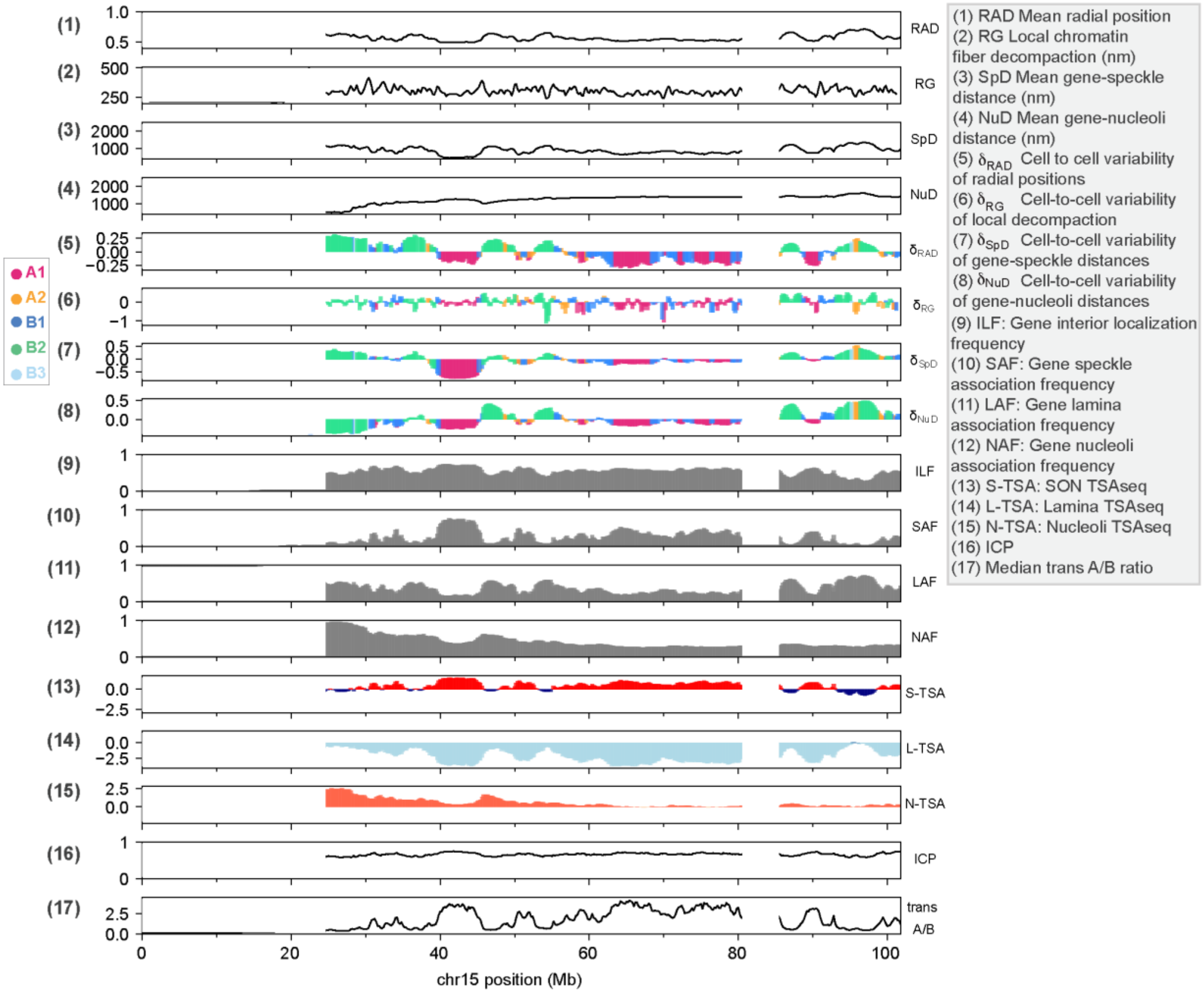


**Fig. S15.** Structure feature profiles for chromosome 15.

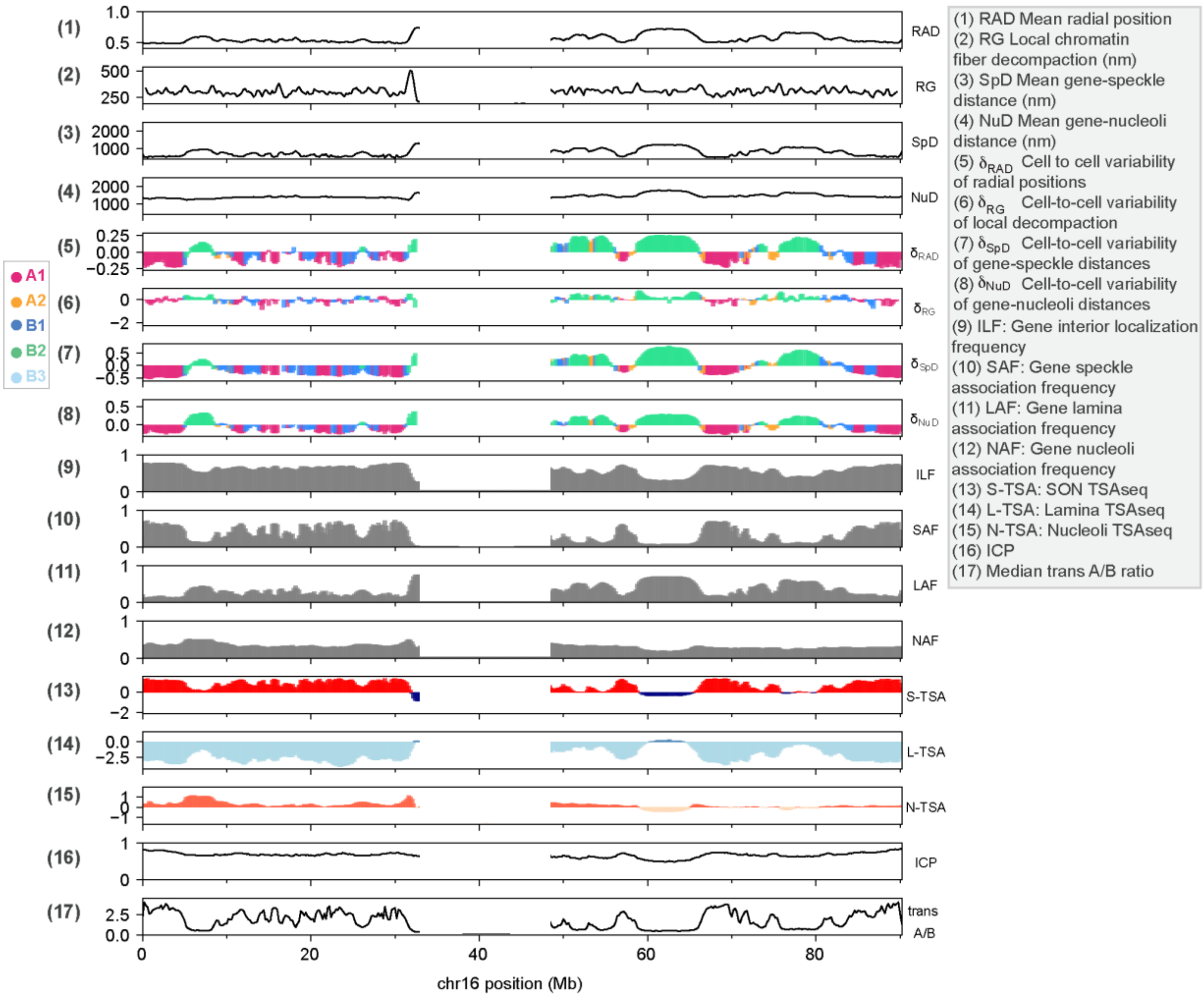


**Fig. S16.** Structure feature profiles for chromosome 16.

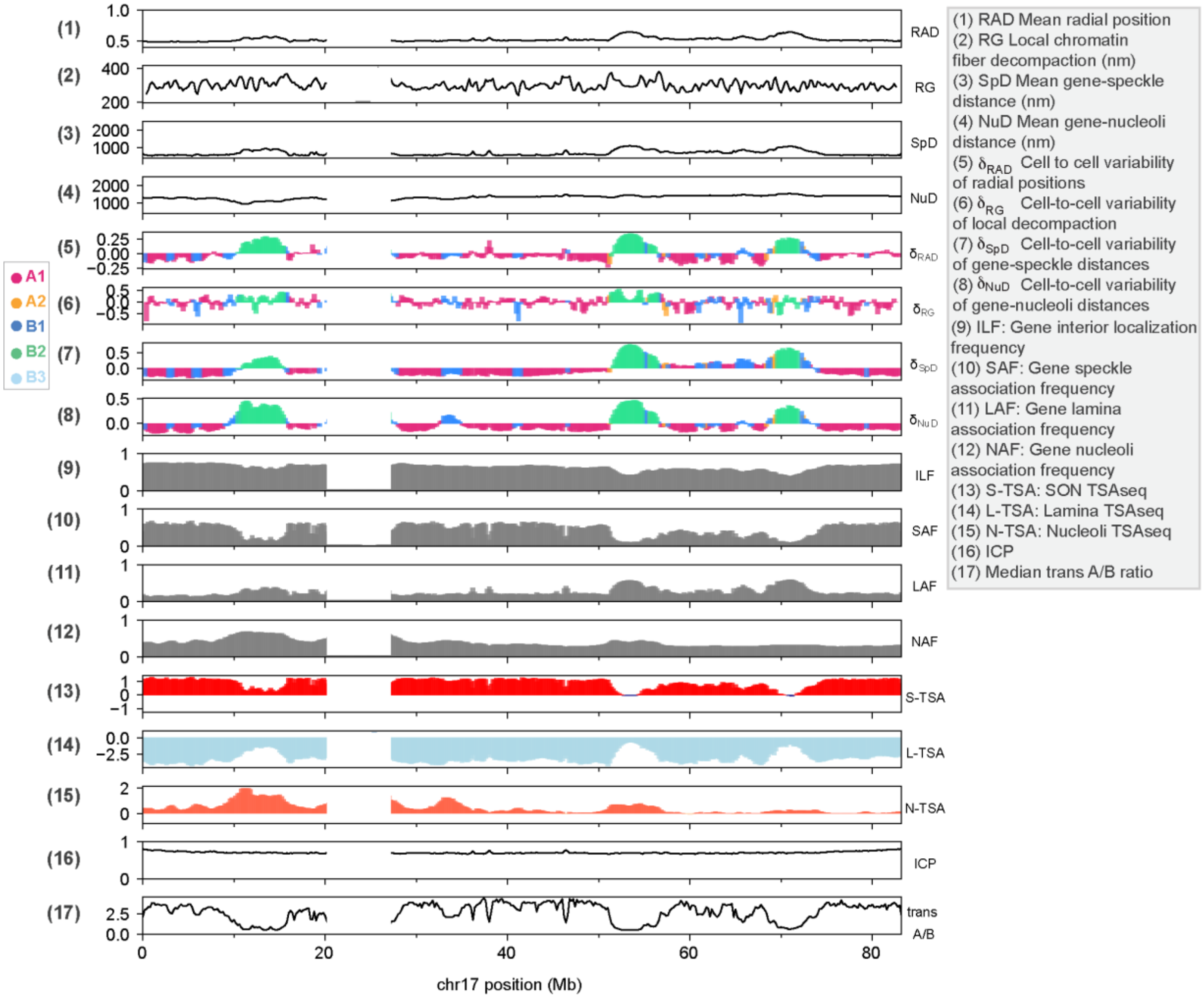


**Fig. S17.** Structure feature profiles for chromosome 17.

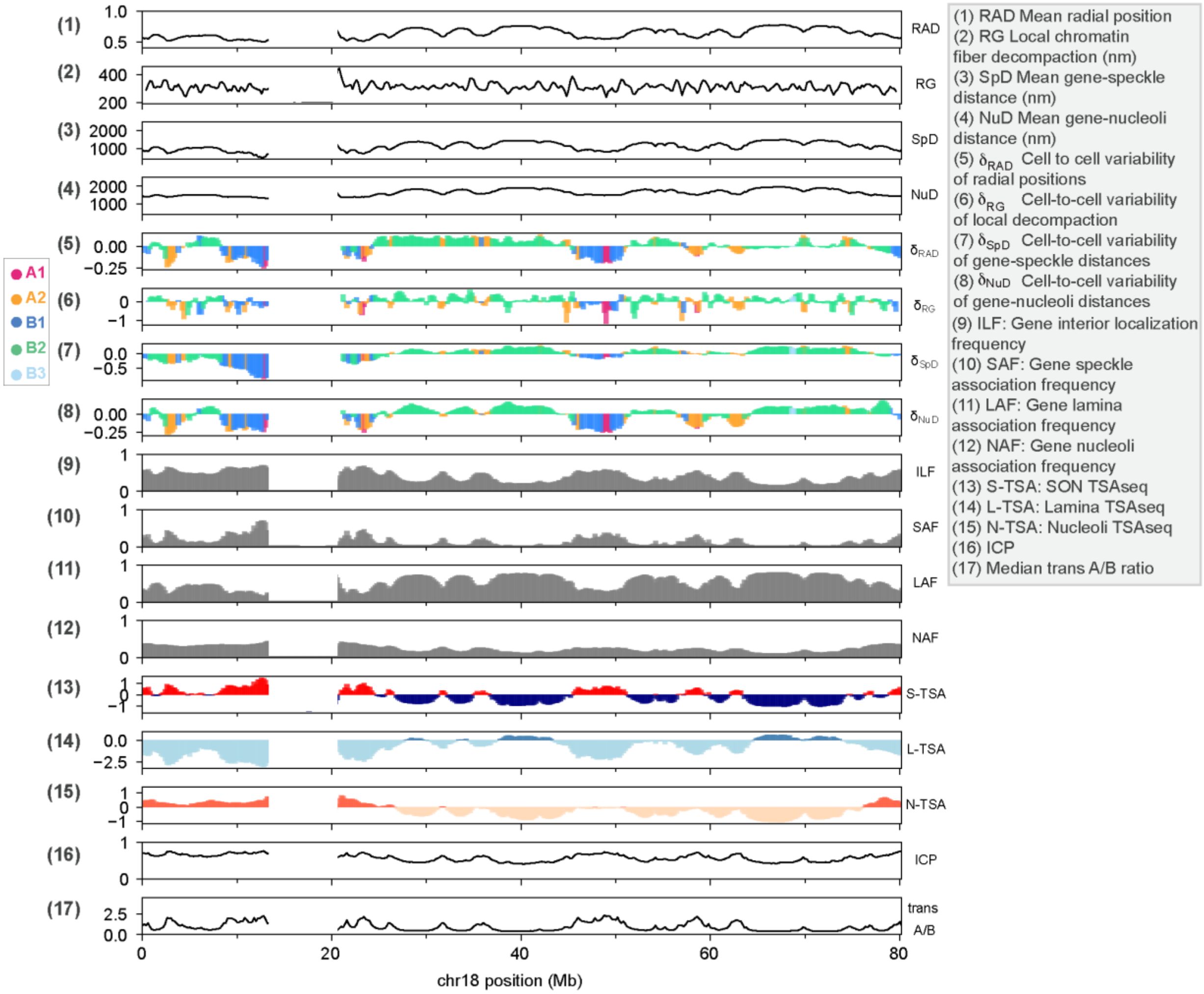


**Fig. S18.** Structure feature profiles for chromosome 18.

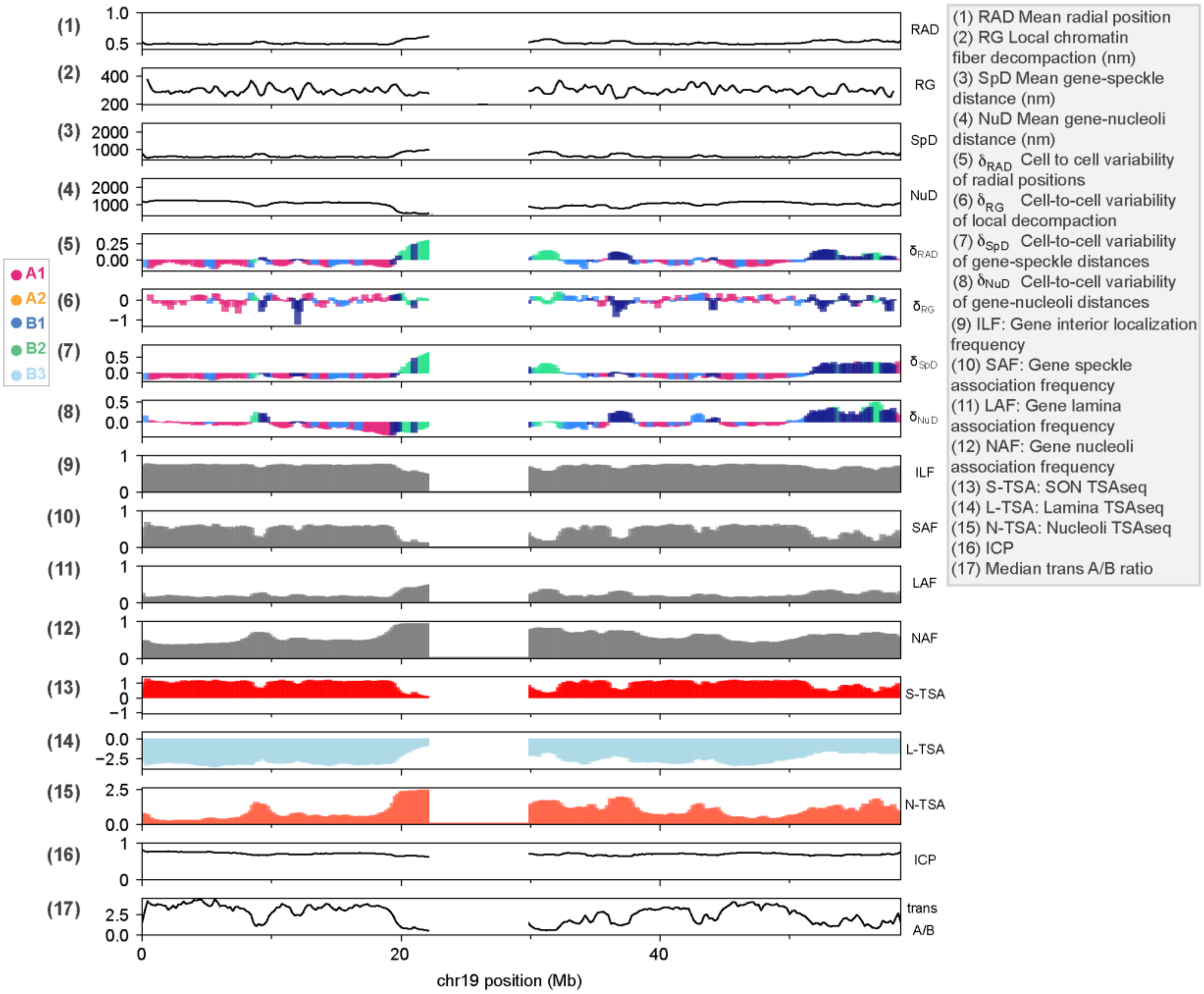


**Fig. S19.** Structure feature profiles for chromosome 19.

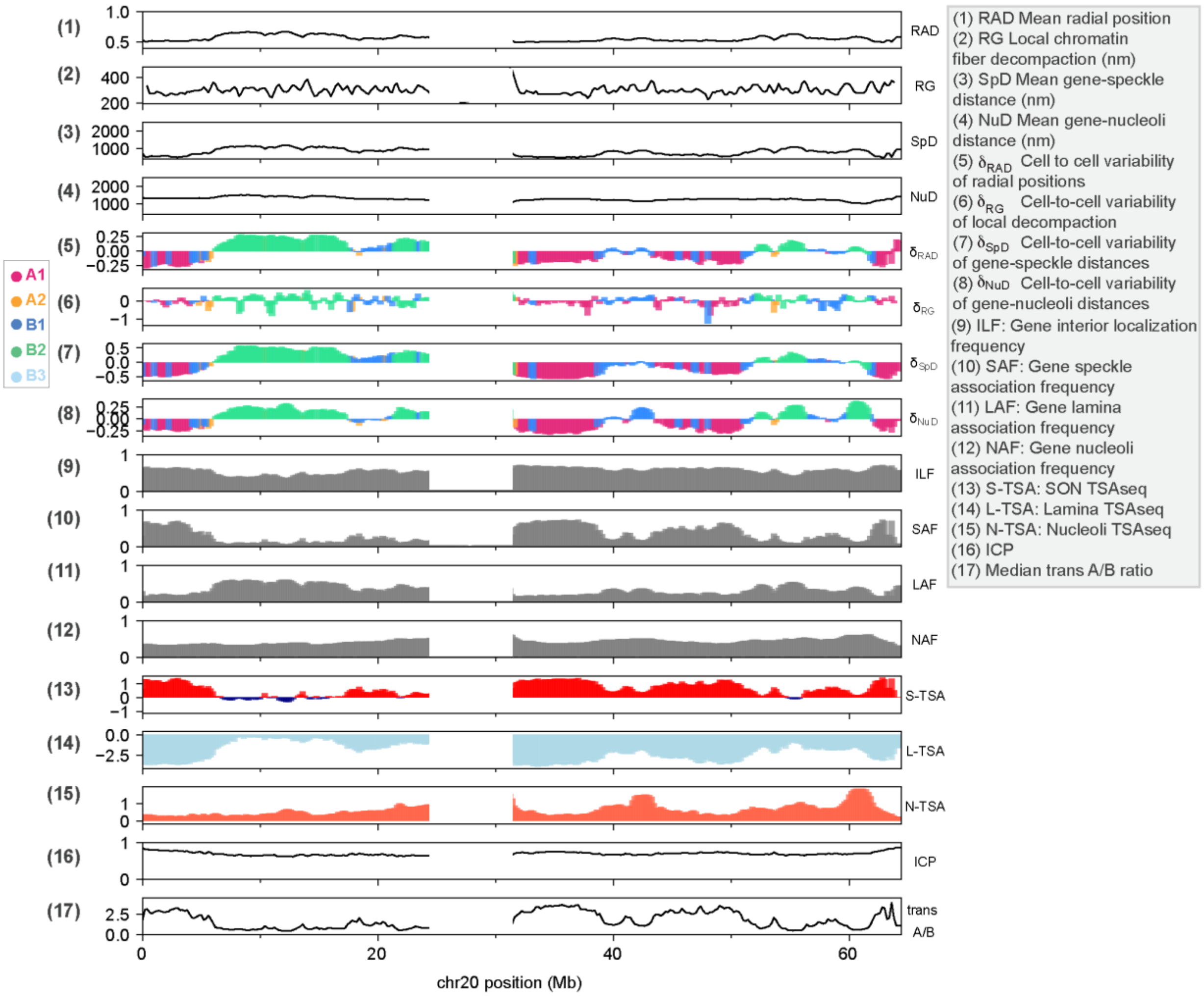


**Fig. S20.** Structure feature profiles for chromosome 20.

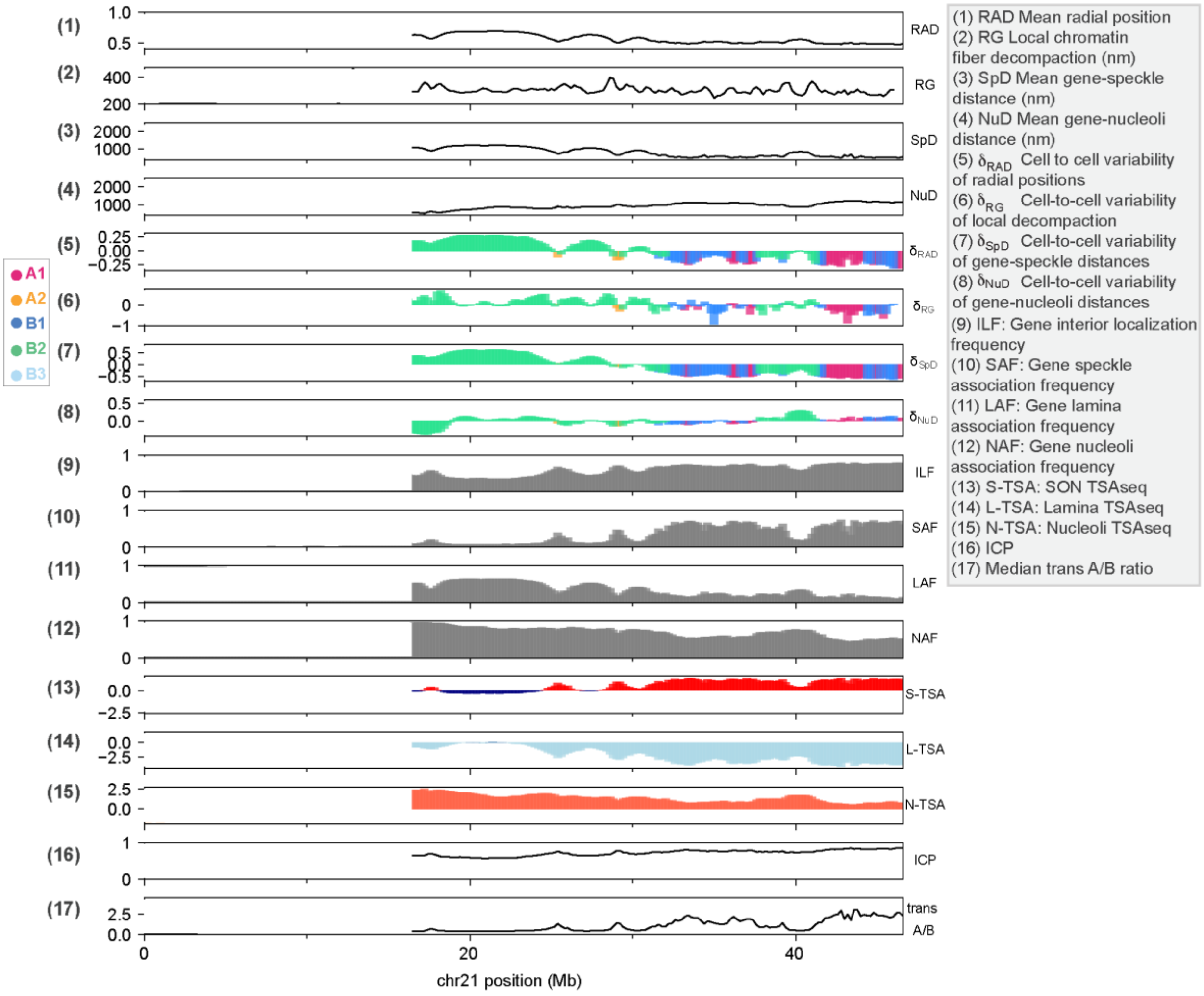


**Fig. S21.** Structure feature profiles for chromosome 21.

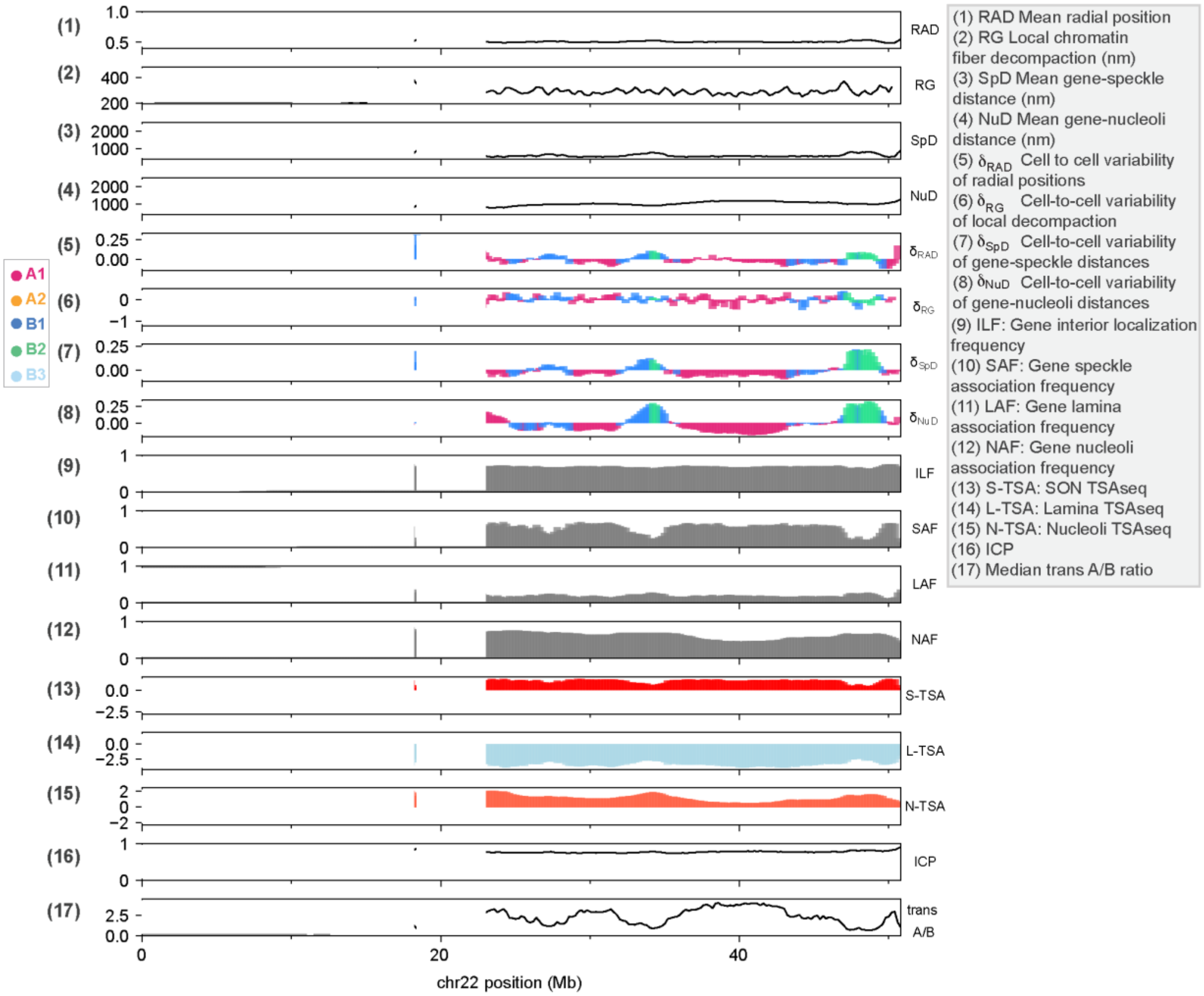


**Fig. S22.** Structure feature profiles for chromosome 22.

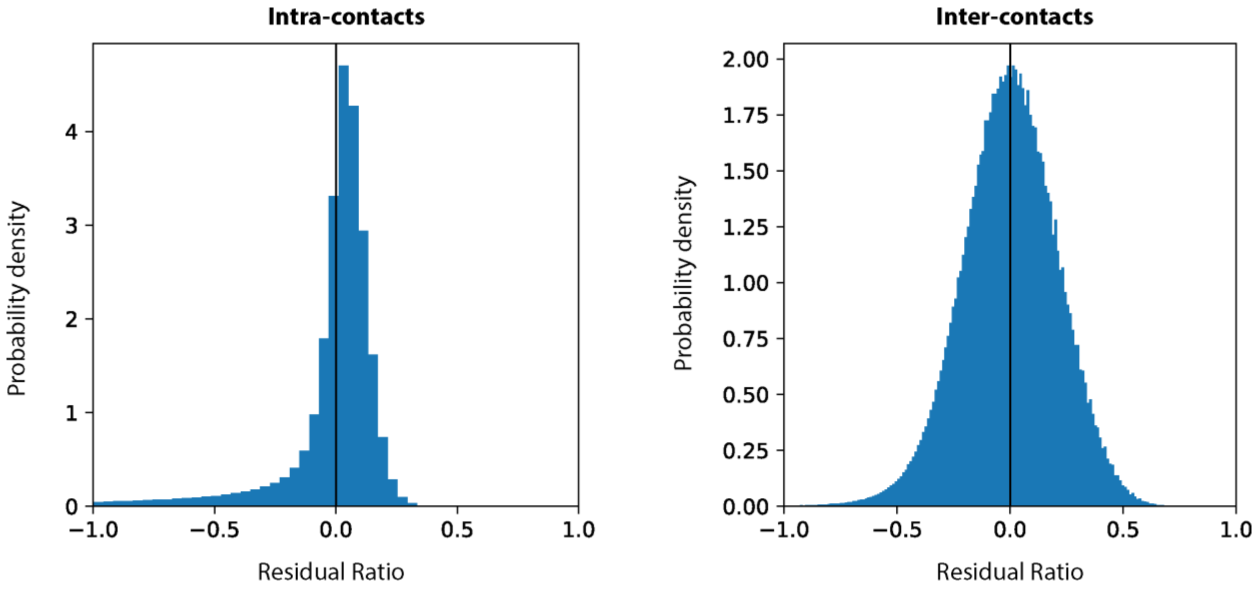


**Fig. S23.** Residual ratios. The residual ratio $\Delta r$ is defined as $\Delta r_{kl} = \left(f_{kl}^{input} - f_{kl}^{model}\right)/f_{kl}^{input}$ with $f_{kl}^{input}$ and $f_{kl}^{model}$ as the contact probabilities between regions *k* and *l* from experiment and models, respectively. Residual ratios are very small, and centered at a median of 0.03 (mean= -0.05) for intra-chromosomal (left) and 0.001 (mean = -0.002) for inter-chromosomal (right) contacts, showing excellent agreement between experiment and model.

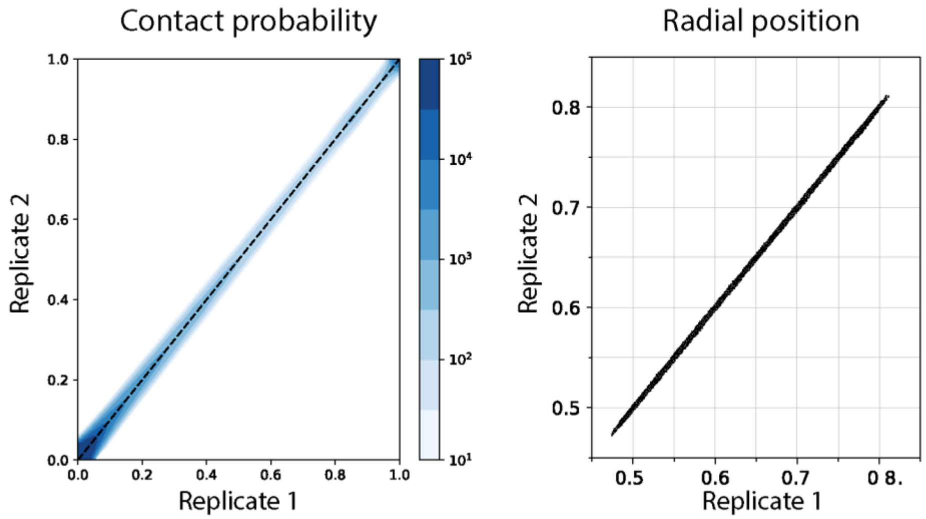


**Fig. S24.** Comparison of replicate populations. Scatter plots of contact probabilities (left, Pearson correlation: $r$ = 0.99, p=0) and radial positions (right, Pearson correlation: $r$ = 0.99, p=0) for two replicates.

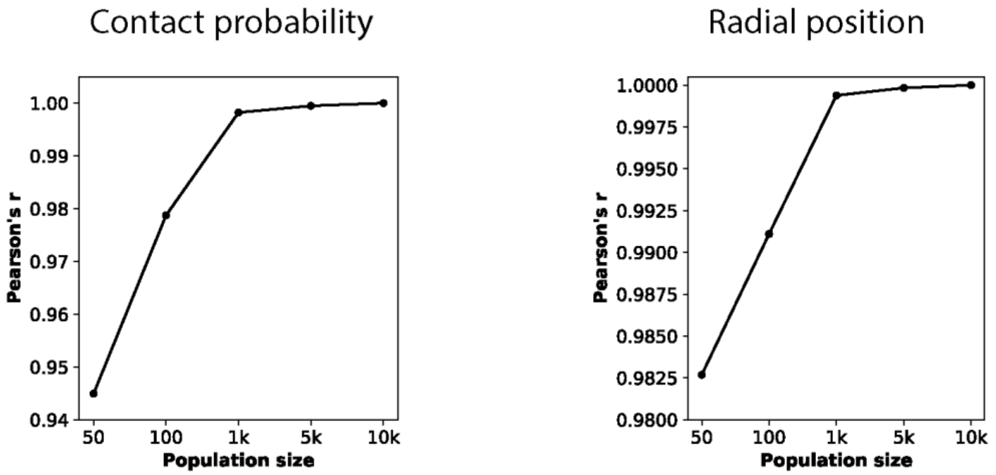


**Fig. S25.** Population size convergence plots. Pearson correlations between the population with 10,000 structures and populations with smaller sizes (50, 100, 1,000, and 5,000 structures). Contact probabilities (left) and radial positions of chromatin regions (right) already converge at 1,000 structures and have very high correlations (>0.99) with the 10,000 structure population.

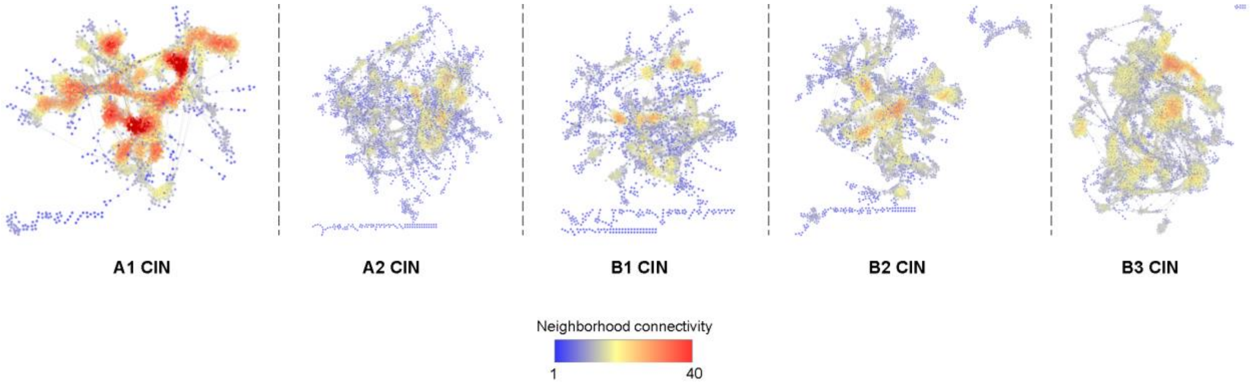


**Fig. S26.** Representative chromatin interaction networks (CIN) for chromatin in each subcompartment in a single structure. Each node in CINs represents a single chromatin region connected by edges if the two regions are in physical contact in the 3D structure. Nodes are colored by their neighborhood connectivity (i.e. the average contacts formed by their neighbor nodes) ranging from low (blue) to high (red).

**Table S1.** Pearson and stratum adjusted correlation coefficients (SCC)[1] between the input and output Hi-C matrices for each chromosome. For SCC calculation, the smoothing parameter and the upper bound of the genomic distance for interacting loci were set to 0 and 50 Mb, respectively.

| **Chromosome** | **Pearson's R** | **SCC** |
|---|---|---|
| chr1 | 0.98 | 0.83 |
| chr2 | 0.99 | 0.90 |
| chr3 | 0.99 | 0.91 |
| chr4 | 1.00 | 0.92 |
| chr5 | 0.99 | 0.90 |
| chr6 | 0.99 | 0.88 |
| chr7 | 0.99 | 0.90 |
| chr8 | 1.00 | 0.90 |
| chr9 | 0.98 | 0.84 |
| chr10 | 0.99 | 0.86 |
| chr11 | 0.99 | 0.87 |
| chr12 | 0.99 | 0.85 |
| chr13 | 0.99 | 0.94 |
| chr14 | 0.98 | 0.88 |
| chr15 | 0.97 | 0.84 |
| chr16 | 0.98 | 0.81 |
| chr17 | 0.96 | 0.78 |
| chr18 | 0.99 | 0.87 |
| chr19 | 0.98 | 0.81 |
| chr20 | 0.97 | 0.79 |
| chr21 | 0.98 | 0.88 |
| chr22 | 0.97 | 0.88 |
| chrX | 1.00 | 0.91 |

**Table S2.** Experimental data used in our analyses.

| Data | Accession Code |
|---|---|
| Hi-C[2] | GEO: GSE63525 |
| SON TSA-seq[3] | GEO: GSE81553 |
| LaminB1 TSA-seq[3] | GEO: GSE81553 |
| Single cell Lamina DamID[4] | GEO: GSE56465 |
| LaminB1 pA-DamID[5] | 4DN: 4DNFIGL8MCSJ |
| GRO-seq[6] | GEO: GSM1480326 |
| GP-seq[7] | GEO: GSE135882 |
| Repli-seq[8] | GEO: GSM923451 |
| ChIP-seq (Histone modif.)[9,10] | ENCODE: ENCFF313LYI, ENCFF171MDW, ENCFF776DPQ, ENCFF309OEW, ENCFF028KBY, ENCFF601YET, ENCFF831ZHL, ENCFF039HDL, ENCFF340JIF, ENCFF803DJF, ENCFF683HCZ |
| scRNA-seq[11] | GEO: GSM3596321 |
| Superresolution imaging[12] | https://zenodo.org/record/3928890 |
| Subcompartments[2] | GEO: GSE63525 |
| Compartments[2] | 4DN: 4DNFILYQ1PAY |
| LADs | obtained from refs.[4,7] |
| Enhancers/Superenhancers | obtained from ref.[13] |
| Genome structure population and structural features | https://doi.org/10.5281/zenodo.7352276 |

## Analyses related to structural features

Other radial position related analyses

i. *Overlap of subcompartment borders and large radial position transitions:* To identify regions coinciding with large transitions in radial positions, we first calculate each region's gradient in radial position from their average radial position profiles. Peaks and valleys in the gradient profile coincide with the regions of large radial transitions in the chromosome and are identified with the *detect_peaks* python package[14]. We obtain 1408 regions with large radial transitions with minimum peak height (mph) set to 0.01 (the gradient values range between -0.06 – 0.05.) to filter out regions with minimal radial transitions. We then check if these identified regions coincide with the subcompartment borders, i.e. where two neighboring chromatin regions are in different subcompartments. We determine an overlap if there is a subcompartment border within a 1-Mb window of a given identified region with a large radial transition.

ii. *Shell analysis:* To map the preferred positions of 200-kb regions in the nucleus, we divide the nuclear volume of each model into 5 concentric shells $L = \{L_1, L_2, L_3, L_4, L_5\}$ so that each shell contains the same amount of chromatin in each single structure. We then calculate the probability of a subcompartment $s$ to be in any shell from $L$:

$$P_{s,L_k} = \frac{1}{M}\sum_{m=1}^{M}\frac{N_{s,L_k,m}}{N_s}$$

where $N_{s,L_k,m}$ is the number of regions from subcompartment $s$ in shell $L_k$ in structure $m$, $N_s$ is the total number of regions in subcompartment $s$, and $M$ is the total number of structures.

iii. *Comparison with GPSeq:* GPSeq scores[7] are rescaled to have values between 0 – 1, where scores 0 and 1 correspond to a chromatin region being at the nuclear lamina and nuclear center, respectively[7]. Average radial positions extracted from our structures vary between 0.48 – 0.94 with higher values corresponding to proximity to nuclear lamina. For comparison with GPSeq, we subtract the average radial positions from 1 and then rescale the values to be between 0 – 1.

iv. *Average radial positions of regions from different replication phases:* Genomic regions are divided into 6 groups (G1b, S1, S2, S3, S4, G2) based on their mapped replication

phases[8]. For each group, the distribution of the average radial positions is then determined from the structure population.

Other RG related analysis

*i.* *TAD border detection:* To investigate if chromatin regions with maxima in RG profiles coincide with TAD borders, we first identify peak regions in the average RG profiles with the *detect_peaks* python package[14]. 2068 peak regions are detected genome-wide with minimum peak distance (mpd) set to 3 (peaks must be at least 3 data points/600-kb apart from each other). We then check if these identified regions coincide with TAD borders detected by TopDom[15], HiCseg[16], InsulationScore[17], and TADbit[18]. We determine an overlap if there is a TAD border within ±200-kb window of a peak region.

*ii.* *RG peak frequency:* Peak regions in the RG profiles are detected in each individual structure using *detect_peaks* python package[14] with same parameters as in the previous section. The RG peak frequency (PF) of a region $i$ is then calculated as:

$$PF_I = \frac{n_i + n_{i'}}{2M}$$

where $n_i$ and $n_{i'}$ are the number of structures in which region $i$ and its homologus copy has an RG peak, and $M$ is the number of genome structures in the population.

Other SpD, NuD related analysis

*Speckle distance heatmaps:* A speckle distance heatmap for a chromosome visualizes, for a given chromatin region, the speckle distance variability across the population of models. For each copy of a chromatin region, the distance to the nearest predicted speckle is calculated in each structure of the population. These distances (20,000 distances total due to 2 copies and 10,000 structures) are ranked from lowest to highest values and plotted along a column of the speckle distance heatmap and color coded according to the distance. Colors range from low distance (red) to large distances (blue).

Other SAF, LAF, NAF related analyses

i. *Predicting lamin B1 DamID signals using LAF:* The predicted laminaDamID signal of region $I$ is calculated as:

$$predicted\ laminaDamID\ signal_I = log_2\left(\frac{LAF_I}{\overline{LAF}}\right)$$

where $\overline{LAF}$ is the mean lamina association frequency calculated from all regions in the genome.

ii. *Comparison with imaging data:* We compare our SAF, LAF and NAF values with imaging data[12]. To calculate association frequencies from imaging and models, we use different distance thresholds (250, 500, 750, 1000 nm distance thresholds for SAF and LAF when calculated from imaging or models, and additional thresholds of 1250, 1500, 1750, 2000 nm for LAF when calculated from models) to define an association to the nuclear body of interest. We find that the best correlations are obtained when the following distance thresholds are used:

- SAF: 500 nm for imaging, 750 nm for models
- NAF: 1000 nm for imaging, 1000 nm for models
- LAF: 1000 nm for imaging, 2000 nm for models

For SAF comparisons, we use the predicted speckle partitions from interior regions (Case 2 for speckle partitions in *Identifying spatial partitions via Markov clustering*).

<u>Other TSA-seq related analysis:</u>

*i. Predicting SON TSA-seq signals using only cis relationships in folded chromosomes:* To identify contributions of cis interactions in SON TSA-seq signals, speckle locations are defined by the geometric center of consecutive A1 sequence blocks formed by more than 1 A1 chromatin region (instead of the geometric center of A1 spatial partitions, which can be formed by both cis and trans chromosomal interactions). For single A1 regions, the bead center location is used instead. For each chromatin region, we then calculate its spatial distances to these predicted speckle locations in the folded chromosome, which are used to predict the resulting TSA-seq signals from cis interactions only.

ii. *Predicting SON TSA-seq signals using only cis relationships in random conformations*: We also repeat the same calculations as defined in the previous section, but instead of the folded chromosomes, use models with random chain configurations, generated without Hi-C data (i.e. only chain connectivity and excluded volume). TSA-seq data is calculated accordingly from the corresponding distances based on the random polymer chain configurations.

iii. *Predicting SON TSA-seq signals using speckle distances based on A1 sequence locations:*

Speckle locations are approximated by the sequence positions of A1 regions, either as median sequence position for a block of consecutive A1 chromatin regions or the sequence positions of individual A1 regions, if their neighboring regions are not part of the A1 subcompartment. The distance $d_{ij}^{seq}$ between a chromatin region *i* and speckle position *j,* separated in sequence by *n* chromatin regions, is then defined as $d_{ij}^{seq} = 2n \times R^{ex}$ , where $R^{ex} = 118\ nm$ is the excluded volume radius of a chromatin region in the models (see *Genome representation*). These distances are then used to predict SON TSA-seq signals as defined above.

iv. *Histone modification histograms based on predicted SON TSA-seq deciles:*

Following the procedure described in ref[3], we divide the 200-kb chromatin regions in our models into 10 decile groups based on their predicted SON TSA-seq signals; deciles 1 and 10 contain regions with the lowest and highest 10% predicted TSA-seq signals, respectively. We then count the number of mapped peaks of H3K27me3, H3K4me3, and H3K9ac as well as the number of A1, A2, A1+A2 regions in each decile, and calculate the fraction of histone modification peaks or A1/A2 regions accrued in each decile. For mapping histone modification peaks to 200-kb bins to match our models' resolution, see *Mapping experimental data to models* in *Supplementary Information*. Same histograms using experimental TSA-seq deciles are re-generated from Fig. 8 in ref[3] using WebPlotDigitizer[19].

## Comparison of gene expression with structural features

- **Transcription frequency**

Transcription frequency (TRF) of each gene in the single cell RNA-seq (scRNA-seq) data is defined as the fraction of cells in the population of cells, where the gene has non-zero mRNA transcription counts in the scRNA-seq data[11]. TRF is also calculated from the recently published nascent RNA-MERFISH imaging data as the fraction of cells where the gene is transcribed (transcription: on) in the population of imaged cells[12].

- **Gene expression heatmaps**

Gene expression heatmaps for each chromosome visualize the variability of mRNA counts (the expression levels) for each gene in a population of cells[11]. For each chromatin region, the observed mRNA count in each cell of the population of models is ranked from highest to lowest values and plotted along a column. Colors ranged from high mRNA counts (red) to 0 (dark blue).

- **ROC curve for assessing performance to classify lowly or highly expressed genes**

We first identify the top 10% (T10) and the bottom 10% (B10) genes with the highest and the lowest total non-zero mRNA counts (i.e. gene expression values) in the scRNA-seq data[11]. Several structural features (mean radial positions, ILF, mean speckle distances, SAF, variability of radial positions and speckle distances) are then calculated for all chromatin regions mapped to T10 genes and B10 genes.

To determine the most informative structural features for distinguishing T10 genes from B10 genes, we perform receiver operator characteristic (ROC) analysis. Specifically, for each structural feature, we define 10 threshold levels, equally separating the range of values for each structural feature.  Then we determine how well the gene in the T10 and B10 groups are separated by each threshold value by calculating the corresponding number of true positives/negatives (TP, TN) and false positive/negatives (FP, FN).

For each structural featue $f$ and for each threshold level, $t,$ the true positive rate (TPR) and false positive rates (FPR) are then calculated as

$$TPR_{t,f} = \frac{TP}{TP + FN}$$

$$FPR_{t,f} = 1 - \frac{TN}{FP + TN}$$

The ROC curves are then plotted for each feature using TPR/FPR values.

## Other structural analyses

- **Experimental GRO-seq and TSA-seq data analysis**

Averaging TSA-seq and GRO-seq signals in concentric shells around subcompartment partitions:

To quantify average TSA-seq[3] and GRO-seq[6] signals for chromatin with respect to the distance to spatial partition centers of each subcompartment, the nuclear volume around a spatial partition center is divided into concentric shells, with each consecutive shell radius increasing by 200 nm. The signals are then averaged over concentric shells around partition centers as follows: In each individual genome structure, the signals of chromatin located in the same shell volume is averaged, irrespective of the chromatin's

subcompartment assignment. The average signal per shell are further averaged over all partition centers in the same subcompartment and over all structures of the population. Note that this measure only relies on the geometric position of a partition center and the folded genome (i.e. calculates average gene expression from all chromatin in a shell, independent of subcompartment annotations).

- **Neighborhood composition**

The neighborhood composition (NeC) shows how frequent chromatin regions in different subcompartments are in spatial proximity to regions of a specific subcompartment. The average percentage of subcompartment $Q$ in the neighborhood composition of subcompartment $S$ in the population is calculated as:

$$NeC_{SQ} = \frac{1}{MN_S}\sum_{m=1}^{M}\sum_{j=1}^{N_S}\frac{n_{Q,m,j}}{|N_{m,i}|}x100$$

where $M$ is the number of structures in the population, $N_S$ is the number of 200-kb regions belonging to subcompartment $S$, $\{N_{m,i}\}$ is the set of 200-kb chromatin regions in the neighborhood of the region $i$ in structure $m$, and $n_{Q,m,i}$ is the number of chromatin regions from subcompartment $Q$ in the set $\{N_{m,i}\}$. We define the neighborhood of $i$ in structure $m$ as $N_{m,i} = \{j: j \neq i, d_{ij} < 500\ nm\}$, which contains the list of all chromatin regions with less than 500 nm center-to-center distance ($d_{ij}$) to chromatin region $i$.

The neighborhood composition enrichment (NeCE) of subcompartment $Q$ in the neighborhood of subcompartment $S$ is calculated as:

$$NeCE_{SQ} = \frac{NeC_{SQ}}{\frac{1}{5}\sum_{T\in\{A1,A2,B1,B2,B3\}} NeC_{TQ}}$$

where $NeC_{SQ}$ is the neighborhood composition percentage calculated for subcompartment $Q$ in the neighborhood of subcompartment $S$ and the denominator is the average percentage of subcompartment $Q$ observed in the neighborhood of all subcompartments. Values greater than 1 ($NeCE_{SQ} > 1$) indicate that subcompartment $Q$ is enriched in the neighborhood of subcompartment $S$, whereas values lower than 1($NeCE_{SQ} < 1$) show depletion of $Q$ around $S$.

- **Enrichment heatmaps for various features**

Enrichment of structural features, experimental TSA-seq, DamID, and GRO-seq signals, and histone modifications in various groups:

To identify structural feature or experimental signal enrichments for chromatin in different groups (subcompartments, TSA-seq deciles, superenhancers, enhancers, replication phases, A/B-LV/HV groups, and T10/B10 genes), we first normalize each feature value to range between 0 and 1. We then calculate the enrichment of a structural feature $f$, for group $g$ as:

$$enrichment_{g,f} = log_2 \frac{\frac{1}{N_g} \sum_{c=1}^{N_g} f_c}{\bar{f_r}}$$

where $N_g$ is the number of 200-kb chromatin regions in group $g$, $f_c$ is the structure feature value for chromatin region $c$. For $\bar{f_r}$, we first randomly select the same number ($N_g$) of regions in the genome and calculate the average feature value, and repeat this step 1000 times. For the enrichment of histone modifications in A-LV and A-HV groups, we randomly select the same number of regions only from regions in compartment A. We then take the average of 1000 different average feature values calculated from randomly selected regions.

For visualization purposes, we reverse the ranges of radial positions, mean-speckle, and mean-nucleoli distances in the structural feature enrichment heatmaps, so lower values would be indicated with red.

Enrichment of replication phases, LADs, and subcompartments in A-LV, A-HV, B-LV, and B-HV groups:

We calculate the enrichment of various tags $t$ (based on replication phases, LADs, or subcompartments), in group $g$ as:

$$enrichment_{g,t} = log_2 \frac{fr_{g,t}}{\overline{fr_{r,t}}}$$

where $fr_{g,t}$ is the fraction of regions with tag *t* in group *g*. For $\overline{fr_{r,t}}$, we first randomly select the same number of genomic regions (as in group *g*) and calculate the fraction of regions with tag *t* among those regions, and repeat this step 1000 times. We then take the average of 1000 different fraction values.

- **K-means clustering of A and B compartments**

For clustering, we first normalize all 17 structural features using $\log_2$-transformation. We then perform K-means clustering using all transformed features for A and B subcompartments separately. We use scikit-learn python package to perform K-means clustering[20] and set the *n_clusters* parameter to 2 for A and 3 for B compartments. Clusters are then compared with actual subcompartment assignments to compute clustering accuracy. The highest prediction accuracies are obtained when clustering is performed with a subset of structural features for both A and B subcompartments. The used features in the clustering are cell-to-cell variability of radial positions, SAF, NAF, median trans A/B ratios for A, and cell-to-cell variability of radial positions and nucleoli distances, nucleoli TSA-seq, ICP, median trans A/B ratios for B subcompartment predictions, respectively.

- **Comparison with 3D in situ hybridization (3D-FISH) data**

FISH probes are mapped to 200-kb chromatin regions in our models according to the highest overlap. Radial positions and pairwise distances for each mapped probe are determined in each structure in the population and compared to the radial positions and pair distances in FISH experiments. FISH and model radial positions are normalized by their maximum values. Intra-chromosomal distances in models are defined by their surface-to-surface distances of the corresponding probe regions (in both copies of the chromosome). Colocalization fraction of inter-chromosomal pairs are calculated as following: first the center-to-center distances of all possible probe pairs ($i - j,\ i - j',\ i' - j,\ i' - j'$ where $i'$ and $j'$ are the homologous copies of each 200-kb chromatin regions, $i$ and $j$) are calculated in each structure. The minimum distance from all possible pairs in each structure is then used to calculate the fraction of models in which both regions are colocalized. We assume a loci pair is colocalized in a structure if the calculated minimum distance in that structure is lower than 1 μm ($d_{min} < 1\ \mu m$).

- **Radial positions of trans and cis interactions**

We select 1,000 random structures from the population and identify all the trans and cis chromatin interactions. Then we calculate the average radial position of the location where the trans or cis interaction occurs by taking the mean of the radial positions of the two loci forming the interaction in that structure.

## Preprocessing Hi-C data

We used *in situ* Hi-C datasets from human lymphoblastoid cell line GM12878 (reference genome hg38)[2]. For this dataset, the raw contact map was downloaded from Gene Expression Omnibus (GEO) under the accession number GSE63525. Similar to the protocol by ref.[21], low bin sequence coverage 3% regions were discarded during the normalization process. For data normalization, we adopted the same KR normalization method used in[2], leading to a normalized contact frequency matrix $\boldsymbol{F} = (f_{ij})_{K\times K}$ at 20 kb resolution with $K = 151{,}561$ bins. We then generated a probability matrix at 200 kb level as our input for our algorithm using the following approach:

We converted the contact frequencies in the 20-kb matrix to contact probabilities by scaling the frequencies by a normalization factor, $f^{max}$, which is chosen to represent the contact frequency value at which two domains have a 100% probability to form a contact. The 20-kb contact probability matrix $\boldsymbol{P} = (p_{ij})_{K\times K}$ was calculated as $p_{ij} = \min\left(\frac{f_{ij}}{f^{max}}, 1\right)$, where $p_{ij}$ and $f_{ij}$ are the contact probability and frequency values, respectively. We set the value of $f^{max}$ so that the average contact probability sum of a region is ~24, which, based on our experience, is the average number of contacts a domain has at saturation (where no more contact restraints can be satisfied, given the contact distance cutoff)[22].

We then defined a mapping $b(i)$ as the set of all 20-kb bins in matrix $\boldsymbol{P}$ that belongs to 200-kb region $i$. Then the domain-level matrix $A = (a_{ij})_{N\times N}$ was calculated as:

$$a_{ij} = mean(\ top10\% < \{p_{\alpha\beta}\colon\ \alpha \in b(i), \beta \in b(j)\} > )$$

In the case that some contacts were drastically higher than the surrounding contacts, these contacts were identified as outliers by $\{p: p > \mu + 1.5IQR\}$, where $p \in \{p_{\alpha\beta}: \alpha \in b(i), \beta \in b(j)\}$ and $\mu = mean\{p_{\alpha\beta}: \alpha \in b(i), \beta \in b(j)\}$. The IQR refers to the interquartile range of $\{p_{\alpha\beta}\}$. These outliers were excluded from the calculation.

After obtaining the contact probability matrix at 200-kb resolution, we identified bins that have spurious inter-chromosomal interaction probabilities (higher than 0.2), and removed the corresponding bins in the 20-kb raw matrix, and repeated the KR normalization, and regenerated the 200-kb contact probability matrix where no inter-chromosomal contact probability is higher than 0.2 following the same procedure explained above. Finally, we set contact probabilities between the consecutive domains as well as between domains up to 1 Mb distance in the gap regions to 1 in order to maintain the chain integrity.

## Iterative refinement of restraint assignment

During the optimization procedure, it is possible that excess contacts can lead to compact structures that cannot be further relaxed easily. A heuristic method has been put in place to compensate for such an effect, by allowing us to assess the actual portion of expected contacts that are available for allocation, which then affects the way the activation distance $d_{act}^{IJ}$ is computed in the Hi-C assignment steps (A). The empirical procedure relies on the predicament that when a population expresses more contacts than it should, we reduce the assignment probability; a lower probability (with fewer restraints) should be equally effective.

Assume the expected number of contacts $Sp_{ij}^{input}$ is the sum of a number of effective contacts that are actually imposed, $N_{eff}$, and a number of incidental contacts (that are also expressed but not imposed), $N_{inc}$:

$$Sp_{ij}^{input} = N_{eff} + N_{inc} = N_{eff} + \eta\left(S - N_{eff}\right)$$

The number of incidental contacts is expressed as a fraction of the number of non-applied (non-enforced) contacts. The latter term can originate from cooperative effects, which automatically bring loci closer without an explicit bonding term operating. We can solve for probability $p_0 = N_{eff}/S$: $p_0 = \frac{p^{input} - \eta}{1-\eta}$. This is an effective probability which controls the number of restraints to be enforced. The estimate for the scalar $\eta$ is calculated as follows.

Let us compare the factual number of contacts in the population (expressed by the tensor $A_{ij}^{X} = \sum_{s=1}^{S} \boldsymbol{W}_{ijs}^{(k-1),\mathrm{X}}$) with the predicted number of contacts from the previous assignment step ($A_{ij}^{assig} = \sum_{s=1}^{S} \boldsymbol{W}_{ijs}^{(k-1),\mathrm{assig}}$):

$$\sum_{s=1}^{S} \boldsymbol{W}_{ijs}^{(k-1),\mathrm{X}} = \sum_{s=1}^{S} \boldsymbol{W}_{ijs}^{(k-1),\mathrm{assig}} + \left( S - \sum_{s-1}^{S} \boldsymbol{W}_{ijs}^{(k-1),\mathrm{assig}} \right) \eta_{ij}$$

We can solve for $\eta_{ij}$:

$$\eta_{ij} = \frac{\sum_{s=1}^{S} \boldsymbol{W}_{ijs}^{(k-1),\mathrm{X}} - \sum_{s=1}^{S} \boldsymbol{W}_{ijs}^{(k-1),\mathrm{X}}}{S - \sum_{s=1}^{S} \boldsymbol{W}_{ijs}^{(k-1),\mathrm{assig}}} = \frac{A_{ij}^{X} - A_{ij}^{assig}}{1 - A_{ij}^{assig}},$$

which can then be plugged into the equation 1 to find a corrected assignment probability $p_0 = \frac{p^{input} - \eta}{1 - \eta}$, which is then used to update the activation distance $d_{ij}^{act}$. Please note that the correction is only implemented if there is a contact excess.

## Mapping experimental data to 200kb models

The list of experimental data used in our analyses is reported in Table S2. Each of the experimental data is in a different resolution; therefore, we performed several mapping methods to bring the data to the models' resolution (200-kb). We also lifted any hg19 data to hg38 by using the liftOver tool from UCSC[23]. The mapping methods for different data are listed below.

- **scRNA-seq**

Each gene in the scRNA-seq data set[11] was mapped to the 200-kb chromatin region in our models with the largest sequence overlap. Each gene was mapped to a single chromatin region, and if multiple genes were mapped to a single chromatin region, the gene with the highest total expression level was selected. After the mapping process, a total of 8,920 genes (7,090 of them with non-zero total expression levels) were mapped to 8,920 200-kb chromatin regions in our models. In our analyses, scRNA-seq data for the GM12878 cells in G1 phase were used.

- **SON and LaminB1 TSA-seq**

Each region in the TSA-seq data[3] was mapped to 200-kb regions with an overlap of 50% or higher. After mapping, each 200-kb region had multiple TSA-seq regions; therefore the signals mapped to each 200-kb region were averaged (we first took the inverse log2 of the signals, then averaged and took the log2 of the average value.). Transcription hot zones were also downloaded from ref[3].

- **LaminB1 scDamID and pA-DamID**

Each region in the pA-DamID data[5] was mapped to 200-kb regions with an overlap of 50% or higher. After mapping, each 200-kb region had multiple DamID regions; therefore the signals mapped to each 200-kb region were averaged (we first took the inverse log2 of the signals, then averaged and took the log2 of the average value.).

100-kb single-cell LaminaDamID lamina contact frequencies[4] were mapped to 200kb and averaged over 200-kb regions.

- **GP-seq**

First, 100-kb rescaled GP-seq scores were averaged over 4 replicate experiments (2 HindII, 2 MboI experiments) for each 100-kb region[7]. The values were then mapped to 200-kb and averaged over 200-kb regions.

- **GRO-seq**

GRO-seq read counts[6] at plus and minus strands were summed up for each 200-kb region.

- **Repli-seq**

We first calculated the average percentage normalized signals (percentage of normalized tag densities over all phases) in each 200-kb region for each phase (G1b, S1, S2, S3, S4, G2)[8]. We then assigned each 200-kb region with the cell phase with the highest average percentage. For example, if a 200-kb region showed the average normalized percentage of tag densities for G1b, S1, S2, S3, S4, and G2 as 10%, 45%, 20%, 10%, 5%, and 0%, then the region was assigned to S1 phase.

- **Superresolution imaging**

For the recently publised superresolution imaging data[12], we used the provided datasets at https://zenodo.org/record/3928890. 1041 imaged loci were first mapped to 200-kb resolution. All loci were either mapped to one or two 200-kb regions depending on their overlaps. If a locus was mapped to two regions, the features calculated from the models were averaged over these two regions in the analyses.

- **ChIP-seq (Histone marks)**

The signals and peaks for H3K36me3, H3K27me3, H3K9ac, H3K9me3, H3K27ac, H3K4me1, H3K4me2, H3K4me3, H3K79me2, H4K20me1, and H2AFZ histone modifications were downloaded from ENCODE[9,10]. Each region in the ChIP-seq data was mapped to 200-kb regions with an overlap of 50% or higher. After mapping, each 200-kb region had multiple ChIP-seq regions; therefore the signals mapped to each 200-kb region were averaged (we first took the inverse log2 of the signals, then averaged and took the log2 of the average value.), and the number of peaks mapped to each region were counted.

- **Subcompartments**

The definition of subcompartment states was retrieved from ref[2] which are at 100-kb resolution. For each 200-kb region of chromatin, we first mapped the subcompartment states and calculated their proportion. The state of the 200-kb region was then set to the majority of its constituent state

(50% or more). If there was no majority constituent state, we then assigned the region as NA and discarded them from further subcompartment-related analyses.

- **Compartments**

The definition of compartment states were retrieved from the 4DN data portal (Table S2) and ref[2] which are at 250-kb resolution. We mapped each compartment state to a 200-kb region with the maximum overlap. If there was no mapped state to a 200-kb region, then we assigned it as NA and discarded them from further compartment-related analyses.

- **LADs**

The definitions of LADs (constitutive and facultative LADs and inter-LADs) were retrieved from refs.[4,7]. LAD assignments were mapped to overlapping 200-kb regions. After mapping, if a 200-kb region had only one mapped LAD state, we assigned that state to the region. However, if the region had multiple mapped LAD states, we assigned the region as NA and discarded them from further LAD-related analysis. This mapping procedure resulted in 1304, 495, 1010, 1904 regions with cLAD, fLAD, ciLAD, and fiLAD assignments, respectively.

- **Enhancers/Superenhancers**

The definitions of enhancers (EN) and superenhancers (SEN) were retrieved from ref[13]. Each EN and SEN region was mapped to 200-kb regions with an overlap of 50% or higher. After mapping, we assigned regions as EN/SEN if they overlapped with one or more EN/SEN peaks. We assigned regions as NA if they did not overlap with any EN/SEN peaks and discarded them from further EN/SEN-related analysis.

## 3D DNA FISH *Experiments*

We carried out a set of 3-color FISH experiment where all probes were on chromosome 6: RP11-945M14 (306,712 – 532,406), RP11-1076L22 (130,076,074 – 130,287,007), and RP11-111J1 (87,193,201 – 87,351,481). For any particular chromosome domains (regions), multiple BAC clones were chosen and synthesized by Empire Genomics and tested individually for their specificity. The experiment was performed following the previous protocols[24,25]. GM12878 cells were cultured in a DMEM medium supplemented with 15% FBS, glutamine and penicillin/streptomycin as suggested by ENCODE. Two days before the experiment, 22mm x 22mm coverslips were cleaned and coated with L-poly-lysine (1mg/ml) at room temperature for 1-2 hours, and dried in a tissue culture hood after a brief rinse with sterile MilliQ water. On the day of the experiment, 10 million GM12878 cells were harvested by centrifugation at 100g for 10 minutes, resuspended in fresh culture medium ($3x10^6$ cells/ml), and seeded evenly on the coverslip in a 6-well tissue culture plate. After incubating at 37°C for one hour and briefly washing with PBS, the cells (on coverslips) were fixed with 4% freshly made paraformaldehyde (in 0.4x PBS) at room temperature for 10 minutes. The cell membrane was permeabilized firstly with 0.5% triton X100/1xPBS at room temperature for 20 minutes, and then through 4-5 freeze-and-thaw cycles (by dipping in liquid nitrogen and then thawing in room temperature) in the next day after pretreatment overnight with 20% glycerol/1xPBS and also before each dip. To facilitate access of the FISH probe to the chromatin DNA, the samples were each washed twice with 0.05% triton X100/1xPBS for five minutes, and then treated with 0.1N HCl at room temperature for 5-10 minutes to remove basic nuclear proteins. The HCl was removed from the sample followed by two washes with 0.05% triton X100/PBS and one wash with 2x SSC (diluted from 20xSSC: 3M

NaCl, 0.3M sodium citrate, pH 7.0) for 5-10 minutes each wash. The coverslips were then stored in 50% formamide/2x SSC at 4°C and were ready for the next step (good for two days to two months). The denaturation and hybridization steps were performed according to the protocols suggested by the manufacturer. (https://www.empiregenomics.com/files/store/products/FISH_probes/FISH_Protocol.pdf). The coverslips were brought to room temperature for 24 hours in advance before denaturing. On the day of experiment, fresh 70% formamide/2x SSC was prepared and pre-warmed at 73°C for 30 minutes. Cells on the coverslips were denatured in this solution (73°C) for five minutes, and dried through sequentially dipping into 70%, 85% and 100% ethanol one minute each at room temperature, and finally through evaporation at 45°C for 20 minutes. FISH probes were denatured similarly in 70% formamide/2x SSC for 5 minutes at 73°C and then quickly cooled down on ice. After incubating at 37°C for 10-20 minutes, three probes (150 ng each) for either targeted regions or for the three control regions were mixed thoroughly with 18 µl hybridization buffer (provided by the manufacturer), and applied evenly with the sample on a microscope slide. Hybridization of FISH probes with the samples occurred in a humidified chamber containing a paper towel soaked with 50% fomamide/ 2x SSC in dark at 37°C for 18-20 hours. Unbound FISH probes were removed by a series of washes, three times with 0.3% NP-40/0.4x SSC at 73°C for two minutes, each followed by a wash with 0.1% NP40/2x SSC at room temperature for one minute. After air-drying for five minutes in dark, the coverslips were mounted on microscope slide with 10 µl DAPI mounting solution and kept in dark at 4°C (ready for imaging).

The FISH images were acquired with Zeiss Laser Scanning Confocal microscope (LSC780) with 63x magnification oil immersion objective lenses. Cells are randomly chosen (each vision field contains 6-15 cells). Signals from four different fluorophores were obtained with two alternative frame scans for best separation: the first scan with two laser beams of 488 nm and 594 nm, followed by the second scan of 405 nm (for DAPI) and 532 nm laser beams (for the yellow probe used in targeted group) or 405 nm and 555 nm laser beams (for the orange probe used in control group). The minimal laser power was used in combination with appropriate filter settings (MBS 488/594 and MBS 458/514/561/633) to greatly reduce the signal bleed through between channels. Images of cells with optical Z sections from the bottom to the top with 0.25 µm or 0.3 µm intervals were acquired one section after another (frame scanning) with the software Zen provided by the manufacturer. Signals of each probe were stored in separate channels (four channels for three chromosomal regions plus DAPI staining of the whole chromosomal DNA).

The nucleus detection and distance measurements between probes were performed using the Nemo software for FISH image analyses[26]. Automated nucleus detection mode was used as the standard procedure, and additional manual selection followed when needed. Each of the cells was subject to manual inspection and validated for containing at least a set of the closest three probes.

## References


1. Yang, T. et al. HiCRep: assessing the reproducibility of Hi-C data using a stratum-adjusted correlation coefficient. *Genome Res* **27**, 1939-1949 (2017).
2. Rao, S.S. et al. A 3D map of the human genome at kilobase resolution reveals principles of chromatin looping. *Cell* **159**, 1665-80 (2014).
3. Chen, Y. et al. Mapping 3D genome organization relative to nuclear compartments using TSA-Seq as a cytological ruler. *J Cell Biol* **217**, 4025-4048 (2018).
4. Kind, J. et al. Genome-wide maps of nuclear lamina interactions in single human cells. *Cell* **163**, 134-47 (2015).
5. van Schaik, T., Vos, M., Peric-Hupkes, D., Hn Celie, P. & van Steensel, B. Cell cycle dynamics of lamina-associated DNA. *EMBO Rep* **21**, e50636 (2020).
6. Core, L.J. et al. Analysis of nascent RNA identifies a unified architecture of initiation regions at mammalian promoters and enhancers. *Nat Genet* **46**, 1311-20 (2014).
7. Girelli, G. et al. GPSeq reveals the radial organization of chromatin in the cell nucleus. *Nat Biotechnol* **38**, 1184-1193 (2020).
8. Pope, B.D. et al. Topologically associating domains are stable units of replication-timing regulation. *Nature* **515**, 402-5 (2014).
9. Consortium, E.P. An integrated encyclopedia of DNA elements in the human genome. *Nature* **489**, 57-74 (2012).
10. Davis, C.A. et al. The Encyclopedia of DNA elements (ENCODE): data portal update. *Nucleic Acids Res* **46**, D794-D801 (2018).
11. Osorio, D., Yu, X., Yu, P., Serpedin, E. & Cai, J.J. Single-cell RNA sequencing of a European and an African lymphoblastoid cell line. *Sci Data* **6**, 112 (2019).
12. Su, J.H., Zheng, P., Kinrot, S.S., Bintu, B. & Zhuang, X. Genome-Scale Imaging of the 3D Organization and Transcriptional Activity of Chromatin. *Cell* **182**, 1641-1659 e26 (2020).

13. Hnisz, D. et al. Super-enhancers in the control of cell identity and disease. *Cell* **155**, 934-47 (2013).
14. Duarte, M. & Watanabe, R.N. Notes on Scientific Computing for Biomechanics and Motor Control (Version v0.0.2). (http://doi.org/10.5281/zenodo.4599319, 2021).
15. Shin, H. et al. TopDom: an efficient and deterministic method for identifying topological domains in genomes. *Nucleic Acids Res* **44**, e70 (2016).
16. Levy-Leduc, C., Delattre, M., Mary-Huard, T. & Robin, S. Two-dimensional segmentation for analyzing Hi-C data. *Bioinformatics* **30**, i386-92 (2014).
17. Crane, E. et al. Condensin-driven remodelling of X chromosome topology during dosage compensation. *Nature* **523**, 240-4 (2015).
18. Serra, F. et al. Automatic analysis and 3D-modelling of Hi-C data using TADbit reveals structural features of the fly chromatin colors. *PLoS Comput Biol* **13**, e1005665 (2017).
19. Rohatgi, A. WebPlotDigitizer. 4.4 edn (https://automeris.io/WebPlotDigitizer, 2020).
20. Pedregosa, F. et al. Scikit-learn: Machine Learning in Python. *Journal of Machine Learning Research* **12**, 2825-2830 (2011).
21. Imakaev, M. et al. Iterative correction of Hi-C data reveals hallmarks of chromosome organization. *Nat Methods* **9**, 999-1003 (2012).
22. Hua, N. et al. Producing genome structure populations with the dynamic and automated PGS software. *Nat Protoc* **13**, 915-926 (2018).
23. Hinrichs, A.S. et al. The UCSC Genome Browser Database: update 2006. *Nucleic Acids Res* **34**, D590-8 (2006).
24. Dai, C. et al. Mining 3D genome structure populations identifies major factors governing the stability of regulatory communities. *Nat Commun* **7**, 11549 (2016).
25. Tjong, H. et al. Population-based 3D genome structure analysis reveals driving forces in spatial genome organization. *Proc Natl Acad Sci U S A* **113**, E1663-72 (2016).
26. Iannuccelli, E. et al. NEMO: a tool for analyzing gene and chromosome territory distributions from 3D-FISH experiments. *Bioinformatics* **26**, 696-7 (2010).
