# Supplementary material for: Evaluating the role of the nuclear microenvironment in gene function by population-based modeling
Source: Nat Struct Mol Biol. 2023 Aug 14;30(8):1193–206. doi: 10.1038/s41594-023-01036-1 (PMC10442234; doi:10.1038/s41594-023-01036-1)
Supplement: Supplementary file 2 — Reporting Summary [file 41594_2023_1036_MOESM2_ESM.pdf]

Corresponding author(s): Frank Alber

Last updated by author(s): 06/01/2023

Reporting Summary

Nature Portfolio wishes to improve the reproducibility of the work that we publish. This form provides structure for consistency and transparency in reporting. For further information on Nature Portfolio policies, see our Editorial Policies and the Editorial Policy Checklist.

Statistics

For all statistical analyses, confirm that the following items are present in the figure legend, table legend, main text, or Methods section.

n/a	Confirmed	
☐	☒	The exact sample size (*n*) for each experimental group/condition, given as a discrete number and unit of measurement
☒	☐	A statement on whether measurements were taken from distinct samples or whether the same sample was measured repeatedly
☐	☒	The statistical test(s) used AND whether they are one- or two-sided<br>*Only common tests should be described solely by name; describe more complex techniques in the Methods section.*
☒	☐	A description of all covariates tested
☒	☐	A description of any assumptions or corrections, such as tests of normality and adjustment for multiple comparisons
☐	☒	A full description of the statistical parameters including central tendency (e.g. means) or other basic estimates (e.g. regression coefficient) AND variation (e.g. standard deviation) or associated estimates of uncertainty (e.g. confidence intervals)
☐	☒	For null hypothesis testing, the test statistic (e.g. *F*, *t*, *r*) with confidence intervals, effect sizes, degrees of freedom and *P* value noted<br>*Give P values as exact values whenever suitable.*
☒	☐	For Bayesian analysis, information on the choice of priors and Markov chain Monte Carlo settings
☒	☐	For hierarchical and complex designs, identification of the appropriate level for tests and full reporting of outcomes
☐	☒	Estimates of effect sizes (e.g. Cohen's *d*, Pearson's *r*), indicating how they were calculated

Our web collection on statistics for biologists contains articles on many of the points above.

Software and code

Policy information about availability of computer code

Data collection	Populations of diploid genome structures were generated using the Integrative Genome Modeling (IGMv1.0) platform (www.github.com/alberlab/igm).
Data analysis	The analyses and most of the figure panels were performed using custom Python scripts (matplotlibv3.4, Scikit-learnv1.0, scipyv1.5 and networkxv2.3) together with the publicly available alabtools platform (https://github.com/alberlab/alabtools). The remaining panels and the final figures were assembled using Adobe Illustrator. Correlations between input and output contact matrices were calculated using HiCRep (https://github.com/TaoYang-dev/hicrep). Spatial partitions were identified using the MCL algorithm (https://micans.org/mcl/). Chromatin interaction networks were visualized with Cytoscape. Images of 3D genome structures were generated using UCSF Chimera1.13.

For manuscripts utilizing custom algorithms or software that are central to the research but not yet described in published literature, software must be made available to editors and reviewers. We strongly encourage code deposition in a community repository (e.g. GitHub). See the Nature Portfolio guidelines for submitting code & software for further information.

Data

Policy information about availability of data

All manuscripts must include a data availability statement. This statement should provide the following information, where applicable:

- Accession codes, unique identifiers, or web links for publicly available datasets
- A description of any restrictions on data availability
- For clinical datasets or third party data, please ensure that the statement adheres to our policy

The genome structure population and genome-wide structural features are available at https://doi.org/10.5281/zenodo.7352276. The accession codes for the experimental data used in our analyses are as follows. [GEO] GSE63525 (Hi-C), GSE63525 (subcompartments), GSE81553 (SON TSA-seq), GSE81553 (LaminB1 TSA-

seq), GSE56465 (single cell lamina DamID), GSM1480326 (GRO-seq), GSE135882 (GP-seq), GSM923451 (Repli-seq), GSM3596321 (scRNA-seq); [4DN] 4DNFIGL8MCSJ (laminB1 pA-DamID), 4DNFILYQ1PAY (compartments); [ENCODE]: ENCFF313LYI, ENCFF171MDW,ENCFF776DPQ, ENCFF309OEW, ENCFF028KBY, ENCFF601YET, ENCFF831ZHL, ENCFF039HDL, ENCFF340JIF, ENCFF803DJF, ENCFF683HCZ (ChIP-seq, histone modifications), https://zenodo.org/record/3928890 (DNA MERFISH imaging). The complete list of the datasets used in this study and their accession numbers are also tabulated in Supplementary Table S2.

Field-specific reporting

Please select the one below that is the best fit for your research. If you are not sure, read the appropriate sections before making your selection.

☒ Life sciences ☐ Behavioural & social sciences ☐ Ecological, evolutionary & environmental sciences

For a reference copy of the document with all sections, see nature.com/documents/nr-reporting-summary-flat.pdf

Life sciences study design

All studies must disclose on these points even when the disclosure is negative.

Sample size

The structure population contains 10,000 models. To test convergence with respect to population size, we generated 5 different populations with 50, 100, 1,000, 5,000 and 10,000 structures, always using the same computation protocol, and computed the correlation between the population predicted Hi-C contact probability matrix and the experimental input (see Supplementary Figure S3). The correlation values plateaus at N=1,000 structures. We then chose a sample size N = 10,000 structures to ensure convergence of the population predictive power, while simulating for a reasonable computing time.

Data exclusions

No structures were excluded from the data analysis.

Replication

Two technical replicates were calculated from different random starting configurations. Resulting contact frequency maps and the average radial positions of all chromatin regions between replica populations are nearly identical. All observed structural features discussed in this paper are reproduced in the technical replicate population.

Randomization

All our genome population calculations (via IGM) start out with fully randomized genome configurations.

Blinding

Blinding was effectively performed as identical analysis scripts were applied for all structure populations with no human intervention.

Reporting for specific materials, systems and methods

We require information from authors about some types of materials, experimental systems and methods used in many studies. Here, indicate whether each material, system or method listed is relevant to your study. If you are not sure if a list item applies to your research, read the appropriate section before selecting a response.

Materials & experimental systems

n/a	Involved in the study	
☒	☐	Antibodies
☒	☐	Eukaryotic cell lines
☒	☐	Palaeontology and archaeology
☒	☐	Animals and other organisms
☒	☐	Human research participants
☒	☐	Clinical data
☒	☐	Dual use research of concern

Methods

n/a	Involved in the study	
☒	☐	ChIP-seq
☒	☐	Flow cytometry
☒	☐	MRI-based neuroimaging
